# Mineralogical and chemical properties inversed from 21-lunar-day VNIS observations taken during the Chang’E-4 mission

**DOI:** 10.1038/s41598-021-93694-8

**Published:** 2021-07-29

**Authors:** Qinghong Zeng, Shengbo Chen, Yuanzhi Zhang, Yongling Mu, Rui Dai, Congyu Yang, Anzhen Li, Peng Lu

**Affiliations:** 1 College of Geo-Exploration Science and Technology, Jilin University, Changchun, China; 2 CAS Center for Excellence in Comparative Planetology, Hefei, China; 3 Key Laboratory of Lunar and Deep-Space Exploration, National Astronomical Observatories, Chinese Academy of Sciences, Beijing, China

**Keywords:** Mineralogy, Petrology

## Abstract

We report on the mineralogical and chemical properties of materials investigated by the lunar rover Yutu-2, which landed on the Von Kármán crater in the pre-Nectarian South Pole–Aitken (SPA) basin. Yutu-2 carried several scientific payloads, including the Visible and Near-infrared Imaging Spectrometer (VNIS), which is used for mineral identification, offering insights into lunar evolution. We used 86 valid VNIS data for 21 lunar days, with mineral abundance obtained using the Hapke radiative transfer model and sparse unmixing algorithm and chemical compositions empirically estimated. The mineralogical properties of the materials at the Chang’E-4 (CE-4) site referred to as norite/gabbro, based on findings of mineral abundance, indicate that they may be SPA impact melt components excavated by a surrounding impact crater. We find that CE-4 materials are dominated by plagioclase and pyroxene and feature little olivine, with 50 of 86 observations showing higher LCP than HCP in pyroxene. In view of the effects of space weathering, olivine content may be underestimated, with FeO and TiO_2_ content estimated using the maturity-corrected method. Estimates of chemical content are 7.42–18.82 wt% FeO and 1.48–2.1 wt% TiO_2_, with a low-medium Mg number (Mg # ~ 55). Olivine-rich materials are not present at the CE-4 landing site, based on the low-medium Mg #. Multi-origin materials at the CE-4 landing site were analyzed with regard to concentrations of FeO and TiO_2_ content, supporting our conclusion that the materials at CE-4 do not have a single source but rather are likely a mixture of SPA impact melt components excavated by surrounding impact crater and volcanic product ejecta.

## Introduction

**Figure 1:**
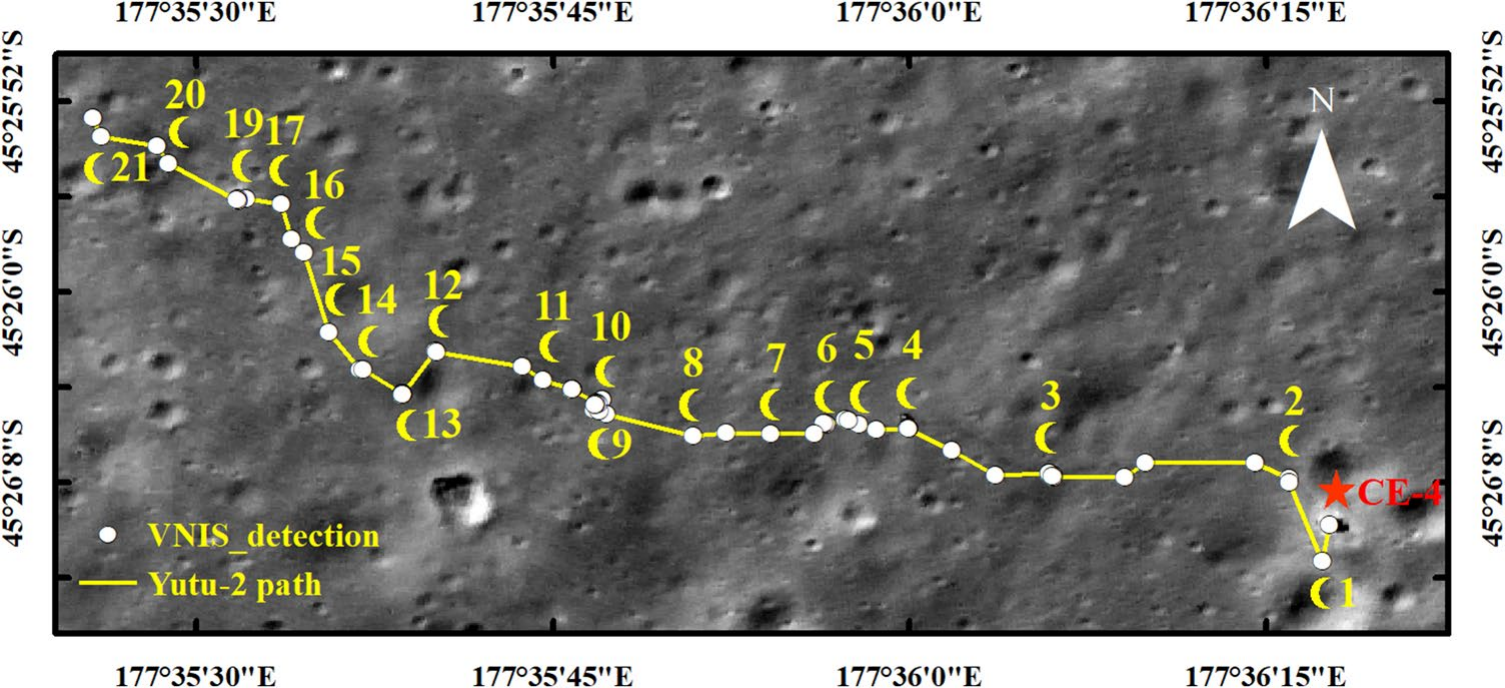
The routing path and the VNIS detections of the Yutu-2 rover during the 21 lunar days (except for the eighteenth lunar day when the Yutu-2 did not move). The number upper the point is the first VNIS detection of a new lunar day. The base map is from the LROC NAC image (image number M1311886645RC and M1311886645LC). This map was created in ESRI ArcMap 10.2 (https://desktop.arcgis.com/zh-cn/arcmap/).

Through October 25, 2020, the Yutu-2 rover of the Chang’E-4 (CE-4) mission had toured 519.29 m up to 600 days (for 21 lunar days), with the Visible and Near-Infrared Image Spectrometer (VNIS) on board the rover acquiring 106 in situ reflectance measurements (Fig. 1). This in situ scientific dataset provides a unique perspective from which to understand the mineralogical and chemical properties of the Von Kármán crater inside the South Pole–Aitken (SPA) basin on the far side of the moon^1,2^. A range of remote-sensing and in situ data, some based on spectral observations, analysis of geomorphology, and the subsurface structure within Von Kármán crater, have helped develop understandings of the moon’s history and evolution.

After the CE-4’s safe landing, researchers have reported initial results by interpreting the first lunar-day VNIS data showing that the materials along the rover route are dominated by olivine and low-calcium pyroxene (LCP)—suggesting that the Yutu-2 rover may have detected the moon’s mantle material^3,4^. These results are consistent with orbital data, confirming that the floor of the SPA is rich in mafic materials^5^.

However, additional data from the Yutu-2 rover have not indicated the presence of expected mantle materials at the CE-4 landing site, where materials are dominated by plagioclase (Plag) and pyroxene but include little olivine (OL), for the first three lunar days^6^. As exploration continues to produce more scientific data, we can arrive at new and different understandings. Several inversion methods could cause the controversial components found at the CE-4 landing site. Findings of magnesium-rich materials obtaining using the modified Gaussian method (MGM) may overstate olivine content, because plagioclase and olivine have very similar spectral features around 1250 nm, making it difficult to confirm the abundance of plagioclase by MGM deconvolution^7,8^. Further studies have considered additional minerals (such as plagioclase and agglutinates) for inversion through the Hapke radiation transmission model, but different unmixing methods (lookup tables^9^ and other empirical approaches^6^) may produce unexpected results. Accordingly, more lunar-days’ worth of data are needed to research mineralogical properties along the rover’s route and support use of an objective unmixing method based on radiative transfer models.

By contrast, the lunar surface material is affected mainly by space weathering, a natural process that affects spectral band depths^10^, hindering precise modeling of lunar mixtures^10,11^. Notably, various mature materials such as rock and regolith were found along the Yutu-2 rover’s route^6,7,12^. The regolith is more mature than the surrounding rocks^13^. Olivine and pyroxene, the major mafic minerals of lunar material, have distinctive spectral characteristics in the visible and near-infrared (VIS/NIS) and short-wave infrared (SWIR) regions^8,14^: for example, pyroxene exhibits absorption at ~ 1000 nm and ~ 2000 nm, whereas olivine has three overlaps in absorption near 1000 nm but no absorption at 2000 nm. Olivine and pyroxene differ in their sensitivity to space weathering^15^, making it difficult to ascertain the extent to which space weathering and mineral composition influence the spectrum. Thus chemical characteristics (such as iron and titanium content) of the lunar surface are needed to be concerned except for lunar minerals.

Iron and titanium are two elements of particular interest on the moon and are useful for understanding its origin and geological evolution. The major rock types can be distinguished by concentrations of FeO and TiO_2_ content^16^ supplemented and compared with the results of mineral inversion. Although optical maturity strongly influences spectral reflectance^10,17–19^, there are ways of reducing the effect of space weathering when inversion of FeO and TiO_2_ content. Generally speaking, two types of models are used, based on spectral data. One is based on the statistical relation of reflectance spectra and compositions, such as principal component analysis^20,21^, the support vector machine model^21^, and partial least squares regression^22^, mathematical methods that show potential for estimating element content by spectra. However, purely mathematical statistics are limited by mature differences in the training data, producing artifacts when applied to other regions. The other algorithms were maturity-corrected by parameterizing spectral properties of sensitivity to iron or titanium and soil maturity (e.g., Lucey et al. 1995^18^, 1998^16^, 2000^19^; Le Mouélic et al. 2000^23^), an approach that can normalize the effects of space weathering, optimized to remove mature effects. Thus FeO and TiO_2_ content and the optical maturity parameter (OMAT) of Yutu-2 spectral data can be estimated using Lucey et al.’s method^19^ to help explain the moon’s geological background.

In this study, we analyze in situ spectra observed by VNIS spectral data during the first 21 synodic days, with a view to reporting mineralogical and chemical information for the region of the Yutu-2 rover and offering insights into the nature and origin of the SPA basin. Based on analysis of the spectral data, we attempt to determine the mineralogical and chemical properties of the materials at the landing site.

## Results

### Mineralogy properties observed by Yutu-2 rover

**Figure 2:**
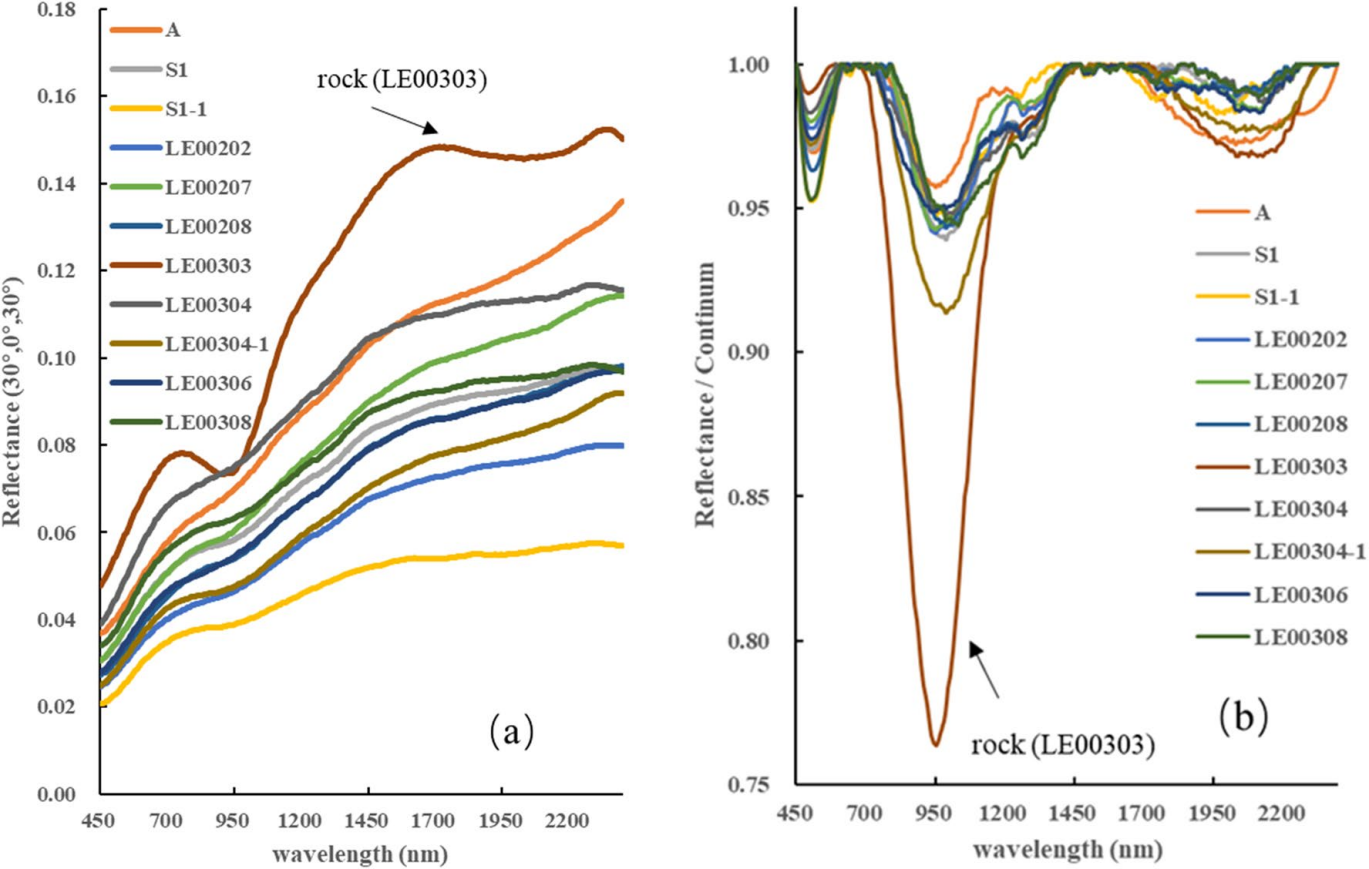
Examples of VNIS spectra measured by Yutu-2 rover. (**a**) The reflectance data with photometric correction and smoothing. (**b**) The spectra after continuum removed.

The VNIS spectra measured by the Yutu-2 rover show absorption regions around 1000 nm and 2000 nm (Fig. 2b). These absorption characteristics can be used to distinguish the primary minerals on the lunar surface, including pyroxene (PYX), olivine (OL), and plagioclase (Plag), because their absorption positions vary with Fe/Ca/Mg content^24–26^. As Fig. 2 shows, similarities in absorption peak position notwithstanding, the band depth of the rock (LE00303) is larger than for other nearby regolith, indicating similarities of components but differing degrees of space weathering.

**Figure 3:**
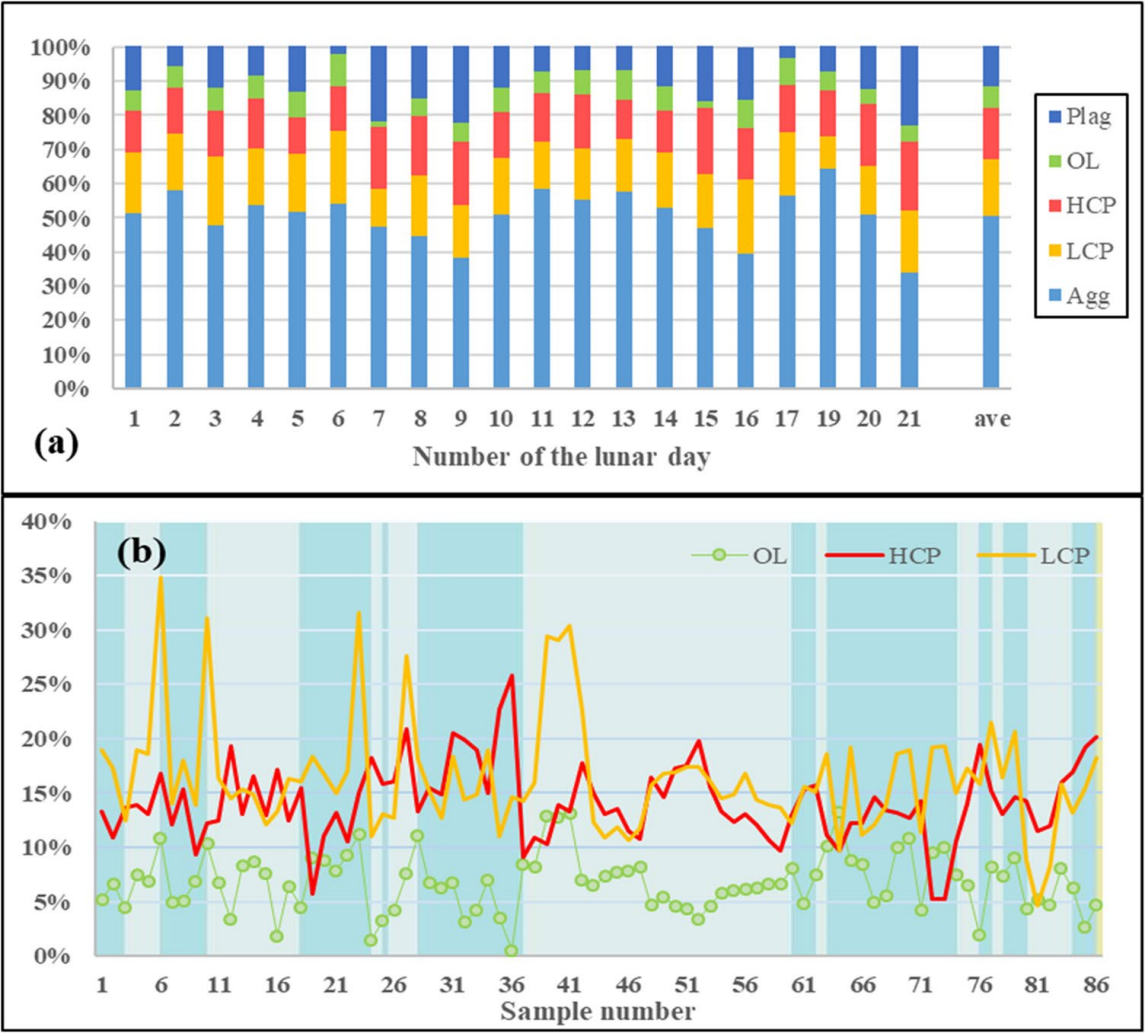
(**a**) The abundance of lunar materials during the first 21 lunar days. The estimated results show an average modal composition of 50.5% agglutinates (AGG), 16.3% LCP, 14.8% HCP, 6.1% olivine (OL) and 12.3% plagioclase (PLG). The ave means the average value during the first 21 lunar days. (**b**) The detailed abundance information for every VNIS detection during the first 21 lunar days. We use the background of the color bars to distinguish the observations during a lunar day.

To quantitatively infer the abundance of major minerals from the VNIS spectra measured by the Yutu-2 rover, we use the Hapke radiative model, in combination with a sparse unmixing algorithm, to estimate their relative content. In view of the space weathering effect, we add the agglutinates as the products of space weathering to estimate the relative contents of the primary minerals^27^. Our estimates show an average modeled composition of 50.5% agglutinates (AGG), 16.3% low-calcium pyroxene (LCP), 14.8% high-calcium pyroxene (HCP), 6.1% olivine (OL), and 12.3% plagioclase (PLG) (Fig. 3a). The modeled mineral abundance of the CE-4 observations suggests that their main sources are norite/gabbro and olivine norite/gabbro. Furthermore, we see higher LCP than HCP at most observation sites, consistent with the findings of the recent study^7^.

Figure 3b shows detailed abundance information for every VNIS detection. Most observations (50 of 86) show higher LCP concentrations than HCP, indicating that composition variation within a small area of the Yutu-2 route is consistent with that suggested by Huang et al.^7^ for the CE-4 landing region, based on multiple sources.

### Chemistry properties observed by Yutu-2 rover

**Figure 4:**
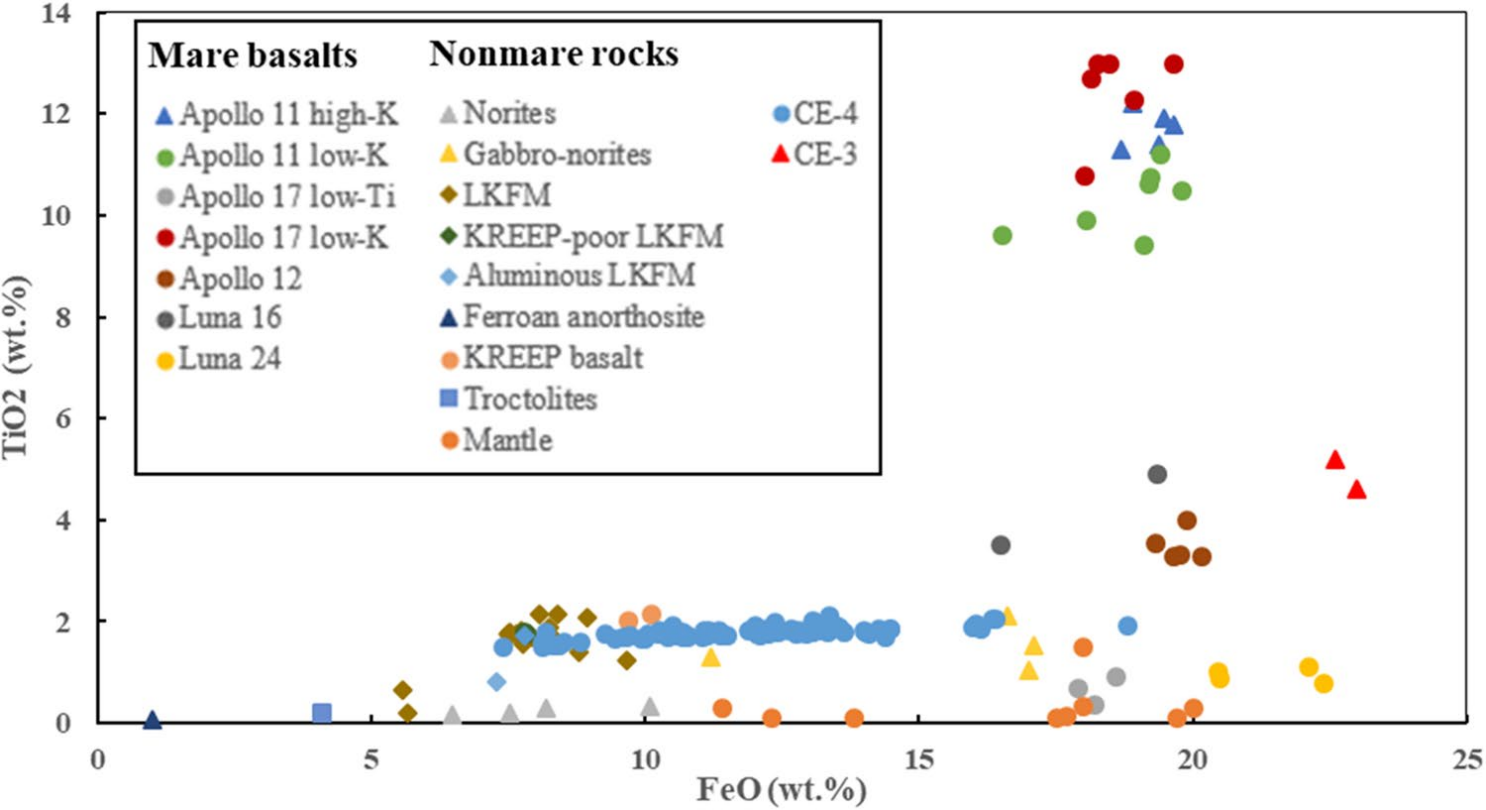
Chemical properties of CE-4 measured materials. The LKFM (11.4–20 wt% FeO, 0.1–0.34 wt% TiO_2_) is a rock type thought to compose the lower lunar crust. The lunar mantle (11.4–17.5 wt% FeO and 0.1–0.34 wt% TiO_2_) compositions are inferred by the experiments at high temperatures and pressure of mare basalt source regions in the lunar mantle which may lead to the elevation of TiO_2_. Mare basalts shows the range of 16.5–22.4 wt% FeO and 0.36–12.99 wt% TiO_2_.

From VNIS spectral data, adopting the method used by Luccy et al.^19^, we derived FeO and TiO_2_ content for Yutu-2 in situ measurements, with estimated content of 7.42–18.82 wt% FeO and 1.48–2.1 wt% TiO_2_ Fig. 4). FeO + TiO_2_ is consistent with estimates (11–19 wt%) based on remote sensing data^19,28,29^. The results show that surface materials have higher FeO content but lower TiO_2_ content.

Elaborating on the chemical properties of the CE-4 landing site, Fig. 4 plots CE-4 FeO versus TiO_2_ for major lunar rock type fields and CE-3 landing site materials. CE-3 data are from Ling et al.^30^, and lunar rock (mare basalt and nonmare rock) data are reported by Lucey et al.^16^, Lindstrom et al.^31^, and Papike et al.^32^ LKFM (low-K Fra Mauro), with basaltic impact melts thought to compose the lower lunar crust^33^. Figure 4 shows that CE-4 composition is unlike that of any major lunar rock type, but with the range of CE-4 spanning a variety of rock types—suggesting a complex source for CE-4. (For further discussion, see the next section.) What’s more, CE-4 composition differs from CE-3, with materials at the CE-4 landing site having lower FeO content than CE-3, indicating that materials at the CE-4 landing site are more mature, consistent with Is/FeO maturity indexes of ~ 82 for the CE-4 landing site^13^ and ~ 53 for CE-3^34^.

**Figure 5:**
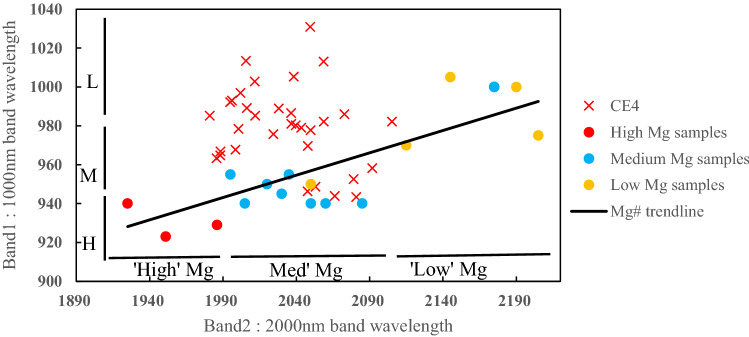
Comparison of the 1000 nm and 2000 nm band centers for spectra observed by Yutu-2 with the lunar highland and mare samples. The Mg # trend line was estimated using Apollo mare and highland samples. The Mg # of lunar samples were calculated using chemical information of MgO and FeO.

Mg number (Mg # = mole percent MgO/(MgO + FeO)) is one of the chemical indexes used to discuss the origin of lunar samples. The Mg number (Mg # = mole percent MgO/(MgO + FeO)) at the CE-4 site was empirically estimated by the 1000-nm and 2000-nm band centers, as Fig. 5 shows. We seek to identify mafic compositional trends and assess whether they are related to band centers, with the magnesium ratio classified as high (Mg # > 75), medium (Mg # 50–75), or low (Mg # < 50). The materials at the CE-4 site have a relatively low–medium Mg # (Fig. 5), consistent with the Mg # of ~ 55 derived from the Chang’E-1 Imaging Interferometer (IIM) data^29^.

## Discussions

Space weathering introduces changes to visible and near-infrared reflectance spectra^35^: because the moon is an airless body, its surface is altered through irradiation by solar wind and galactic cosmic rays^36^, a process that reduces the strength of mineralogical absorption bands, including the mafic minerals pyroxene and olivine. Various types of pits are on the path of the Yutu-2, and the fragments that fill them show varying degrees of space weathering (Fig. S1), providing a unique perspective on space weathering’s influence on mineral abundance, based on in situ spectra on the far side of the moon.

**Figure 6:**
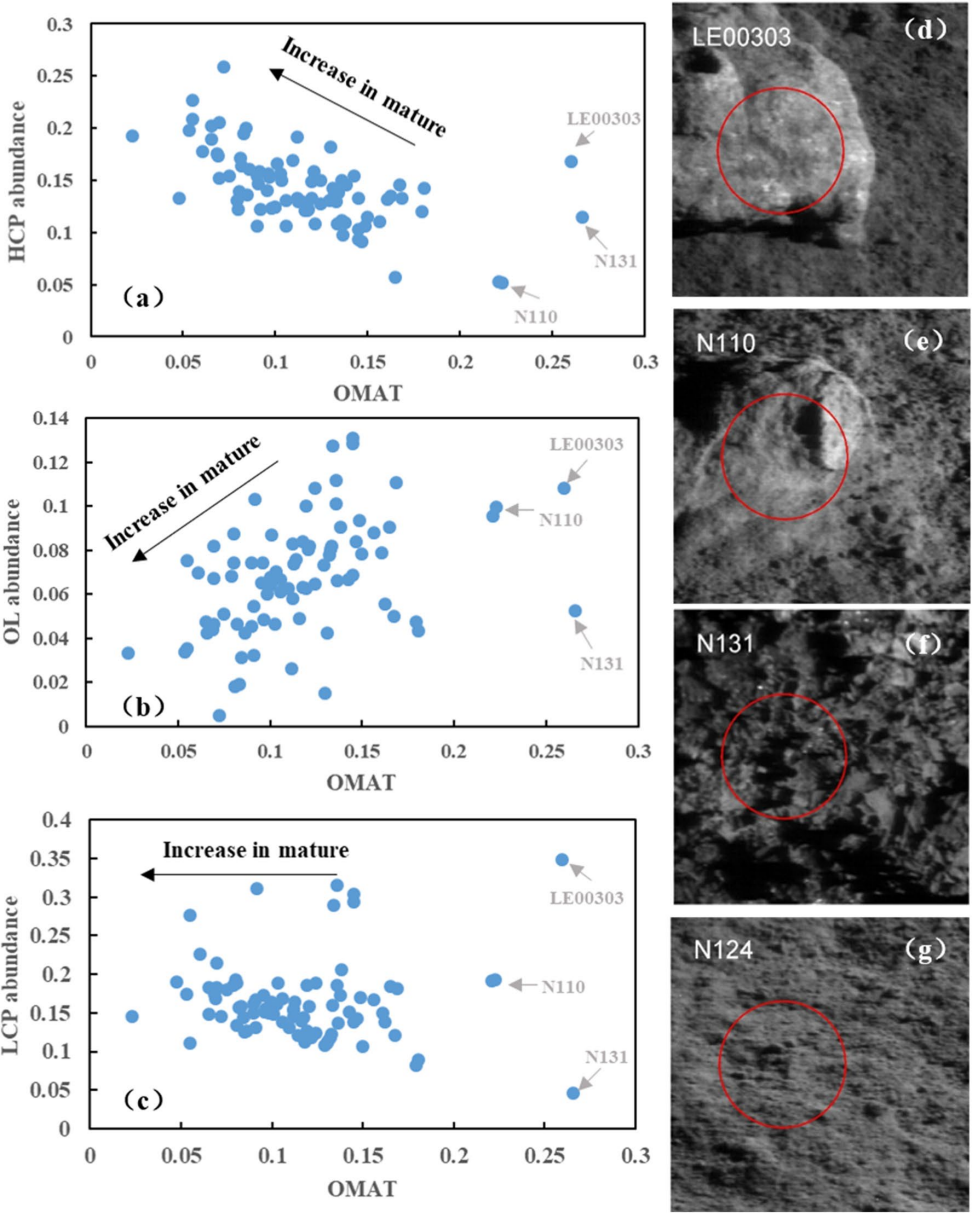
The relation between the OMAT and the main lunar minerals (HCP, LCP and OL). (**a**–**c**) are the scatterplots showing the relationship between the OMAT and the main lunar materials. (**d**–**g**) are the different type of lunar materials during the CE-4 routine path, (**d**, **e**) are the rock types, (**f**) is the fragment filled in the pit, (**g**) is the normal type of the lunar regolith nearby the Yutu-2, the red circle is the field of view (FOV) for SWIR. The VNIS images (**d**–**g**) were created by our own script in MATLAB 2013a (https://www.mathworks.com/products/matlab.html).

Optical maturity (OMAT) is a spectral parameter used to estimate the degree of space weathering for lunar soils, based on reflectance properties. We calculated the optical maturity parameter^19^ and produced a scatterplot (Fig. 6) of the relationship between OMAT and lunar mafic materials (HCP, LCP, and OL), showing that the three minerals have different weathering resistances. OL has decreased tendency with increasing maturity, whereas OL has weak resistance to weathering. LCP has better weathering resistance, whereas HCP shows relative increases with maturity, because they are in the relative content system, indicating that HCP has better weathering resistance. Because of the effects of space weathering, olivine may be underestimated, so further discussion of the sources of materials at CE-4 should be combined with the chemical properties.

The CE-4 landing site is in the Von Kármán crater, which formed in the pre-Nectarian era and subsequently underwent a series of complex geological evolutions^37–40^. The materials at the CE-4 landing site measured by Yutu-2 are dominated by pyroxenes and plagioclase, with little olivine seen—suggesting that the rock type is norite/gabbro. The norite-like surface materials at the CE-4 landing site may contain materials of the noritic layer during SPA melt pool formation^41,42^, excavated by the surrounding impact craters (e.g., Finsen crater and Alder crater). Furthermore, no olivine-rich materials were found at the CE-4 landing site with low–medium Mg #, suggesting that the source might not be the lunar mantle, although the possibility of the mixture’s coming from the lower crust cannot be ruled out.

By contrast, elevated CE-4 FeO content (Fig. 4) may result from exposure from lunar mantle^16^, but TiO_2_ content is significantly higher than the expected mantle composition. The correlation of FeO with TiO_2_ of CE-4 shown in Fig. 4 could result from the differentiation of the large melt sheet, with the differentiation materials perhaps containing parts of LKFM (lower crustal composition).

The CE-4 materials have a distinctive chemical characteristic compared with other various mare basalt (Fig. 4), and the chemical composition at CE-4 may offer insights into the mixing effects of impact melt ejecta and volcanic products.

**Figure 7:**
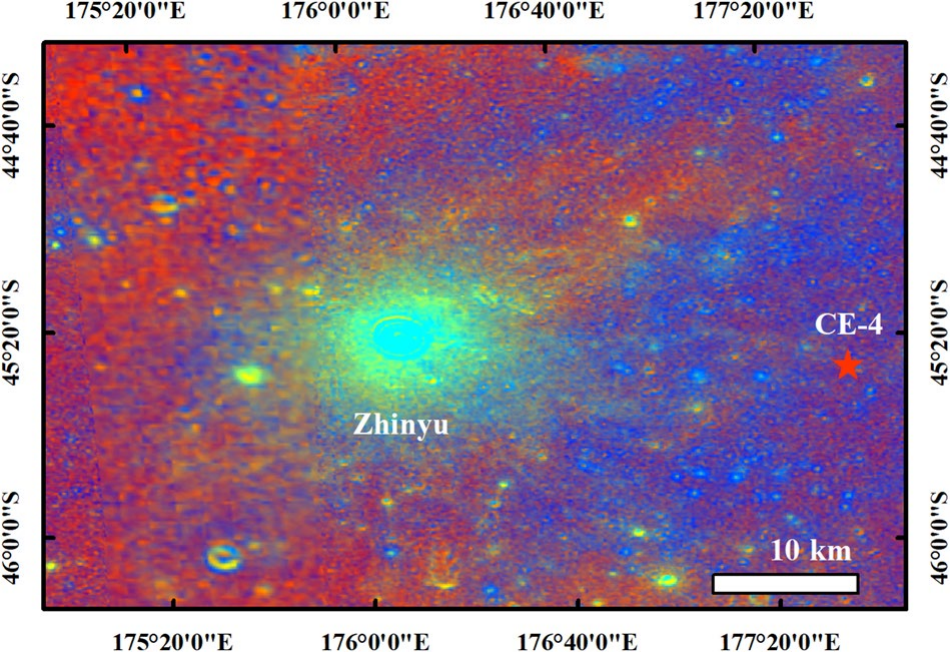
The color ratio image^43,44^ from Clementine UVVIS data at the CE-4 landing site, showing a slight impact ray to the CE-4 landing site (red star) from the Zhinyu crater. The map was created in ESRI ArcMap 10.2 (https://desktop.arcgis.com/zh-cn/arcmap/).

We note multiple lava-infilling events^45^ within the Von Kármán crater, ~ 70 km west of the CE-4 landing site. Volcanic activity may have peaked in the Late Imbrium and could have continued into the Eratosthenian epoch^45^, with multiple lava flooding products excavated by the fresh Zhinyu crater (~ 4 km diameter), ~ 30 km west of the CE-4 landing site^12^. The orbital data indicate the presence of a slight impact ray to the CE-4 landing site from the Zhinyu crater, based on color ratio imaging from Clementine UVVIS data (Fig. 7).

As already discussed, the CE-4 landing site has been affected by the mixing effects of ejecta and volcanic products. Accordingly, multiple lavas were probably infilled in the bottom of Zhinyu crater and the CE-4 landing site before the Zhinyu impact event, containing the ejecta and volcanic products from the Zhinyu crater brought to the surface of the CE-4 landing site.

This paper investigates the mineralogical and chemical properties by in situ spectra as measured by Yutu-2, finding that the materials detected by Yutu-2 were norite/gabbro. No olivine-rich mantle materials^3^ have been detected thus far, but plagioclase and pyroxene have been. Agglutinates are abundant, based on the results of mineral abundance, suggesting that the materials have undergone space weathering effects and reached a mature state. The chemical compositions of materials at the CE-4 landing site were empirically estimated using in situ spectra. Chemical properties show that the surface materials have higher FeO (7.42–18.82 wt%) and lower TiO_2_ (1.48–2.1 wt%) content and low–medium Mg number (Mg #  ~ 55). Based on analysis of their mineralogical and chemical properties, the materials measured by Yutu-2 likely originated from SPA impact melt components excavated by the surrounding impact crater and from the mixing effects of SPA ejecta and volcanic products.

## Methods

### VNIS spectra

The Visible and Near-infrared Imaging Spectrometer (VNIS) is composed of a 256- by 256-pixel VIS/NIR imager and a short-wave infrared (SWIR) single-pixel non-imaging spectrometer^3,14^. The VNIS is fixed on the front of the rover, offering a 45° viewing angle at a height of 0.69 m. Its spectral range is 450–2395 nm, with 400 spectral channels at a default 5-nm resolution. The VIS/NIR imager works from 450 to 945 nm, with 300 channels, and the 100-channel SWIR spectrometer works from 900 to 2395 nm, with 10 overlap channels^1,14^. The SWIR spectrometer’s field of view is circular, with a center of (96, 128) in the VIS/NIR image and a diameter of 107 pixels.

### VNIS data processing

This section describes VNIS data processing. VNIS spectral data processing began with level 2B data, which underwent dark current, flat-field, temperature corrections and radiometric and geometric calibration^3,46–48^. L2B radiance data were converted to reflectance data by solar irradiance, for an airless planetary surface, with bidirectional reflectance commonly given as radiance factor (RADF) I/F,

1 $$\mathrm{I}/\mathrm{F}({\lambda }_{i})=\frac{\pi {I}_{s}({\lambda }_{i})}{\int J(\lambda )S(\lambda )d\lambda }$$

where $J(\lambda )$ is the solar irradiance^49^, $S(\lambda )$ the spectral response of the VNIS sensor, and ${I}_{s}({\lambda }_{i})$ the radiance measured by Yutu-2 at wavelength ${\lambda }_{i}$.

To reduce the influence of the geometric circumstances, the photometric correction described in Lin et al.^6^ was used to correct the data to fit common viewing geometry (incidence angle = 30°, emission angle = 0°, phase angle = 30°).

Owing to differences in signal-to-noise ratios (SNR) between the VIS/NIR spectrum (37.4 dB) and SWIR spectrum (44.0 dB)^8^, a gap is seen at the boundary of the two spectra. With no obvious slope difference at the overlapping region (900–950 nm), and taking into account SWIR’s higher SNR, we multiplied the VIS/NIR spectrum by a factor (reflectance ratio of SWIR 900-nm to the VNIS 900-nm) to connect to the SWIR spectrum and obtain a continuous spectrum. Finally, Savitzky–Golay method was performed during the spectral data smoothing, shown in Fig. 2.

### Estimation of absorption band center

In lunar mineralogical studies, the absorption band center is an important spectral parameter^50^, especially for mafic minerals (olivine and pyroxene), which have two obvious absorption bands, band1 (~ 1000 nm) and band2 (~ 2000 nm). The properties of absorption band centers are related to mineral structure and chemical composition^51^.

To calculate the band center, we first used the straight-line continuum method^52^ to obtain the continuum-removed spectra. We then used a sixth-order polynomial to fit the bottom of the continuum-removed absorption feature around band1 (~ 1000 nm) and band2 (~ 2000 nm)^48^. The band center is defined as the wavelength at the minimum point on the polynomial fitted curve. We set three different spectral ranges for band1 and band2 when polynomial fitting, and take the average center values as the final results.

### Estimation of the mineral abundances from the spectra

In this study, we used the radiative transfer model^53,54^ (see Supplementary Text S1) and the sparse unmixing algorithm^55,56^ to estimate mineral abundance from the VNIS spectra. To avoid any thermal emission effects, we limited our analysis to the wavelength range 950–1500 nm. First we calculated the imaginary part (k) of the endmembers (shown in Table S1) using the Hapke model. Next we computed the single scattering (SSA) for each endmember with different particle sizes (ranging from 5 to 200 nm, with an interval of 5 nm) using the Hapke slab model, constructed as the endmember library. Finally, we used the sparse unmixing algorithm to unmix the SSA of the Yutu-2 spectra derived by the Hapke model.

### Derivation of iron and titanium abundance

To evaluate the chemical properties of the materials detected by Yutu-2, we calculated FeO and TiO_2_ content using the algorithms described by Luccy et al.^19^. These approaches were based on returned *Apollo* and *Luna* samples. FeO and TiO_2_ content was calculated from the best fit curve,

2 $$ {\text{FeO}}\;({\text{wt}}{\text{\% }}) = 31.875\theta _{{Fe}}  - 17.895 $$

3 $$ \theta _{{Fe}}  =  - {\text{arctan}}[\left( {R_{{950}} /R_{{750}}  - y_{{0Fe}} } \right)/\left( {R_{{750}}  - x_{{0Fe}} } \right) $$

4 $$ {\text{TiO}}_{2} \;(wt\% ) = 1.836 \times \theta _{{Ti}} ^{{9.779}} $$

5 $$ \theta _{{Ti}}  = {\text{arctan}}\left[ {\left( {R_{{415}} /R_{{750}}  - y_{{0Ti}} } \right)/\left( {R_{{750}}  - x_{{0Ti}} } \right)} \right] $$

where the origin ${(x}_{0Fe},{y}_{0Fe})$ and $({x}_{0Ti},{y}_{0Ti}$) were set to (–0.1, 1.39) and (–1.08, 0.208), optimized to maximize the correlation coefficient between content and ${\theta }_{Fe}$ using the *Apollo* lunar samples^10,57^. ${R}_{415}$ was determined by ${R}_{415}={0.95R}_{415}-0.0013$ (${R}^{2}=0.99$).

## Supplementary Information

Supplementary Information 1.

Supplementary Information 2.

## Data Availability

CE-4 VNIS data are available at Data Publishing and Information Service System of China’s Lunar Exploration Program (http://moon.bao.ac.cn/). All the CE-4 VNIS data IDs are listed in Supplementary Table S3. Additional data related to this paper are available from the corresponding author upon reasonable request.

## References

[1] Li CL, Xu R, Lv G, Yuan LY, He ZP, Wang JY (2019). Detection and calibration characteristics of the visible and near-infrared imaging spectrometer in the Chang'E-4. Rev. Sci. Instrum..

[2] Chen J, Ling ZC, Qiao L, He ZP, Xu R, Sun LZ, Zhang J, Li B, Fu XH, Liu CQ, Qi XB (2020). Mineralogy of Chang'E-4 landing site: Preliminary results of visible and near-infrared imaging spectrometer. Sci. China Inform. Sci..

[3] Li CL, Liu DW, Liu B, Ren X, Liu JJ, He ZP, Zuo W, Zeng XG, Xu R, Tan X, Zhang XX, Chen WL, Shu R, Wen WB, Su Y, Zhang HB, Ouyang ZY (2019). Chang'E-4 initial spectroscopic identification of lunar far-side mantle-derived materials. Nature.

[4] Gou S, Di KC, Yue ZY, Liu ZQ, He ZP, Xu R, Liu B, Peng M, Wan WH, Wang YX, Liu JZ (2020). Forsteritic olivine and magnesium-rich orthopyroxene materials measured by Chang'E-4 rover. Icarus.

[5] Moriarty DP, Pieters CM (2018). The character of South Pole-Aitken Basin: Patterns of surface and subsurface composition. J. Geophys. Res. Planet.

[6] Lin HL, He ZP, Yang W, Lin YT, Xu R, Zhang C, Zhu MH, Chang R, Zhang JH, Li CL, Lin HY, Liu Y, Gou S, Wei Y, Hu S, Xue CB, Yang JF, Zhong J, Fu XH, Wan WX, Zou YL (2020). Olivine-norite rock detected by the lunar rover Yutu-2 likely crystallized from the SPA-impact melt pool. Natl. Sci. Rev..

[7] Huang J, Xiao ZY, Xiao L, Horgan B, Hu XY, Lucey P, Xiao X, Zhao SY, Qian YQ, Zhang H, Li CL, Xu R, He ZP, Yang JF, Xue B, He Q, Zhong J, Lin HY, Huang CN, Xie JF (2020). Diverse rock types detected in the lunar South Pole-Aitken Basin by the Chang'E-4 lunar mission. Geology.

[8] Gou S, Di KC, Yue ZY, Liu ZQ, He ZP, Xu R, Lin HL, Liu B, Peng M, Wan WH, Wang YX, Liu JZ (2019). Lunar deep materials observed by Chang'E-4 rover. Earth Planet. Sci. Lett..

[9] Hu XY, Ma P, Yang YZ, Zhu MH, Jiang T, Lucey PG, Sun LZ, Zhang H, Li CL, Xu R, He ZP, Lin HY, Huang CN, Sun YX (2019). Mineral abundances inferred from in situ reflectance measurements of Chang'E-4 landing site in South Pole-Aitken Basin. Geophys. Res. Lett..

[10] Pieters CM, Taylor LA, Noble SK, Keller LP, Hapke B, Morris RV, Allen CC, McKay DS, Wentworth S (2000). Space weathering on airless bodies: Resolving a mystery with lunar samples. Meteor. Planet. Sci..

[11] Moriarty DP, Pieters CM (2018). The character of South Pole-Aitken Basin: Patterns of surface and subsurface composition. J. Geophys. Res. Planets.

[12] Ma P, Sun YX, Zhu MH, Yang YZ, Hu XY, Jiang T, Zhang H, Lucey PG, Xu R, Li CL, He ZP, Xue B, Yang JF, Huang CN, Lin HY (2020). A plagioclase-rich rock measured by Yutu-2 Rover in Von Karman crater on the far side of the Moon. Icarus.

[13] Gou S, Yue Z, Di K, Wan W, Liu Z, Liu B, Peng M, Wang Y, He Z, Xu R (2020). In situ spectral measurements of space weathering by Chang'E-4 rover. Earth Planet. Sci. Lett..

[14] He ZP, Li CL, Xu R, Lv G, Yuan LY, Wang JY (2019). Spectrometers based on acousto-optic tunable filters for in situ lunar surface measurement. J. Appl. Remote Sens..

[15] Weber I, Stojic AN, Morlok A, Reitze MP, Markus K, Hiesinger H, Pavlov SG, Wirth R, Chreiber A, Sohn M, Hubers HW, Helbert J (2020). Space weathering by simulated micrometeorite bombardment on natural olivine and pyroxene: A coordinated IR and TEM study. Earth Planet. Sci. Lett..

[16] Lucey PG, Taylor GJ, Hawke BR, Spudis PD (1998). FeO and TiO2 concentrations in the South Pole-Aitken basin: Implications for mantle composition and basin formation. J. Geophys. Res. Atmos..

[17] Fischer EM, Pieters CM (1994). Remote determination of exposure degree and iron concentration of lunar soils using VIS-NIR spectroscopic methods. Icarus.

[18] Lucey PG, Taylor GJ, Malaret E (1995). Abundance and distribution of iron on the Moon. Science.

[19] Lucey PG, Blewett DT, Jolliff BL (2000). Lunar iron and titanium abundance algorithms based on final processing of Clementine ultraviolet-visible images. J. Geophys. Res..

[20] Pieters CM, Stankevich DG, Shkuratov YG, Taylor LA (2002). Statistical analysis of the links among lunar mare soil mineralogy, chemistry, and reflectance spectra. Icarus.

[21] Zhang XY, Li CL, Lü C (2009). Quantification of the chemical composition of lunar soil in terms of its reflectance spectra by PCA and SVM. Chin. J. Geochem..

[22] Li, L. Partial least squares modeling to quantify lunar soil composition with hyperspectral reflectance measurements. *J. Geophys. Res.* **111**(E4), pp E04002 (2006).

[23] Mouélic SL, Langevin Y, Erard S, Pinet P, Chevrel S, Daydou Y (2000). Discrimination between maturity and composition of lunar soils from integrated Clementine UV-visible/near-infrared data: Application to the Aristarchus Plateau. J. Geophys. Res..

[24] Adams, J. B. & Goullaud, L. H. Plagioclase feldspars: Visible and near infrared diffuse reflectance spectra as applied to remote sensing. *Geochim. Cosmochim. Acta* **10** (1978).

[25] Bowell E (1988). Application of photometric models to asteroids. Asteroids.

[26] Sunshine JM, Pieters CM (1998). Determining the composition of olivine from reflectance spectroscopy. J. Geophys. Res..

[27] Trang D, Lucey PG (2019). Improved space weathering maps of the lunar surface through radiative transfer modeling of Kaguya multiband imager data. Icarus.

[28] Otake, H., Ohtake, M. & Hirata, N. Lunar iron and titanium abundance algorithms based on SELENE (Kaguya) multiband imager data. In *43rd Lunar and Planetary Science Conference*, Vol. 43, 1905 (2012).

[29] Ling Z, Qiao L, Liu C, Cao H, Bi X, Lu X, Zhang J, Fu X, Li B, Liu J (2019). Composition, mineralogy and chronology of mare basalts and non-mare materials in Von Kármán crater: Landing site of the Chang'E-4 mission. Planet. Space Sci..

[30] Ling Z, Jolliff BL, Wang A, Li C, Liu J, Zhang J, Li B, Sun L, Chen J, Xiao L (2015). Correlated compositional and mineralogical investigations at the Chang’E-3 landing site. Nat. Commun..

[31] Lindstrom, M. M., Marvin, U. B., Holmberg, B. B. & Mittlefehldt, D. W. Apollo 15 KREEP-poor impact melts. In *Lunar and Planetary Science Conference 20th* (1989).

[32] Papike JJ, Ryder G, Shearer CK (1998). Lunar samples. Rev. Mineral. Geochem..

[33] Spudis PD (1984). Apollo 16 site geology and impact melts: Implications for the geologic history of the lunar highlands. J. Geophys. Res. Solid Earth.

[34] Xiao L, Zhu P, Fang G, Xiao Z, Zou Y, Zhao J, Zhao N, Yuan Y, Le Qiao L, Zhang X (2015). A young multilayered terrane of the northern Mare Imbrium revealed by Chang'E-3 mission. Science.

[35] Pieters CM, Noble SK (2016). Space weathering on airless bodies. J. Geophys. Res. Planets.

[36] Brett WD, Robinson MS, Boyd AK, Sato H, Hapke BW, Hawke BY (2014). Characterization of space weathering from lunar reconnaissance orbiter camera ultraviolet observations of the Moon. J. Geophys. Res. Planets.

[37] Fu XH, Qiao L, Zhang J, Ling Z, Li B (2020). The subsurface structure and stratigraphy of the Chang'E-4 landing site: Orbital evidence from small craters on the Von Kármán crater floor. Res. Astron. Astrophys..

[38] Huang J, Xiao Z, Flahaut J, Martinot M, Head J, Xiao X, Xie M, Xiao L (2018). Geological characteristics of Von Kármán Crater, Northwestern South Pole-Aitken Basin: Chang'E-4 landing site region. J. Geophys. Res. Planets.

[39] Paskert JH, Hiesinger H, van der Bogert CH (2018). Lunar far side volcanism in and around the south pole–Aitken basin. Icarus.

[40] Wilhelms, D. E., Howard, K. A. & Wilshire, H. G. Geologic map of the south side of the Moon. In *IMAP* (1979).

[41] Hurwitz DM, Kring DA (2014). Differentiation of the South Pole-Aitken basin impact melt sheet: Implications for lunar exploration. J. Geophys. Res. Planets.

[42] Vaughan WM, Head JW (2014). Impact melt differentiation in the South Pole-Aitken basin: Some observations and speculations. Planet. Space Sci..

[43] McEwen AS, Robinson MS (1997). Mapping of the Moon by Clementine. Adv. Space Res..

[44] Pieters CM, Tompkins S (1999). Tsiolkovsky crater: A window into crustal processes on the lunar farside. J. Geophys. Res..

[45] Lai JL, Xu Y, Roberto B, Meng X, Xiao L, Xie MG, Liu B, Di KC, Zhang XP, Zhou B, Shen SX, Xu LY (2020). First look by the Yutu-2 rover at the deep subsurface structure at the lunar farside. Nat. Commun..

[46] He ZP, Wang BY, Lv G, Li CL, Yuan LY, Xu R, Chen K, Wang JY (2014). Visible and near-infrared imaging spectrometer and its preliminary results from the Chang'E 3 project. Rev. Sci. Instrum..

[47] Liu B, Liu J, Zhang G, Ling Z, Zhang J, He Z, Yang B, Zou Y (2013). Reflectance conversion methods for the VIS/NIR imaging spectrometer aboard the Chang'E-3 lunar rover: based on ground validation experiment data. Res. Astron. Astrophys..

[48] Wu Y, Hapke B (2018). Spectroscopic observations of the Moon at the lunar surface. Earth Planet. Sci. Lett..

[49] Gueymard C (2004). Direct solar transmittance and irradiance predictions with broadband models. Part I: detailed theoretical performance assessment. Sol. Energy.

[50] Zhang X, Ouyang ZY, Zhang X, Chen Y, Tang X, Xu A, Tang Z, Wu Y (2016). Study of the continuum removal method for the Moon Mineralogy Mapper (M3) and its application to Mare Humorum and Mare Nubium. Res. Astron. Astrophys..

[51] Klima RL, Pieters CM, Boardman JW, Green RO, Head JW, Isaacson PJ, Mustard JF, Nettles JW, Petro NE, Staid MI, Sunshine JM, Taylor LA, Tompkins S (2011). New insights into lunar petrology: Distribution and composition of prominent low-Ca pyroxene exposures as observed by the Moon Mineralogy Mapper (M3). J. Geophys. Res. Planets.

[52] Clark RN, Roush TL (1984). Reflectance spectroscopy: Quantitative analysis techniques for remote sensing applications. J. Geophys. Res. Solid Earth.

[53] Hapke B (1981). Bidirectional reflectance spectroscopy: 1. J. Geophys. Res..

[54] Hapke B (2012). Theory of Reflectance and Emittance Spectroscopy.

[55] Iordache M-D, Bioucas-Dias JM, Plaza A (2011). Sparse unmixing of hyperspectral data. IEEE Trans. Geosci. Remote Sens..

[56] Lin H, Zhang X, Yang Y, Guo D, Wu X, Qi W (2019). Retrieval of the mineral abundance and particle size distribution at the landing site of Yutu rover with hyperspectral remote sensing data (Article). J. Remote Sens..

[57] Taylor LA, Pieters CM, Keller LP, Morris RV, McKay DS (2001). Lunar mare soils: Space weathering and the major effects of surface-correlated nanophase Fe. J. Geophys. Res..

