# Publisher Correction: Mineralogical and chemical properties inversed from 21-lunar-day VNIS observations taken during the Chang’E-4 mission

**DOI:** 10.1038/s41598-021-97274-8

**Published:** 2021-08-31

**Authors:** Qinghong Zeng, Shengbo Chen, Yuanzhi Zhang, Yongling Mu, Rui Dai, Congyu Yang, Anzhen Li, Peng Lu

**Affiliations:** 1 College of Geo-Exploration Science and Technology, Jilin University, Changchun, China; 2 CAS Center for Excellence in Comparative Planetology, Hefei, China; 3 Key Laboratory of Lunar and Deep-Space Exploration, National Astronomical Observatories, Chinese Academy of Sciences, Beijing, China

Correction to: *Scientific Reports* https://doi.org/10.1038/s41598-021-93694-8, published online 29 July 2021.

The original version of this Article omitted an affiliation for Shengbo Chen. The correct affiliations for Shengbo Chen are listed below:

College of Geo-Exploration Science and Technology, Jilin University, Changchun, China.

CAS Center for Excellence in Comparative Planetology, Hefei, China.

The original Article has been corrected.

