# Supplementary figures and images for: Mineralogical and chemical properties inversed from 21-lunar-day VNIS observations taken during the Chang’E-4 mission
Source: Sci Rep. 2021 Jul 29;11:15435. doi: 10.1038/s41598-021-93694-8 (PMC8322253; doi:10.1038/s41598-021-93694-8)

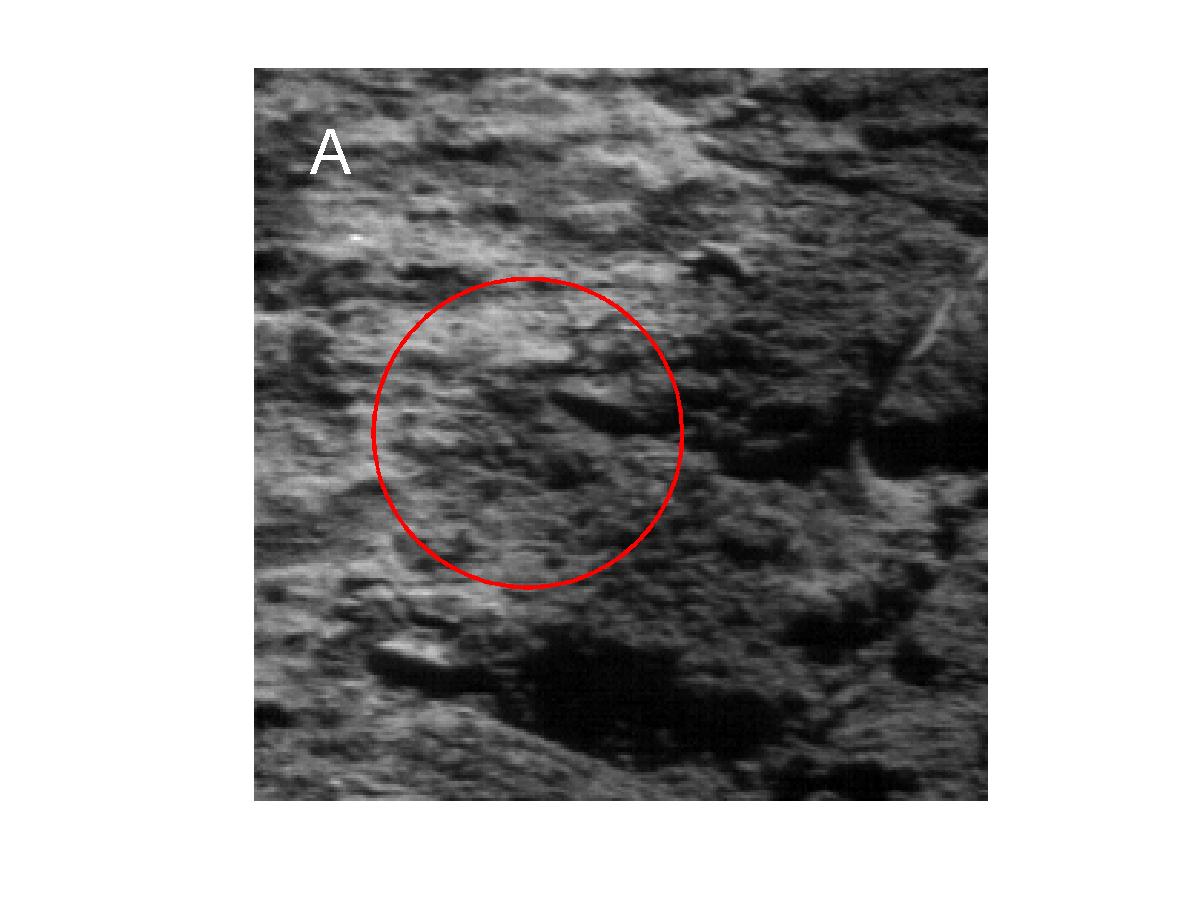

Supplement: Supplementary file 1 — Supplementary Information 1. [file 41598_2021_93694_MOESM1_ESM.zip › Supplementary_images of the spectral observations/A.jpeg]

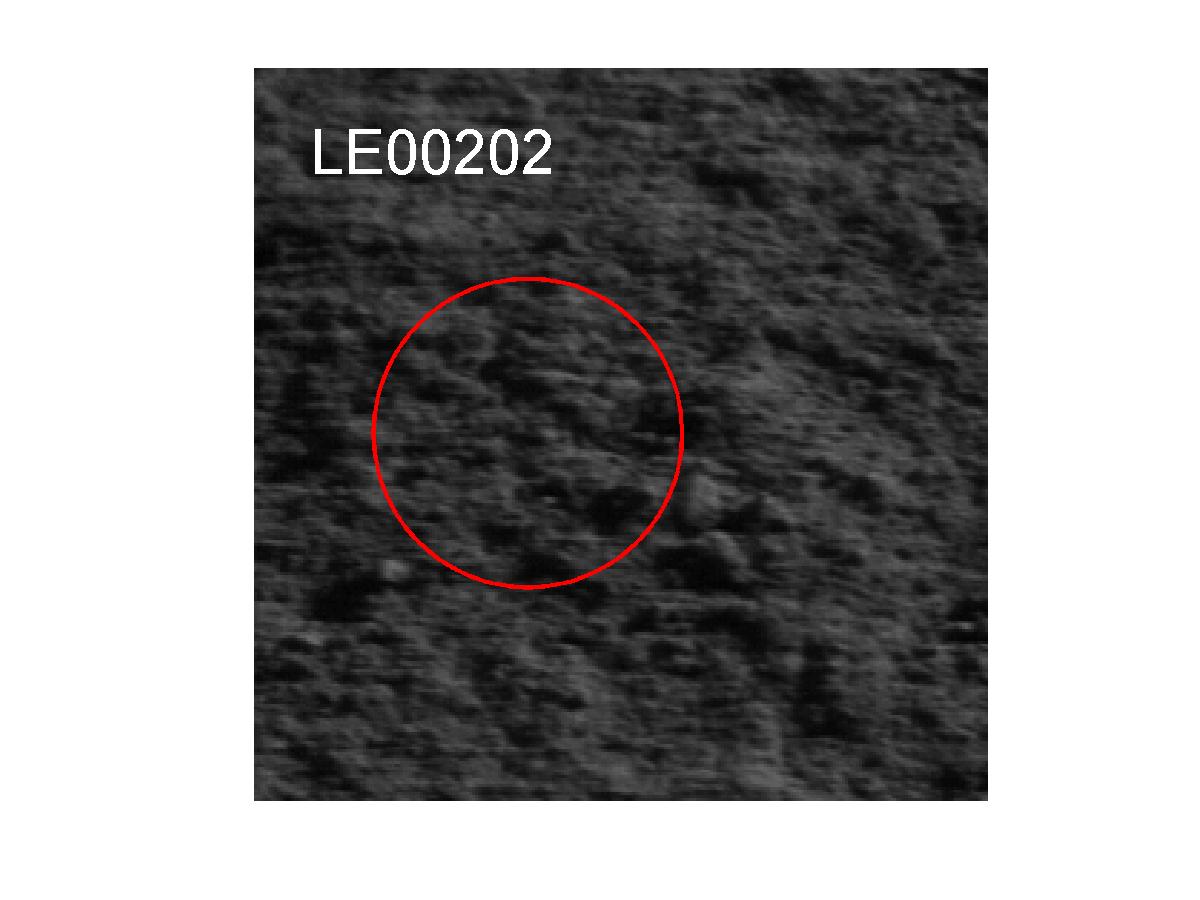

Supplement: Supplementary file 1 — Supplementary Information 1. [file 41598_2021_93694_MOESM1_ESM.zip › Supplementary_images of the spectral observations/LE00202.jpeg]

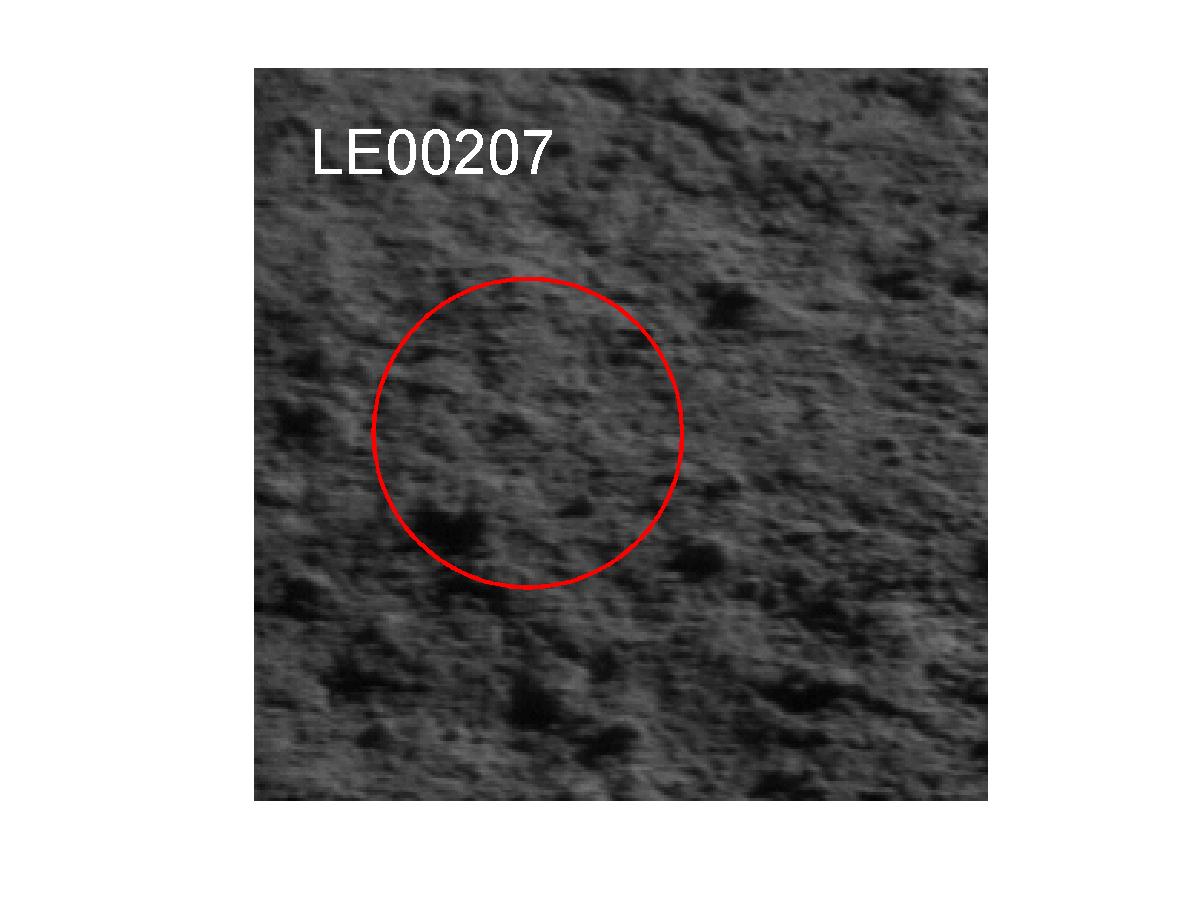

Supplement: Supplementary file 1 — Supplementary Information 1. [file 41598_2021_93694_MOESM1_ESM.zip › Supplementary_images of the spectral observations/LE00207.jpeg]

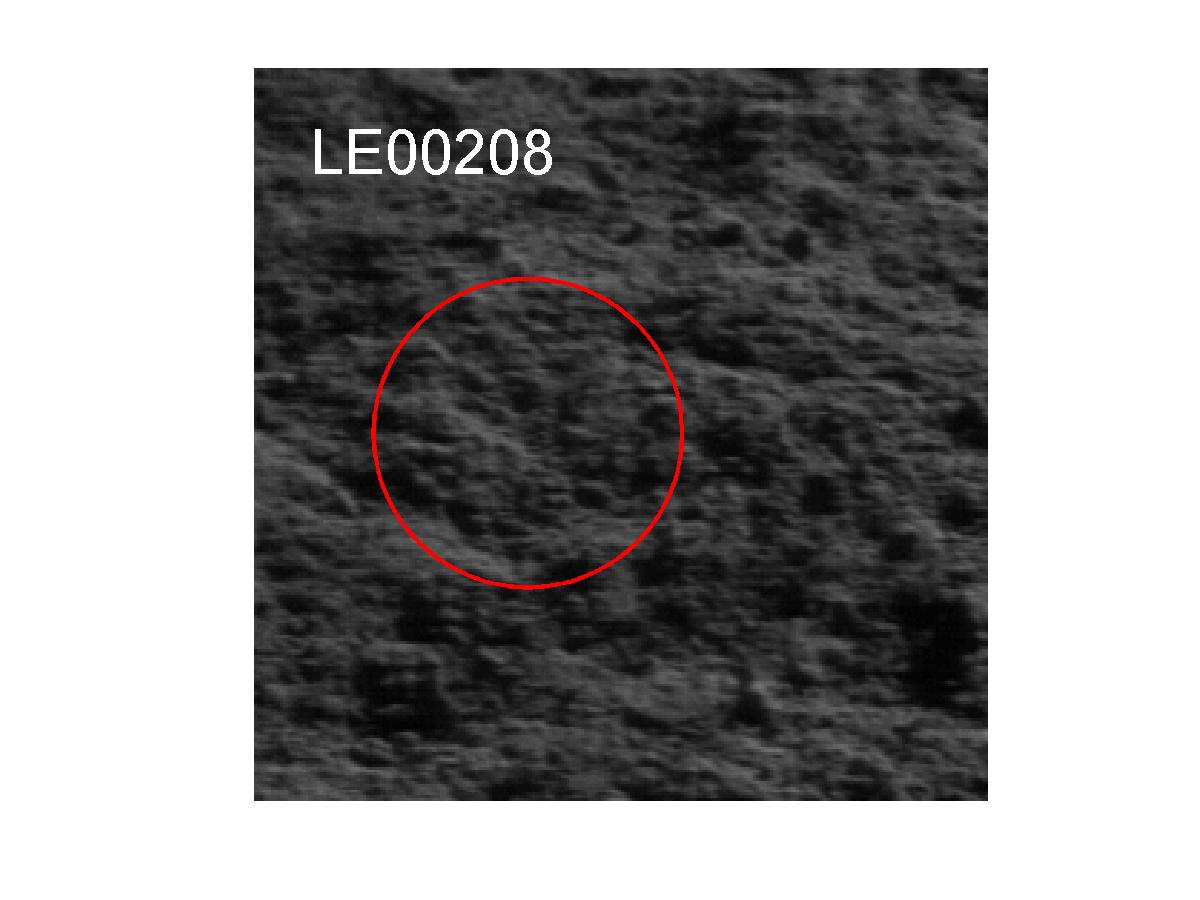

Supplement: Supplementary file 1 — Supplementary Information 1. [file 41598_2021_93694_MOESM1_ESM.zip › Supplementary_images of the spectral observations/LE00208.jpeg]

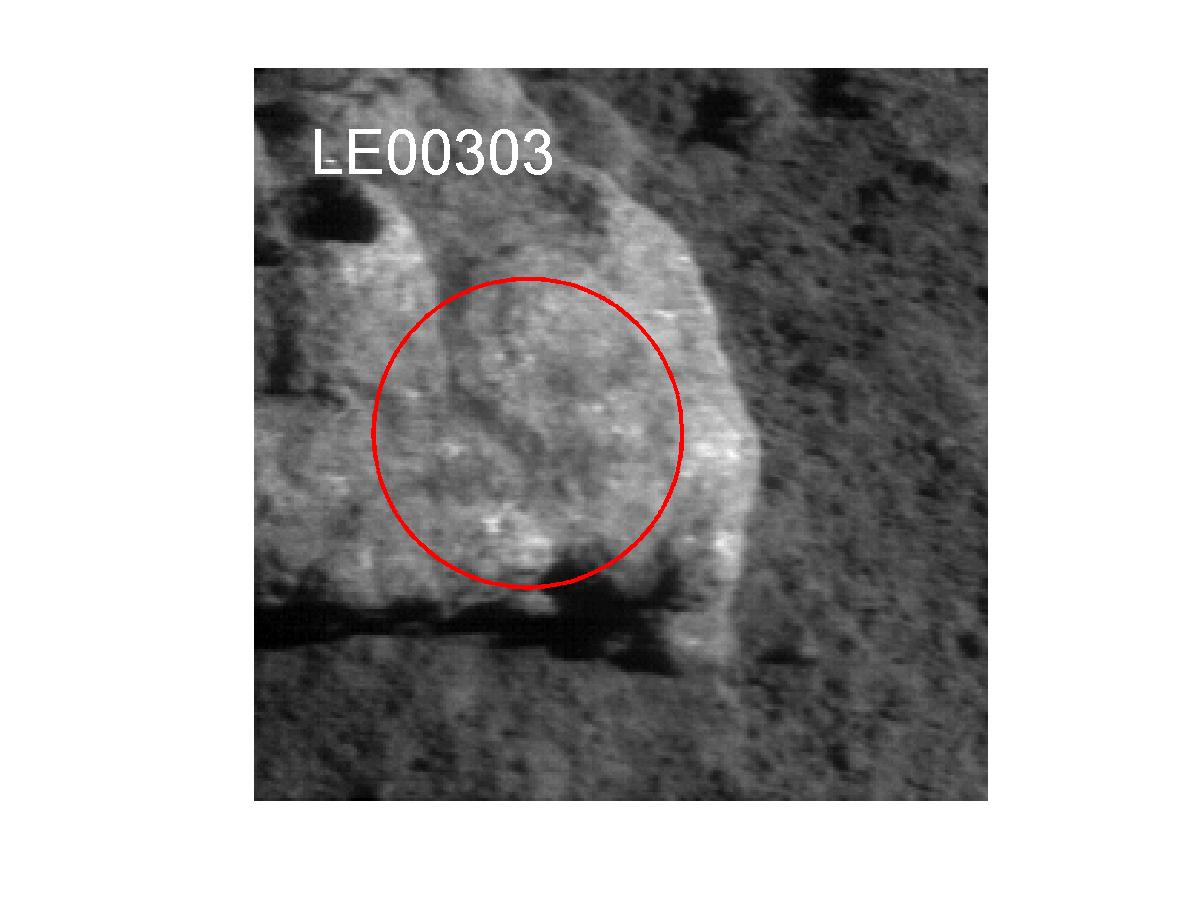

Supplement: Supplementary file 1 — Supplementary Information 1. [file 41598_2021_93694_MOESM1_ESM.zip › Supplementary_images of the spectral observations/LE00303.jpeg]

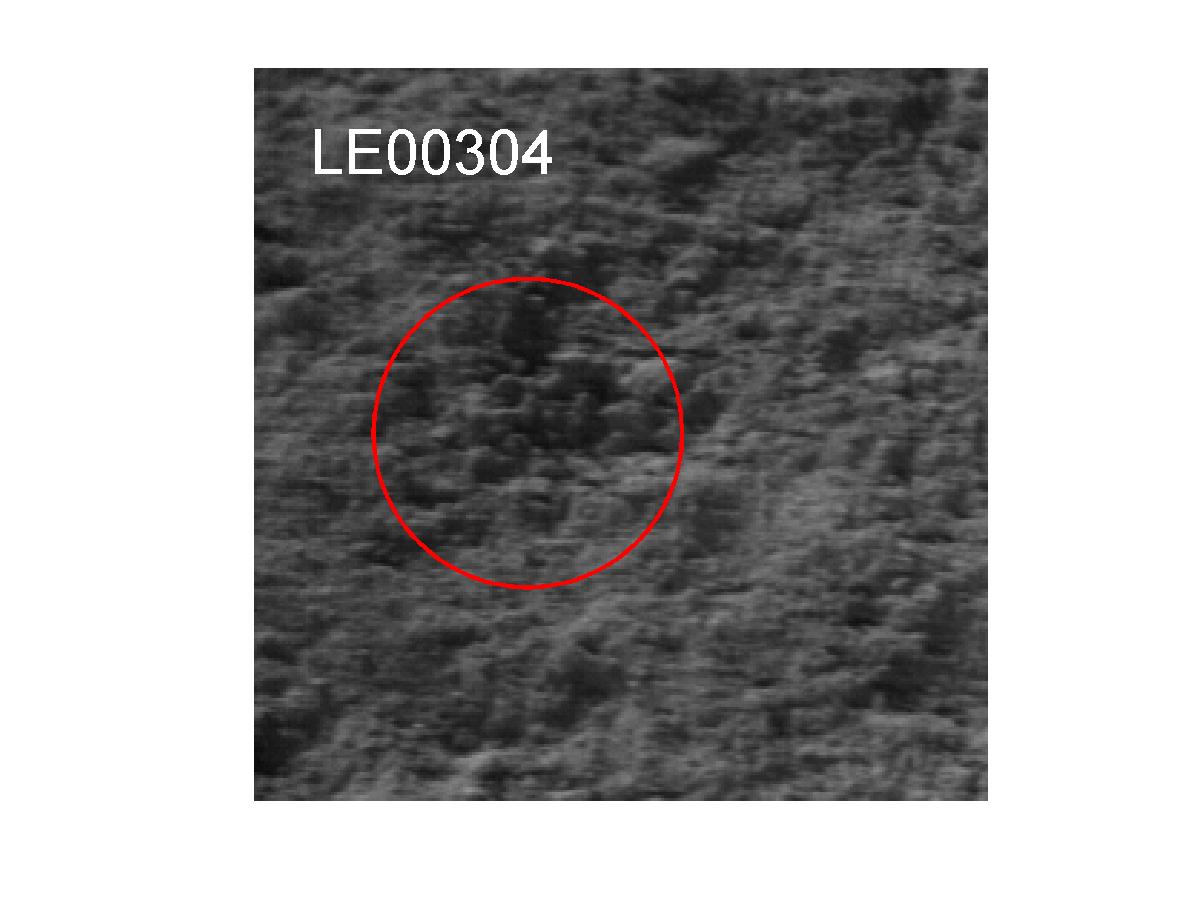

Supplement: Supplementary file 1 — Supplementary Information 1. [file 41598_2021_93694_MOESM1_ESM.zip › Supplementary_images of the spectral observations/LE00304.jpeg]

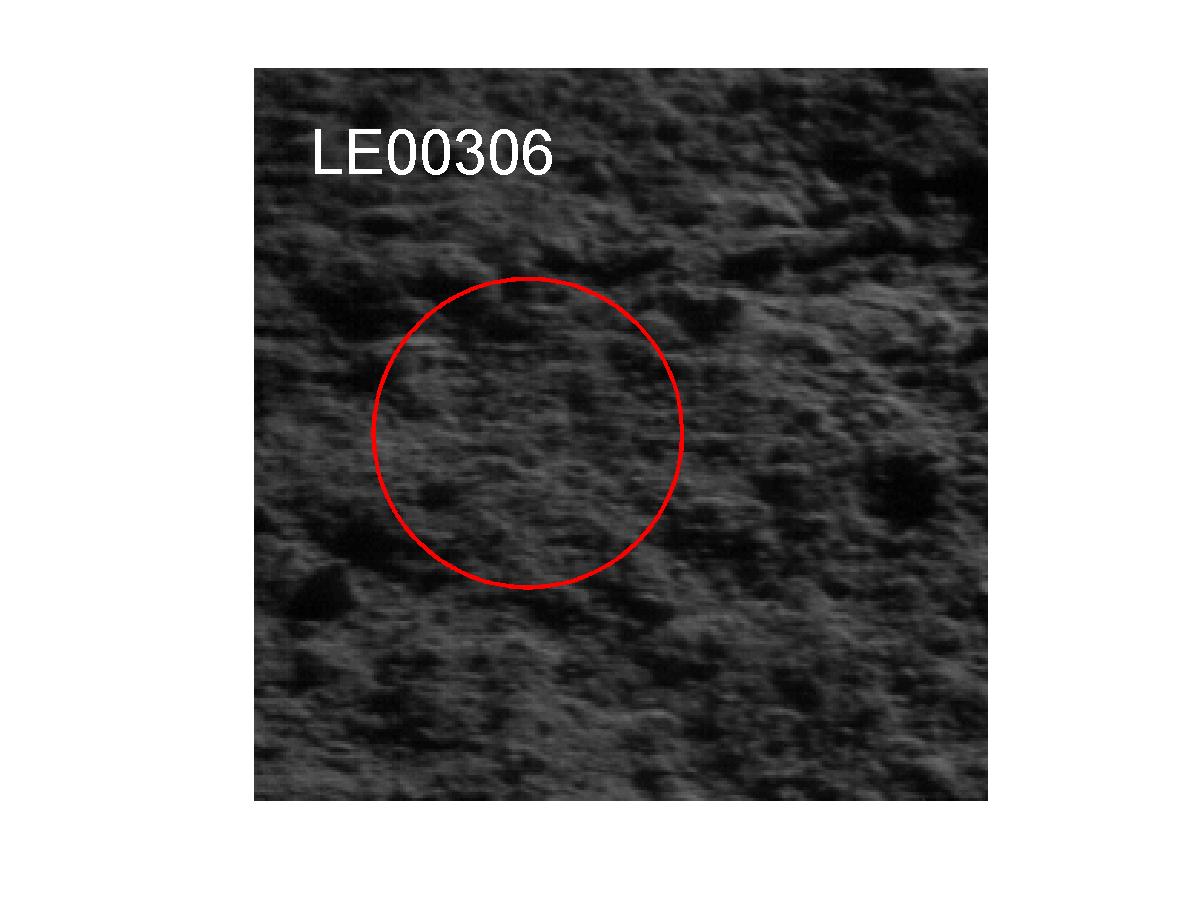

Supplement: Supplementary file 1 — Supplementary Information 1. [file 41598_2021_93694_MOESM1_ESM.zip › Supplementary_images of the spectral observations/LE00306.jpeg]

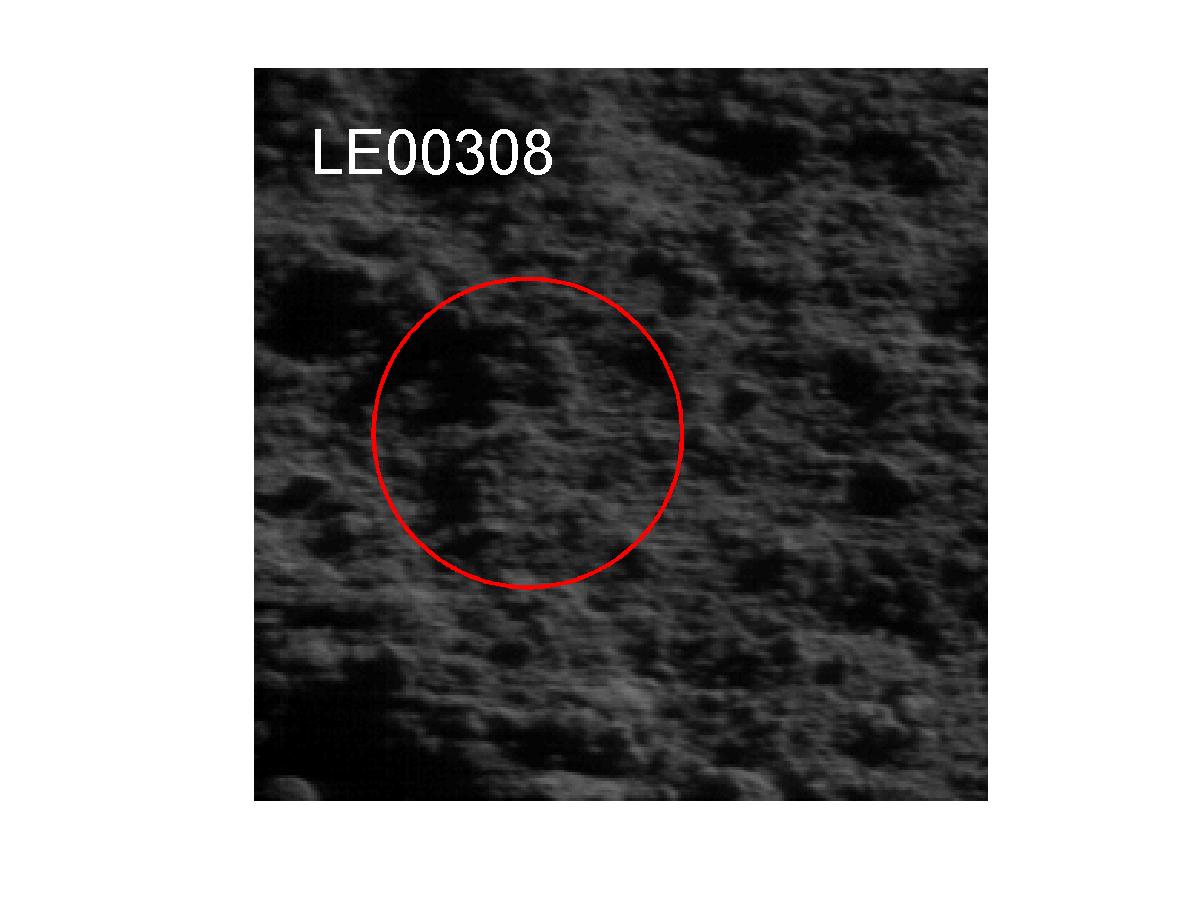

Supplement: Supplementary file 1 — Supplementary Information 1. [file 41598_2021_93694_MOESM1_ESM.zip › Supplementary_images of the spectral observations/LE00308.jpeg]

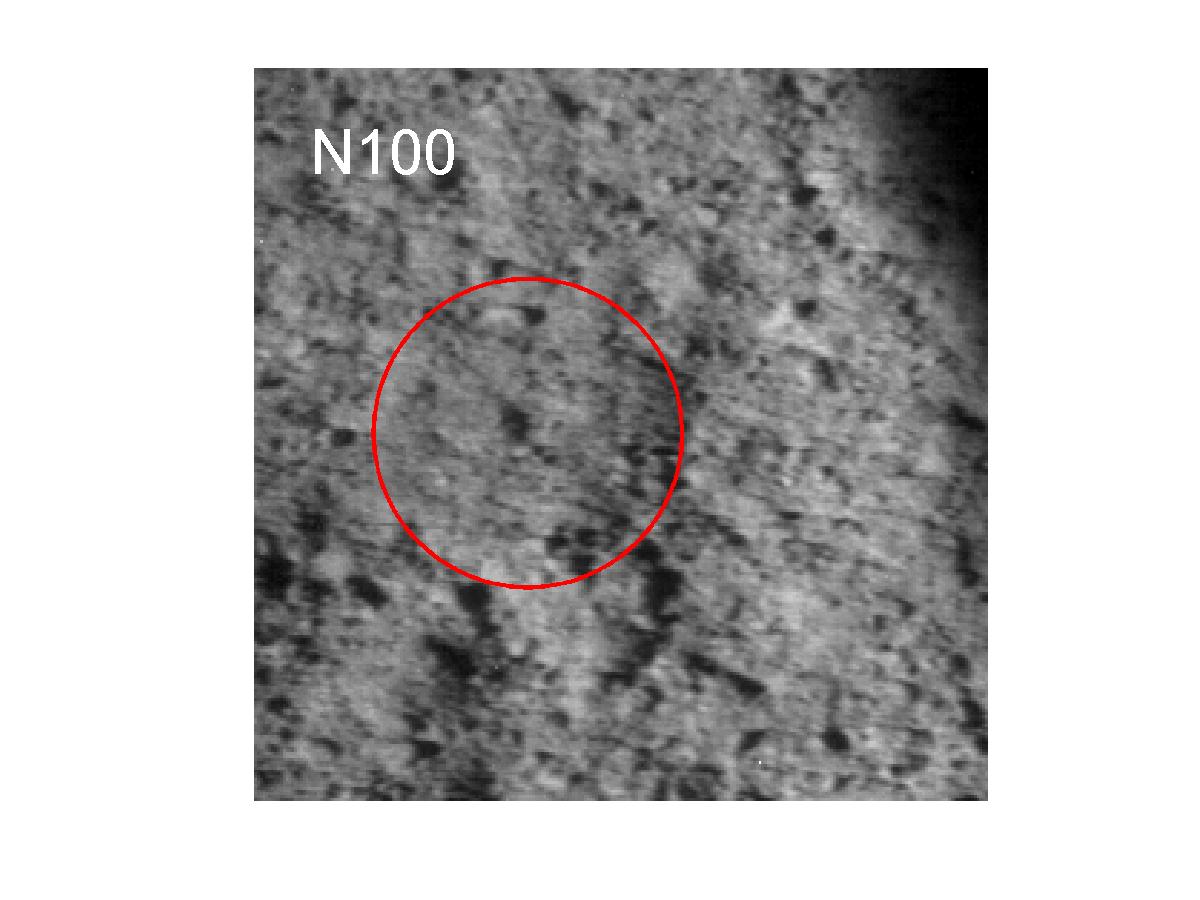

Supplement: Supplementary file 1 — Supplementary Information 1. [file 41598_2021_93694_MOESM1_ESM.zip › Supplementary_images of the spectral observations/N100.jpeg]

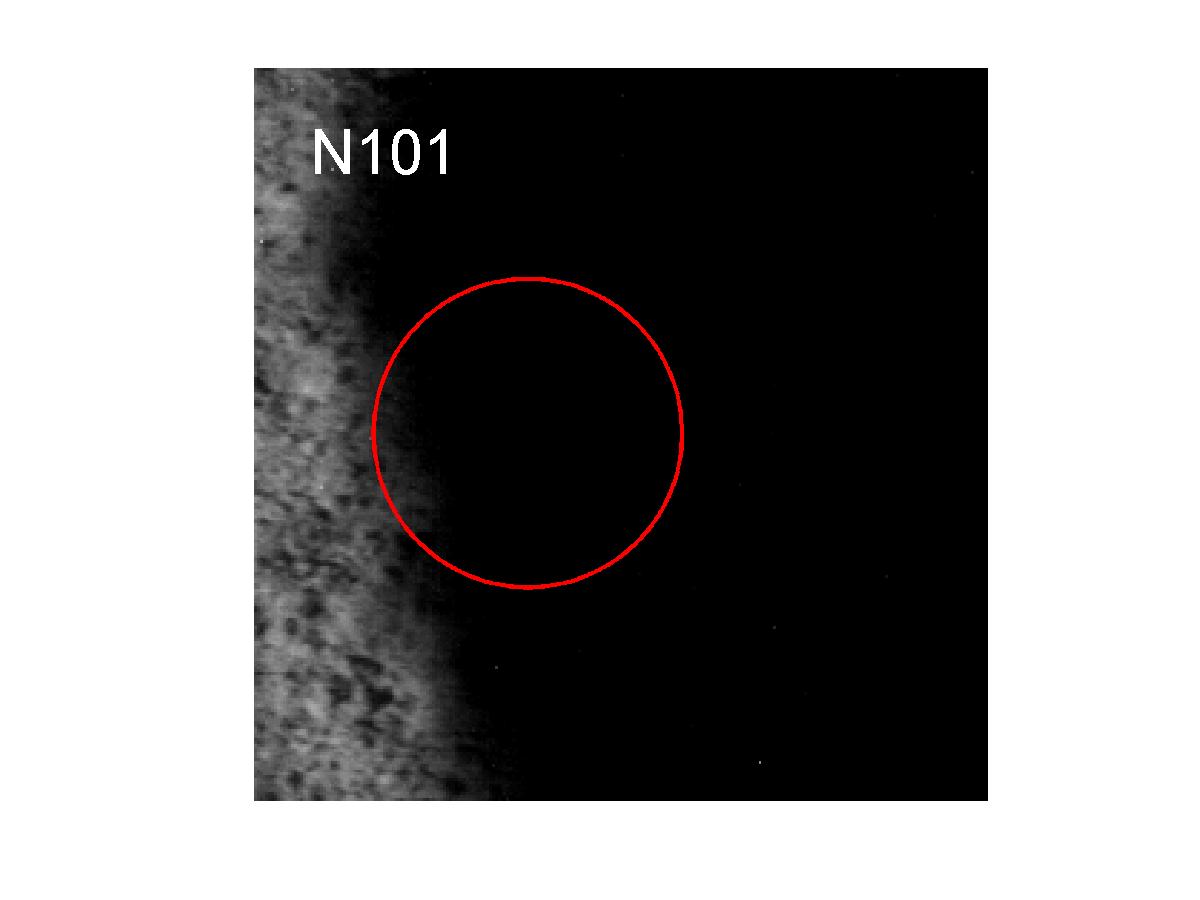

Supplement: Supplementary file 1 — Supplementary Information 1. [file 41598_2021_93694_MOESM1_ESM.zip › Supplementary_images of the spectral observations/N101.jpeg]

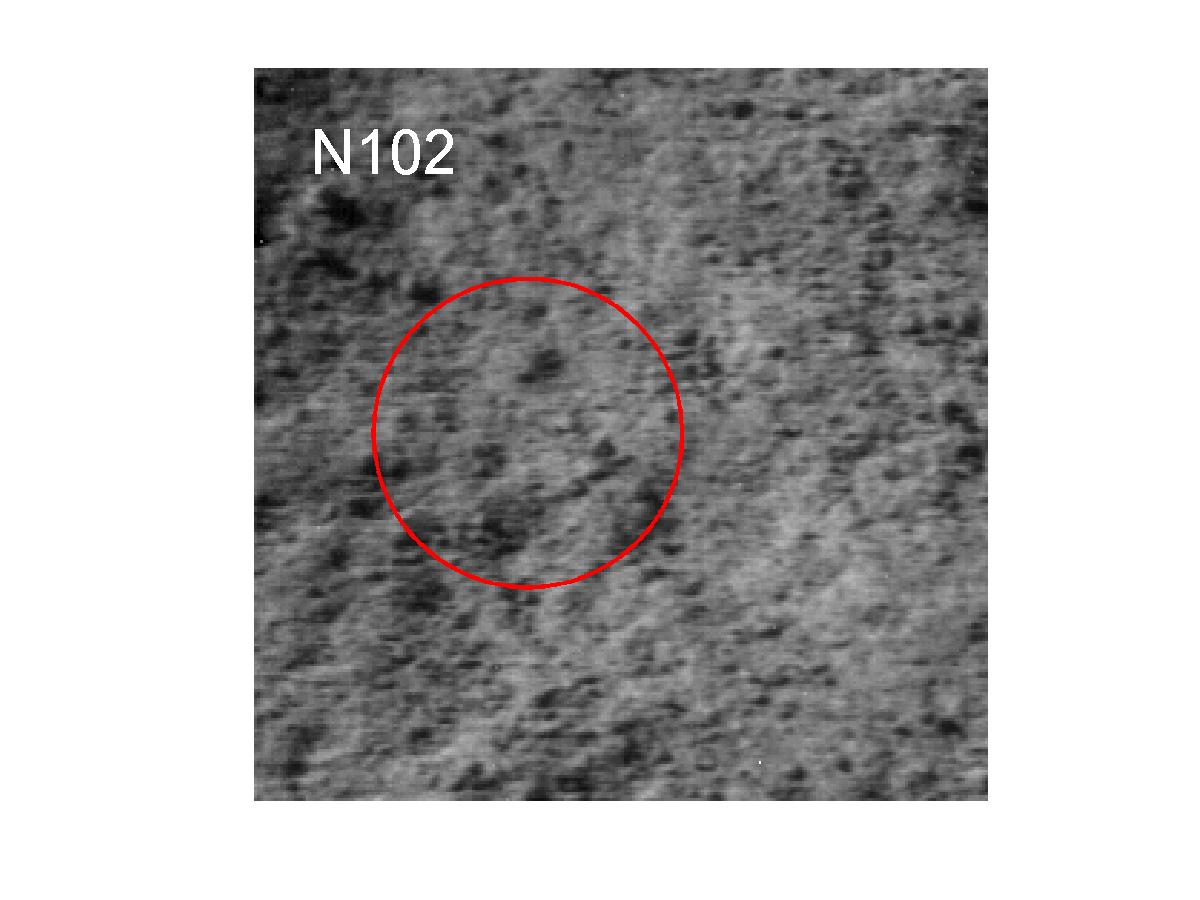

Supplement: Supplementary file 1 — Supplementary Information 1. [file 41598_2021_93694_MOESM1_ESM.zip › Supplementary_images of the spectral observations/N102.jpeg]

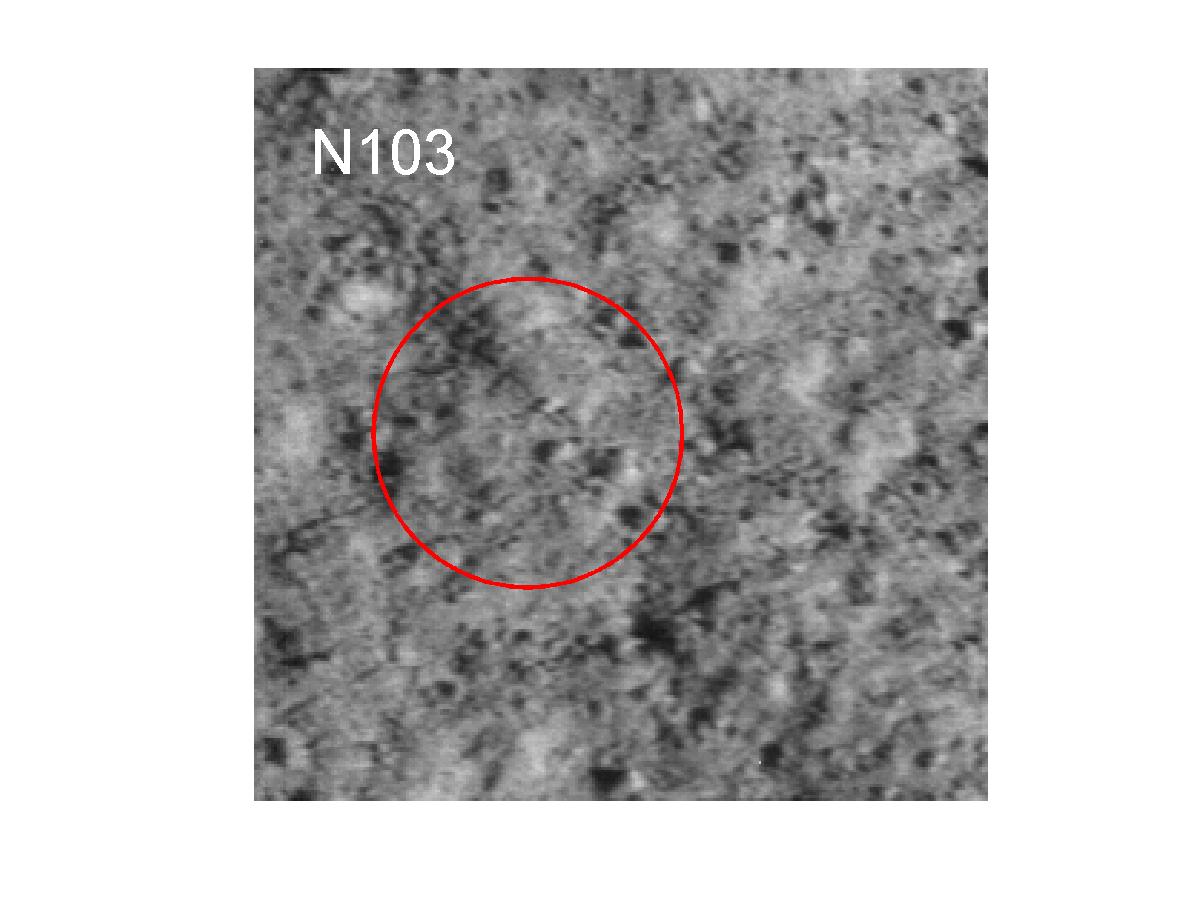

Supplement: Supplementary file 1 — Supplementary Information 1. [file 41598_2021_93694_MOESM1_ESM.zip › Supplementary_images of the spectral observations/N103.jpeg]

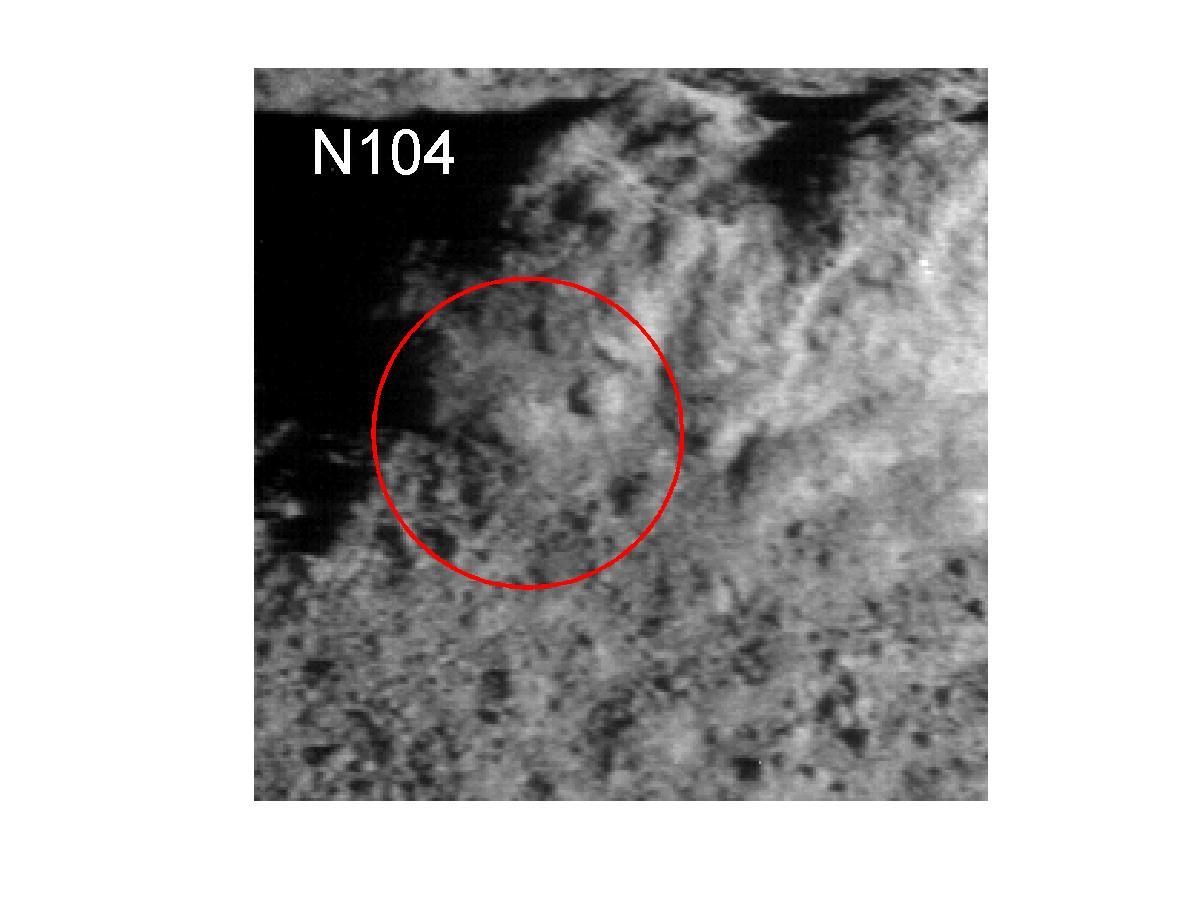

Supplement: Supplementary file 1 — Supplementary Information 1. [file 41598_2021_93694_MOESM1_ESM.zip › Supplementary_images of the spectral observations/N104.jpeg]

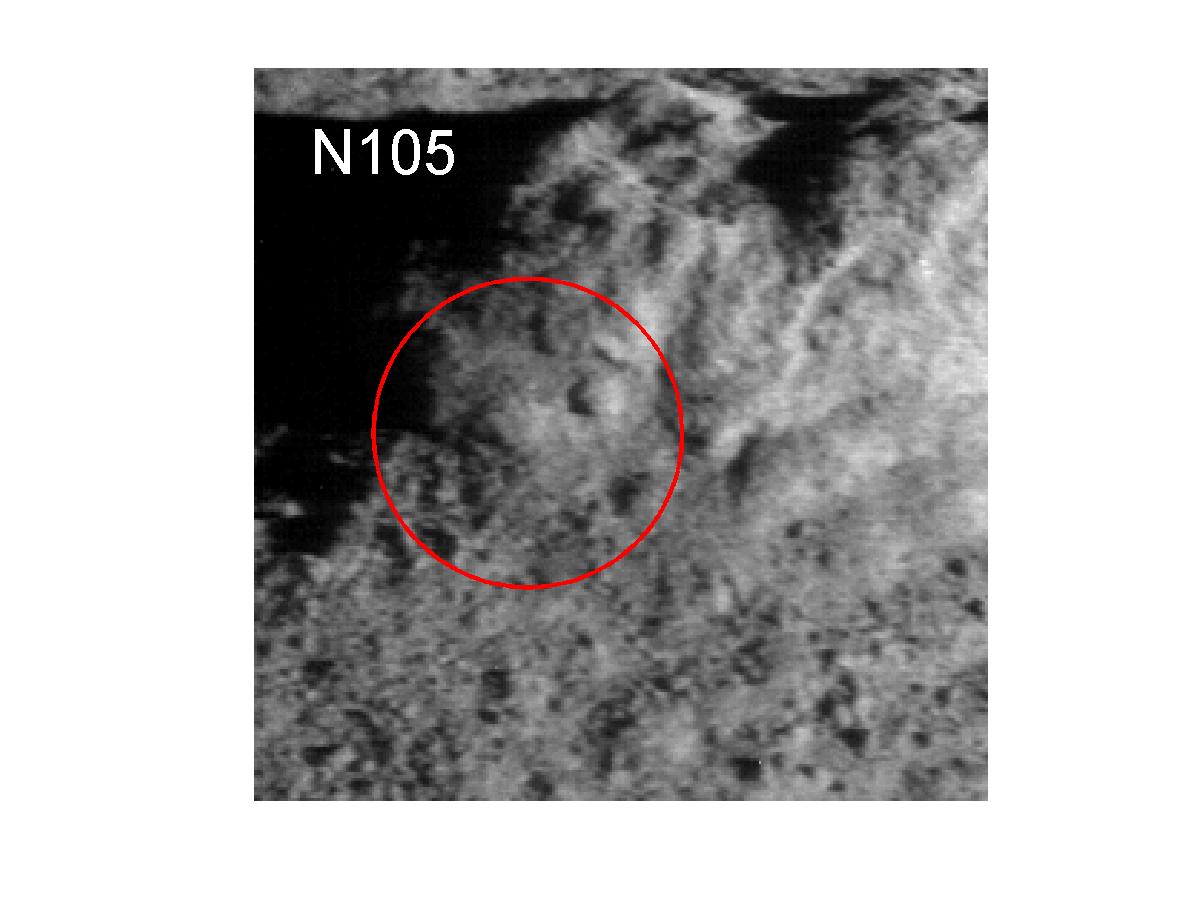

Supplement: Supplementary file 1 — Supplementary Information 1. [file 41598_2021_93694_MOESM1_ESM.zip › Supplementary_images of the spectral observations/N105.jpeg]

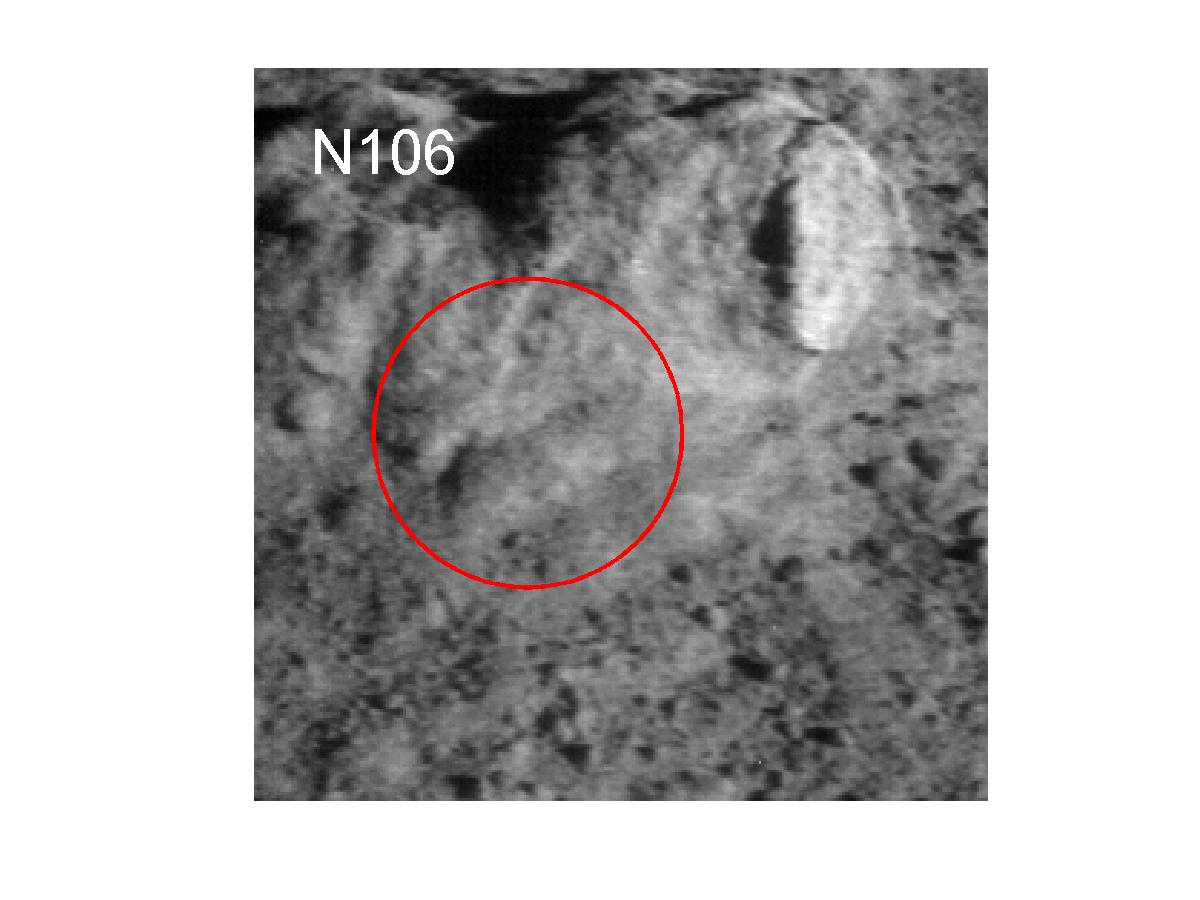

Supplement: Supplementary file 1 — Supplementary Information 1. [file 41598_2021_93694_MOESM1_ESM.zip › Supplementary_images of the spectral observations/N106.jpeg]

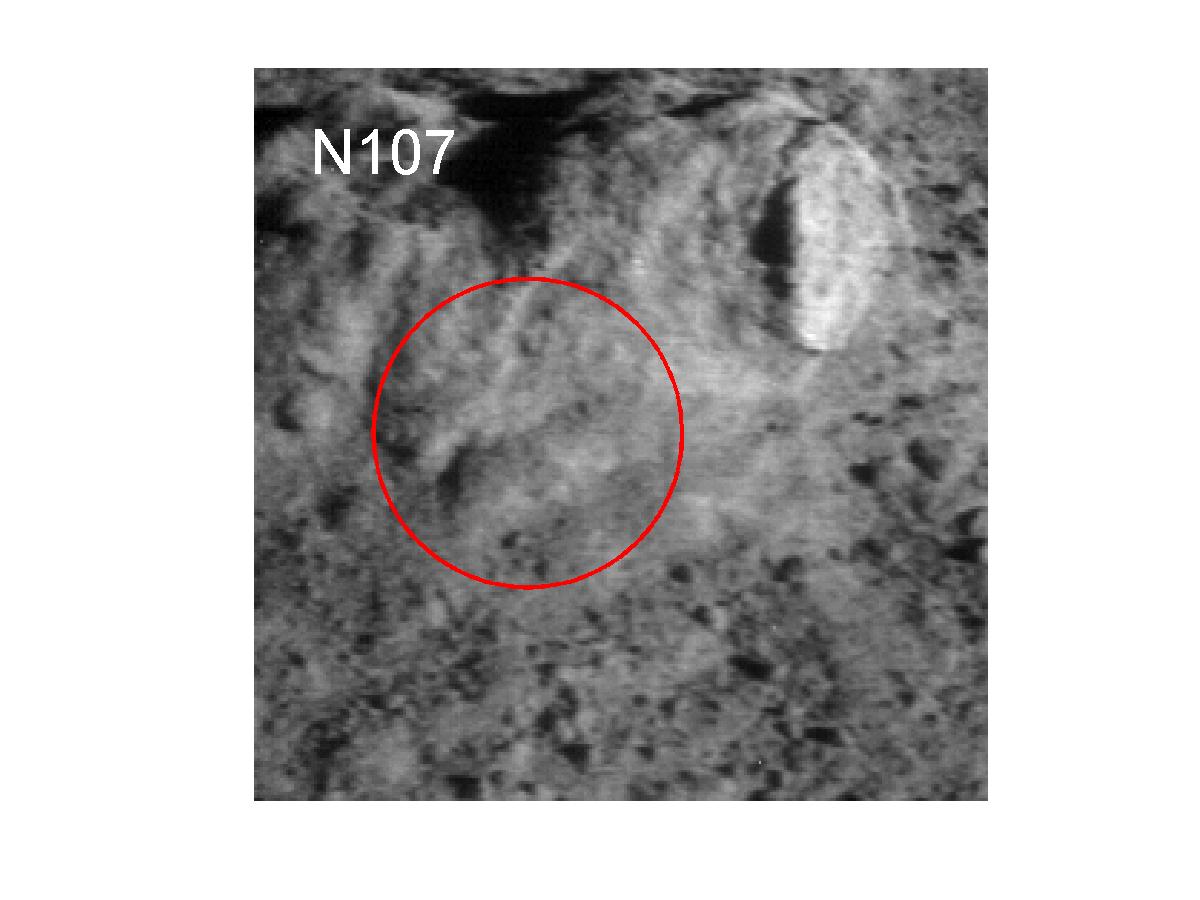

Supplement: Supplementary file 1 — Supplementary Information 1. [file 41598_2021_93694_MOESM1_ESM.zip › Supplementary_images of the spectral observations/N107.jpeg]

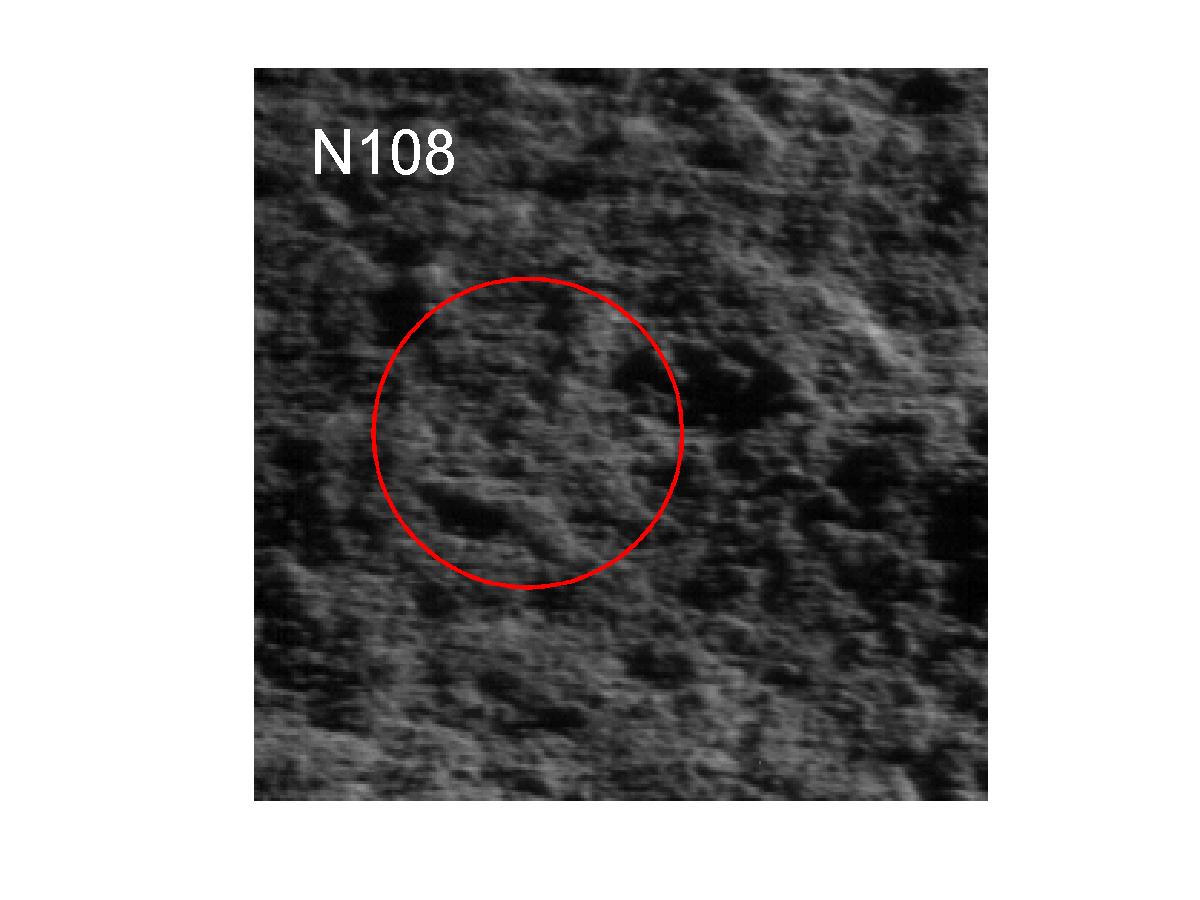

Supplement: Supplementary file 1 — Supplementary Information 1. [file 41598_2021_93694_MOESM1_ESM.zip › Supplementary_images of the spectral observations/N108.jpeg]

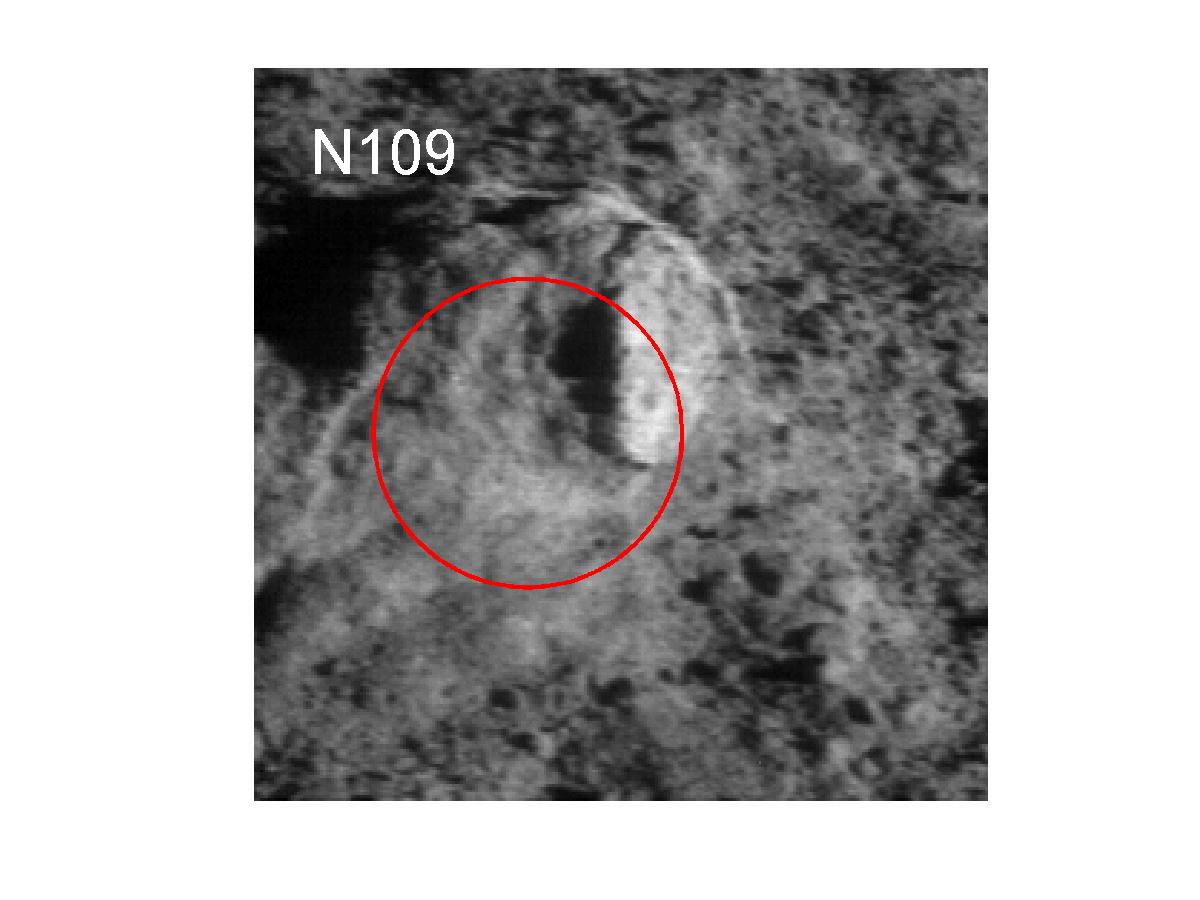

Supplement: Supplementary file 1 — Supplementary Information 1. [file 41598_2021_93694_MOESM1_ESM.zip › Supplementary_images of the spectral observations/N109.jpeg]

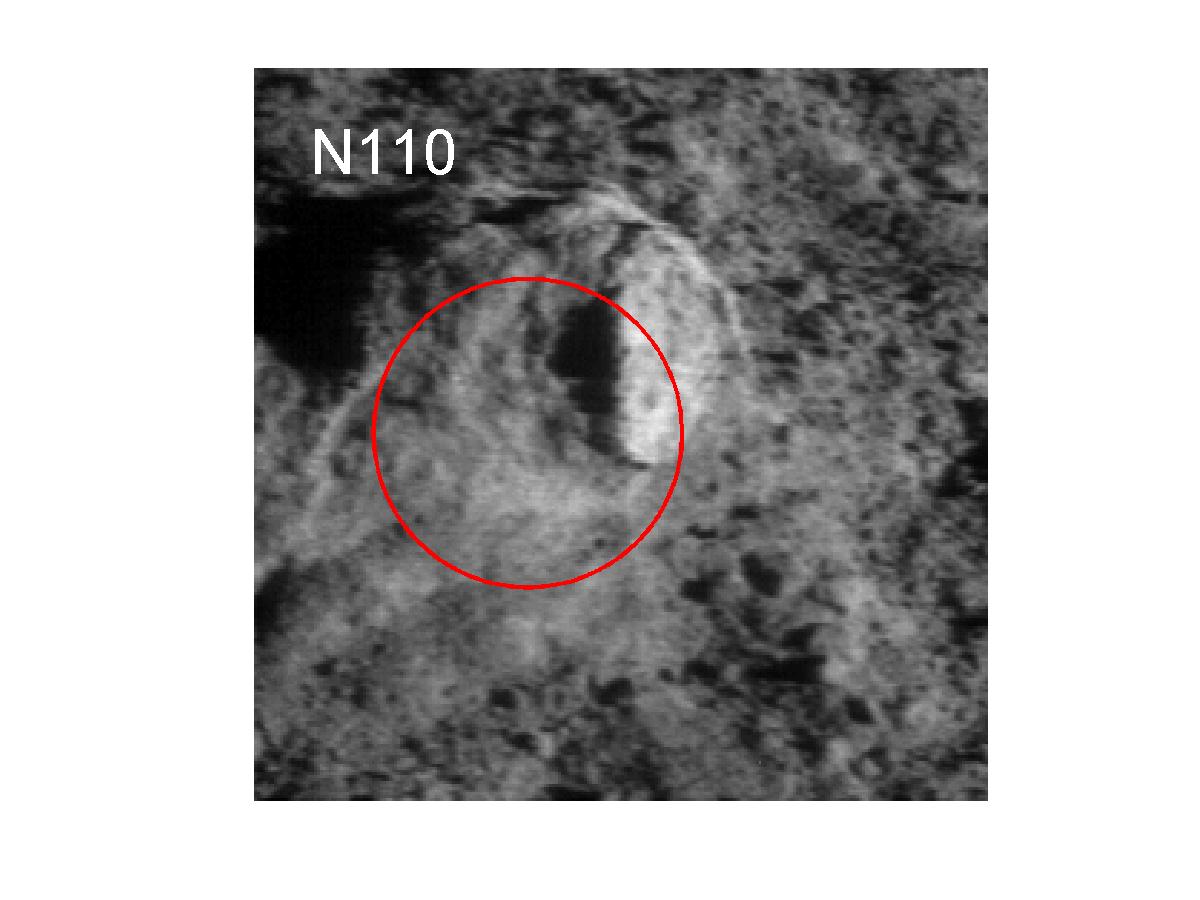

Supplement: Supplementary file 1 — Supplementary Information 1. [file 41598_2021_93694_MOESM1_ESM.zip › Supplementary_images of the spectral observations/N110.jpeg]

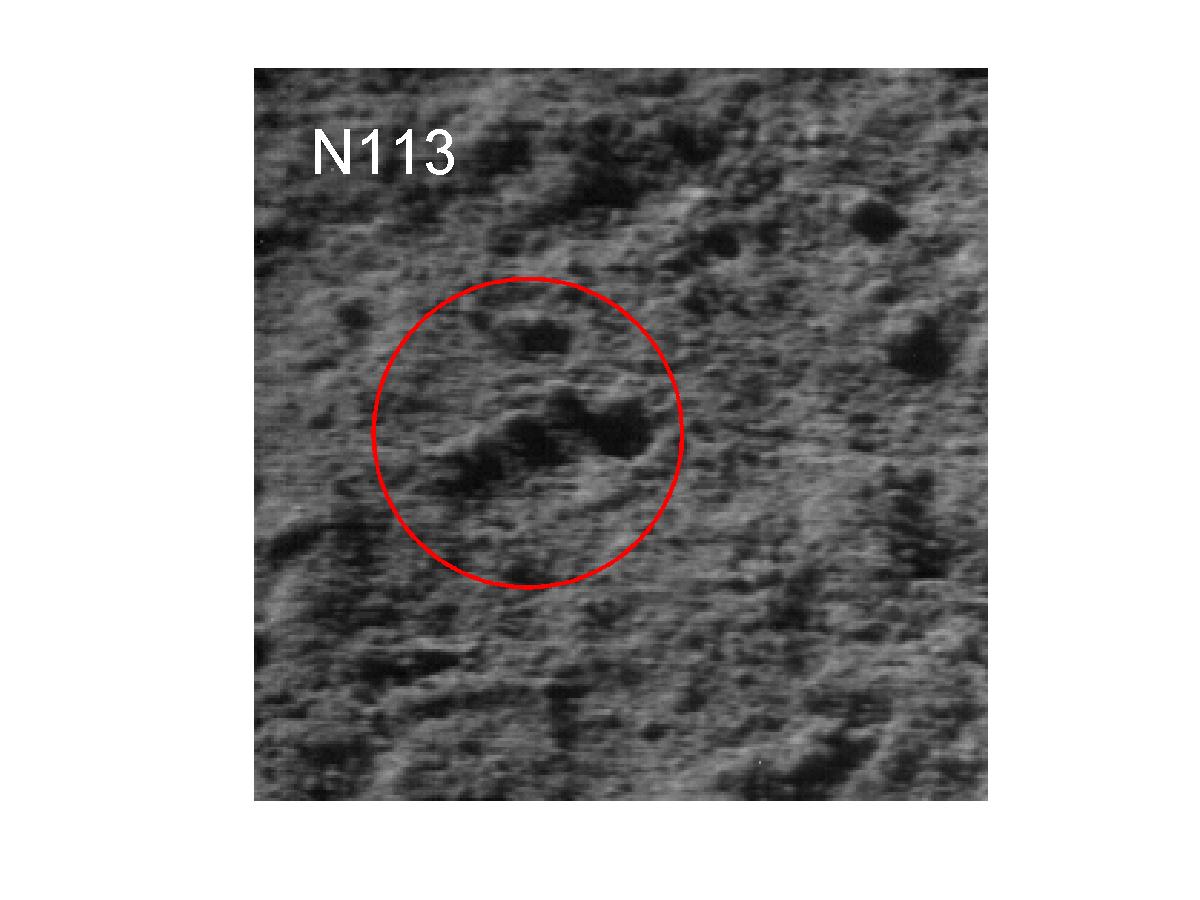

Supplement: Supplementary file 1 — Supplementary Information 1. [file 41598_2021_93694_MOESM1_ESM.zip › Supplementary_images of the spectral observations/N113.jpeg]

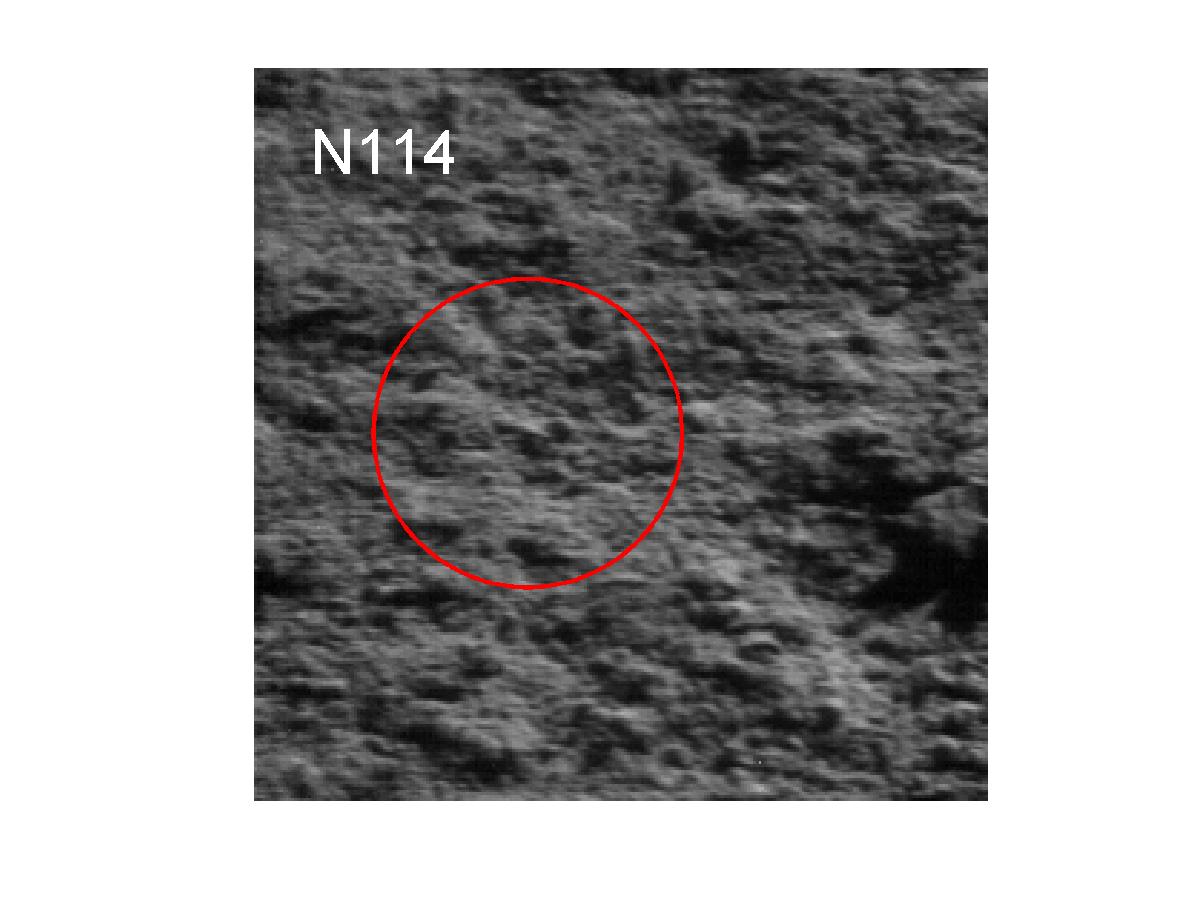

Supplement: Supplementary file 1 — Supplementary Information 1. [file 41598_2021_93694_MOESM1_ESM.zip › Supplementary_images of the spectral observations/N114.jpeg]

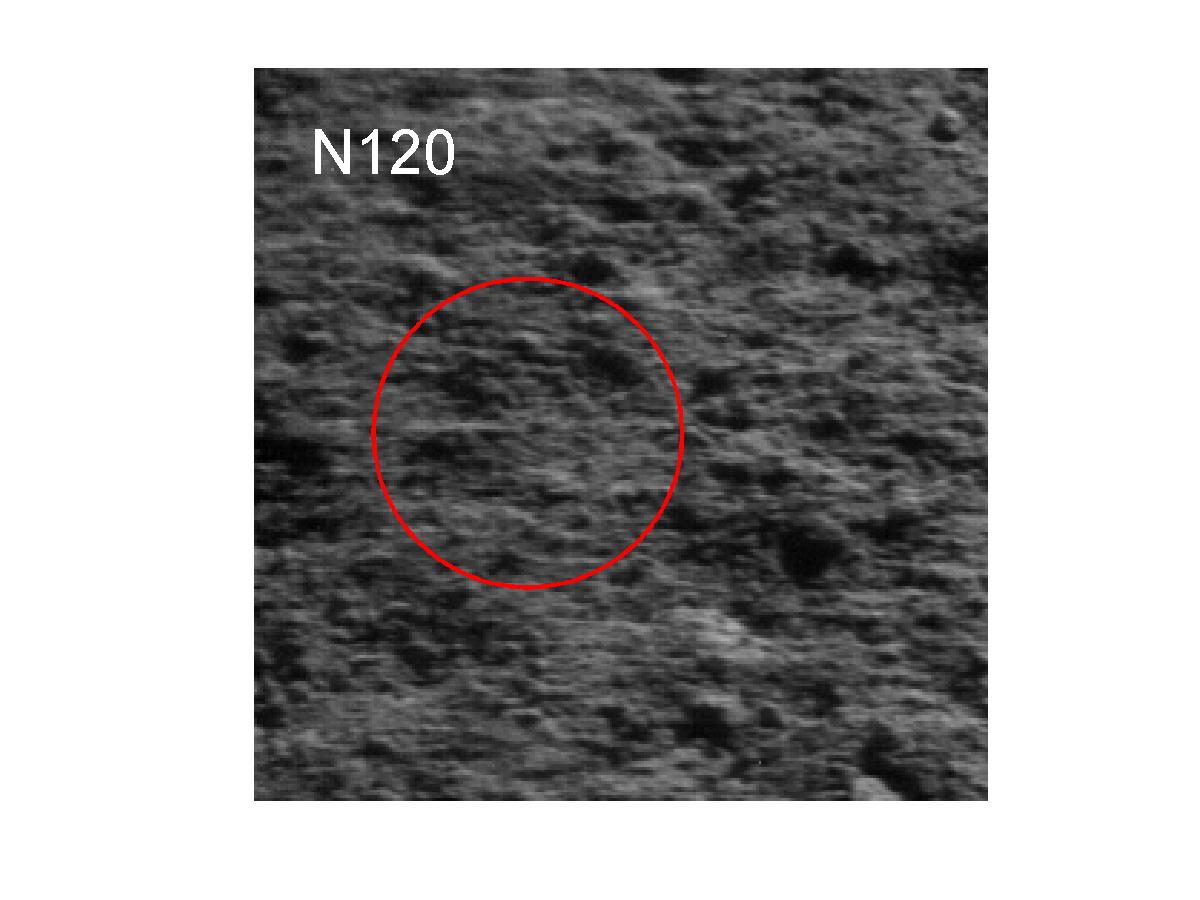

Supplement: Supplementary file 1 — Supplementary Information 1. [file 41598_2021_93694_MOESM1_ESM.zip › Supplementary_images of the spectral observations/N120.jpeg]

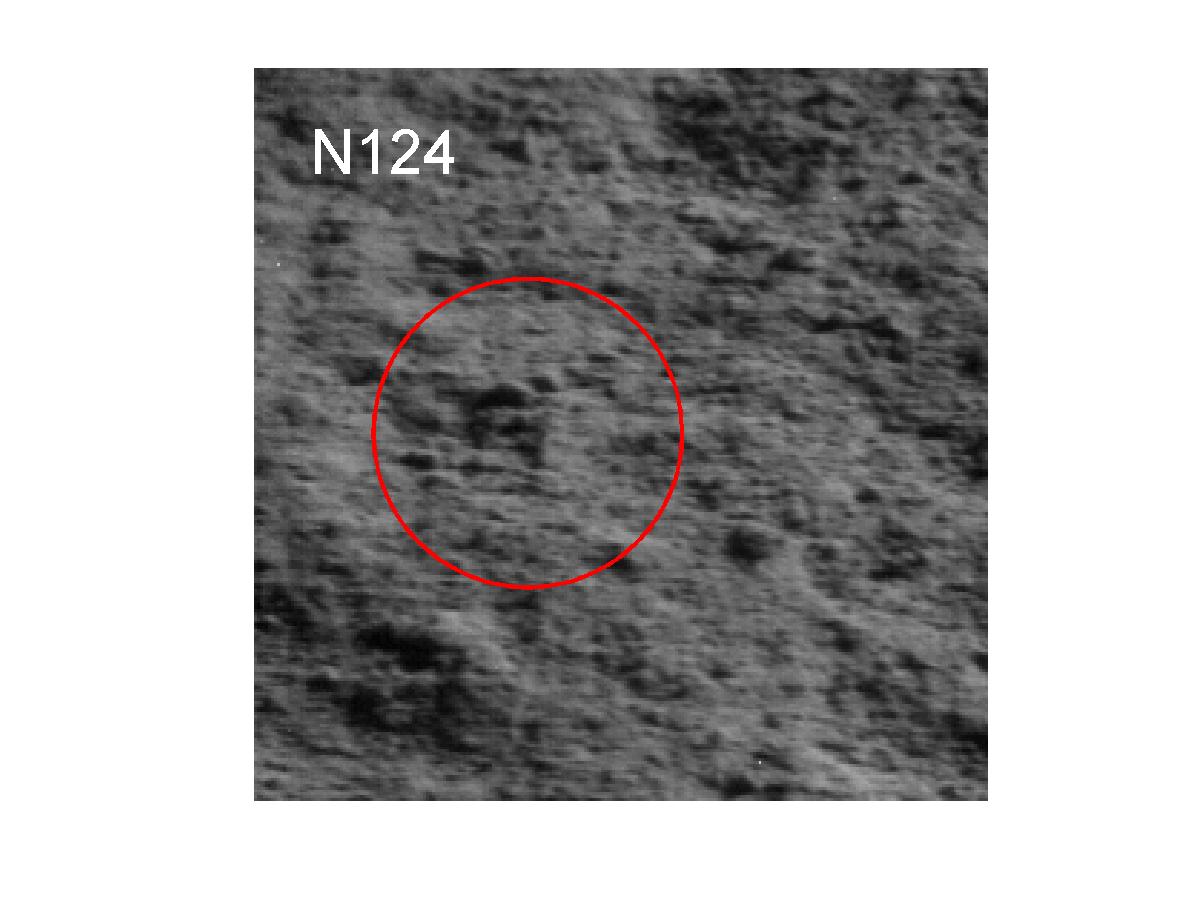

Supplement: Supplementary file 1 — Supplementary Information 1. [file 41598_2021_93694_MOESM1_ESM.zip › Supplementary_images of the spectral observations/N124.jpeg]

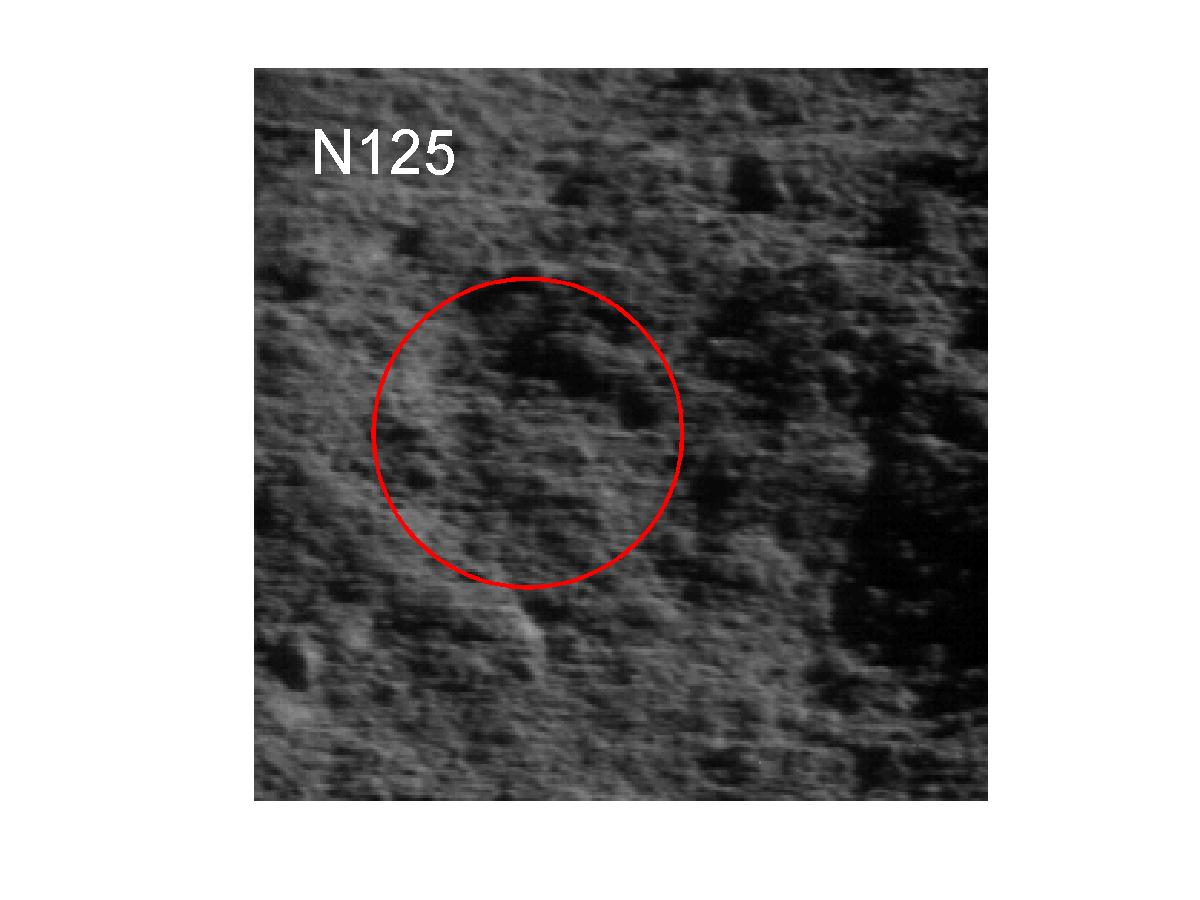

Supplement: Supplementary file 1 — Supplementary Information 1. [file 41598_2021_93694_MOESM1_ESM.zip › Supplementary_images of the spectral observations/N125.jpeg]

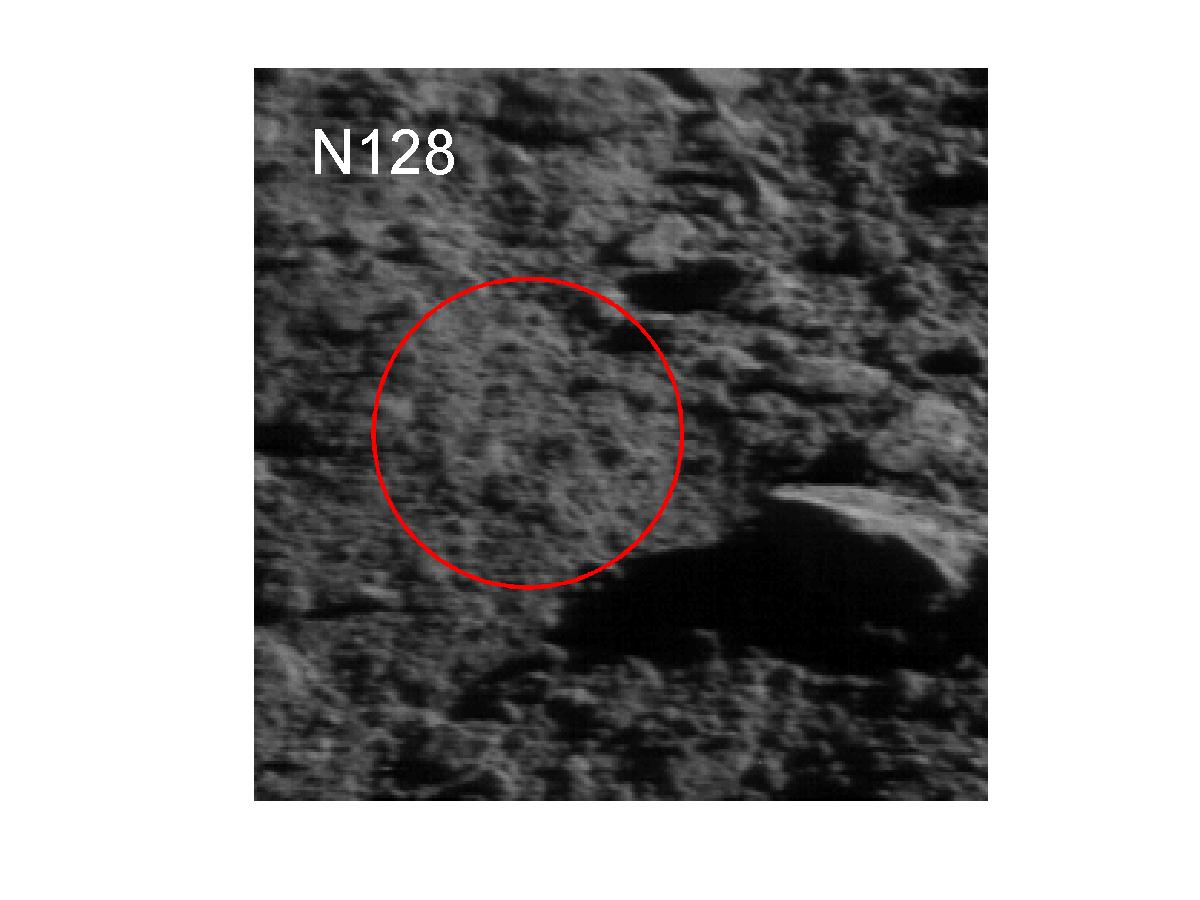

Supplement: Supplementary file 1 — Supplementary Information 1. [file 41598_2021_93694_MOESM1_ESM.zip › Supplementary_images of the spectral observations/N128.jpeg]

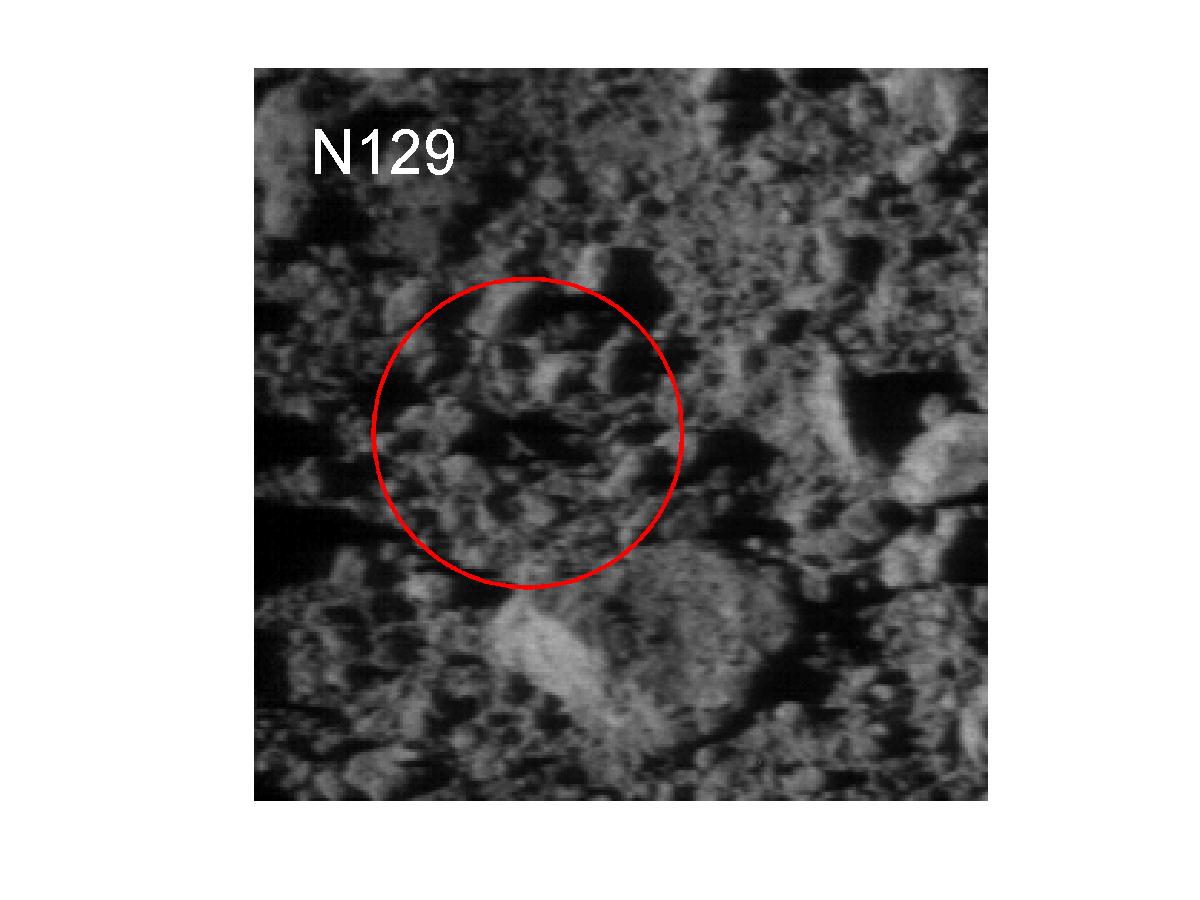

Supplement: Supplementary file 1 — Supplementary Information 1. [file 41598_2021_93694_MOESM1_ESM.zip › Supplementary_images of the spectral observations/N129.jpeg]

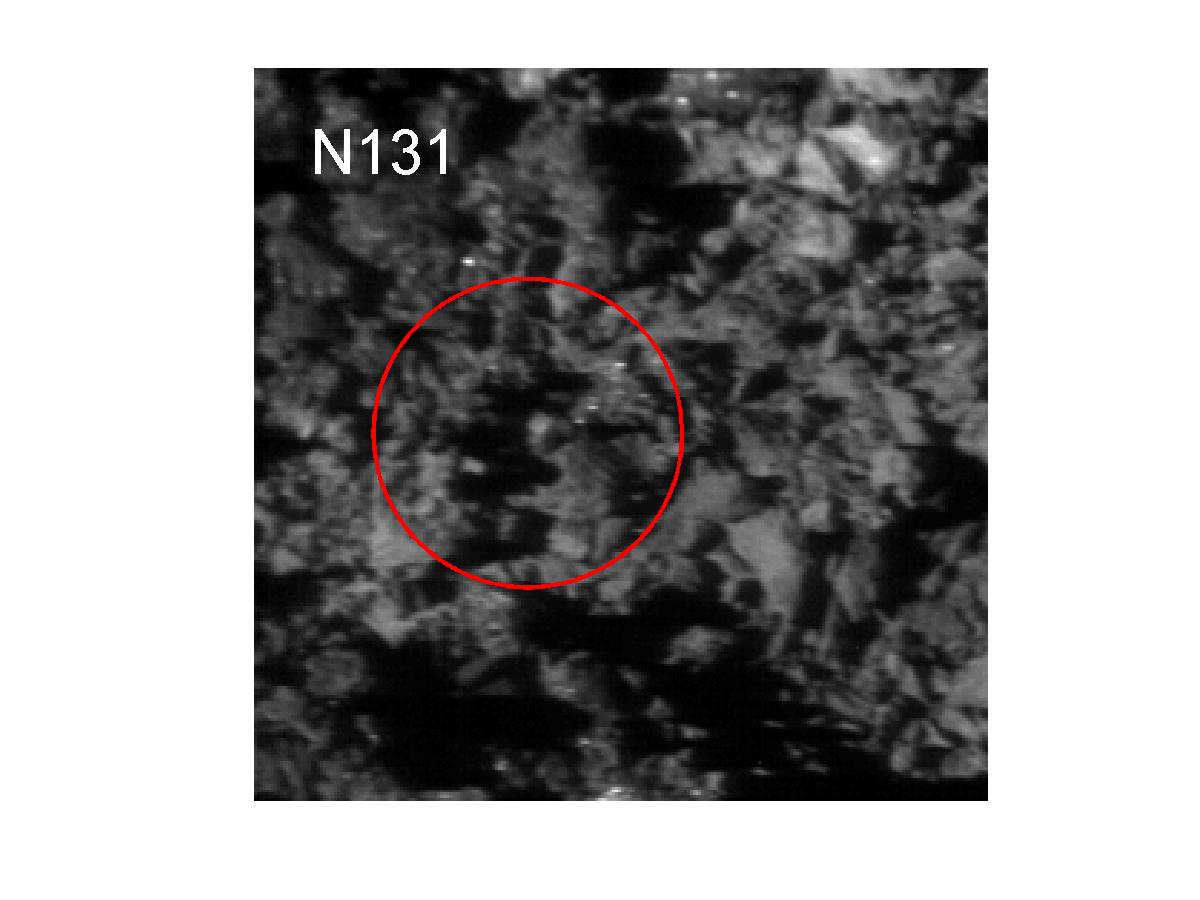

Supplement: Supplementary file 1 — Supplementary Information 1. [file 41598_2021_93694_MOESM1_ESM.zip › Supplementary_images of the spectral observations/N131.jpeg]

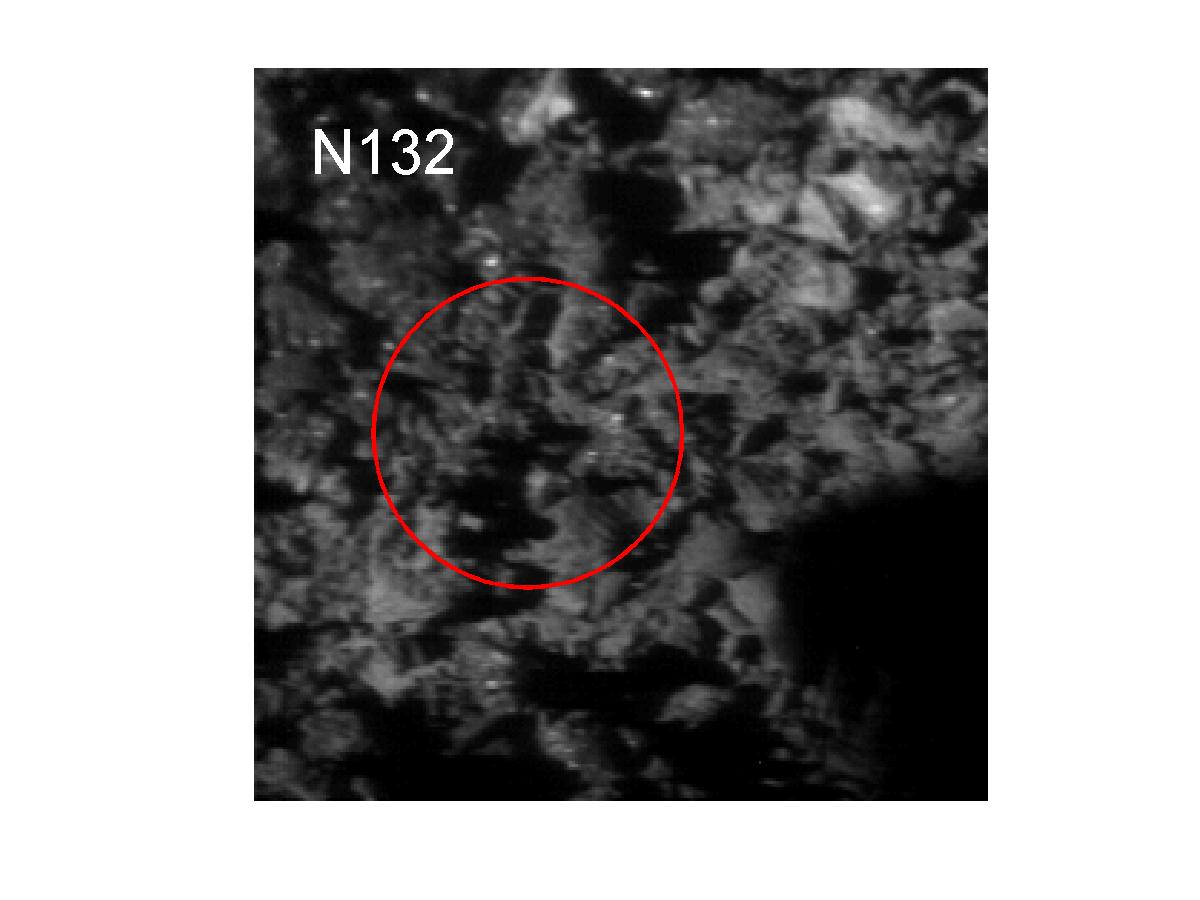

Supplement: Supplementary file 1 — Supplementary Information 1. [file 41598_2021_93694_MOESM1_ESM.zip › Supplementary_images of the spectral observations/N132.jpeg]

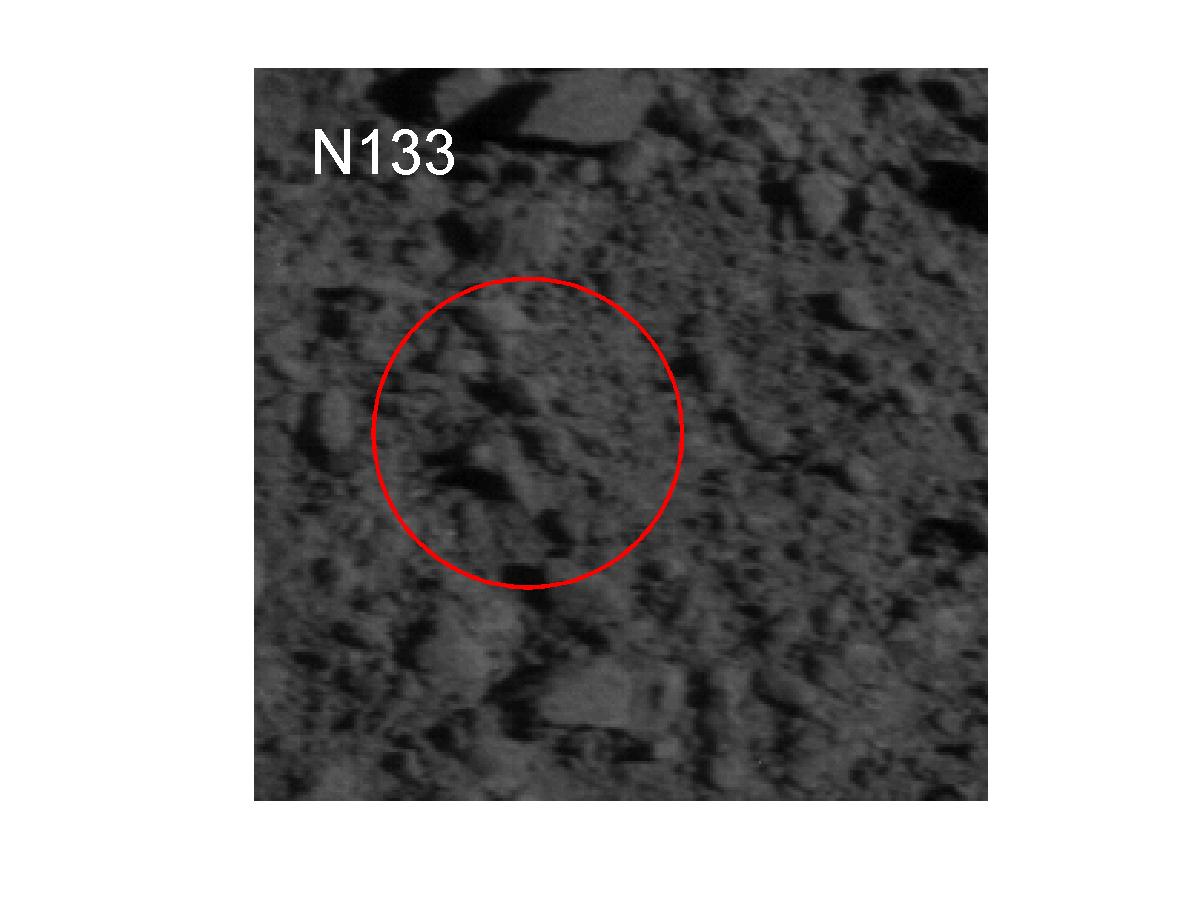

Supplement: Supplementary file 1 — Supplementary Information 1. [file 41598_2021_93694_MOESM1_ESM.zip › Supplementary_images of the spectral observations/N133.jpeg]

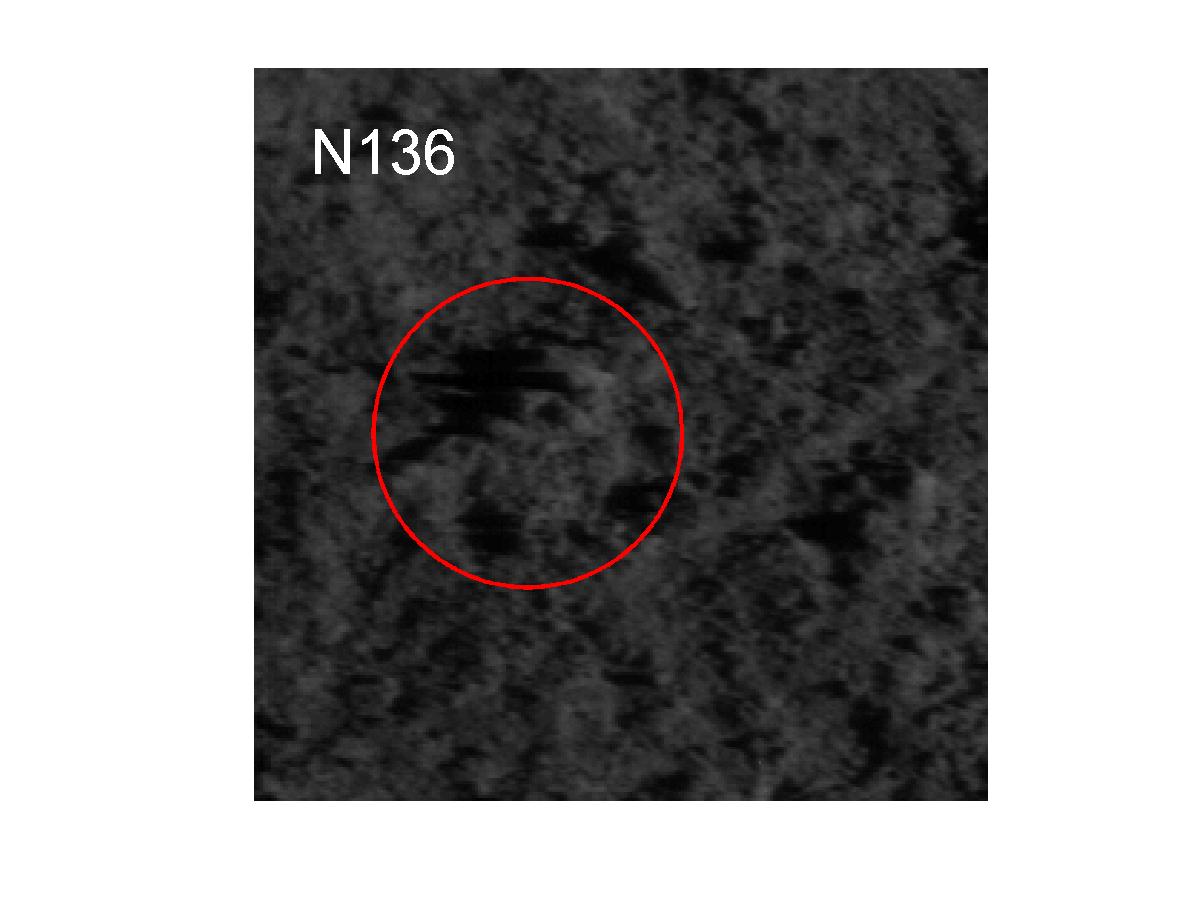

Supplement: Supplementary file 1 — Supplementary Information 1. [file 41598_2021_93694_MOESM1_ESM.zip › Supplementary_images of the spectral observations/N136.jpeg]

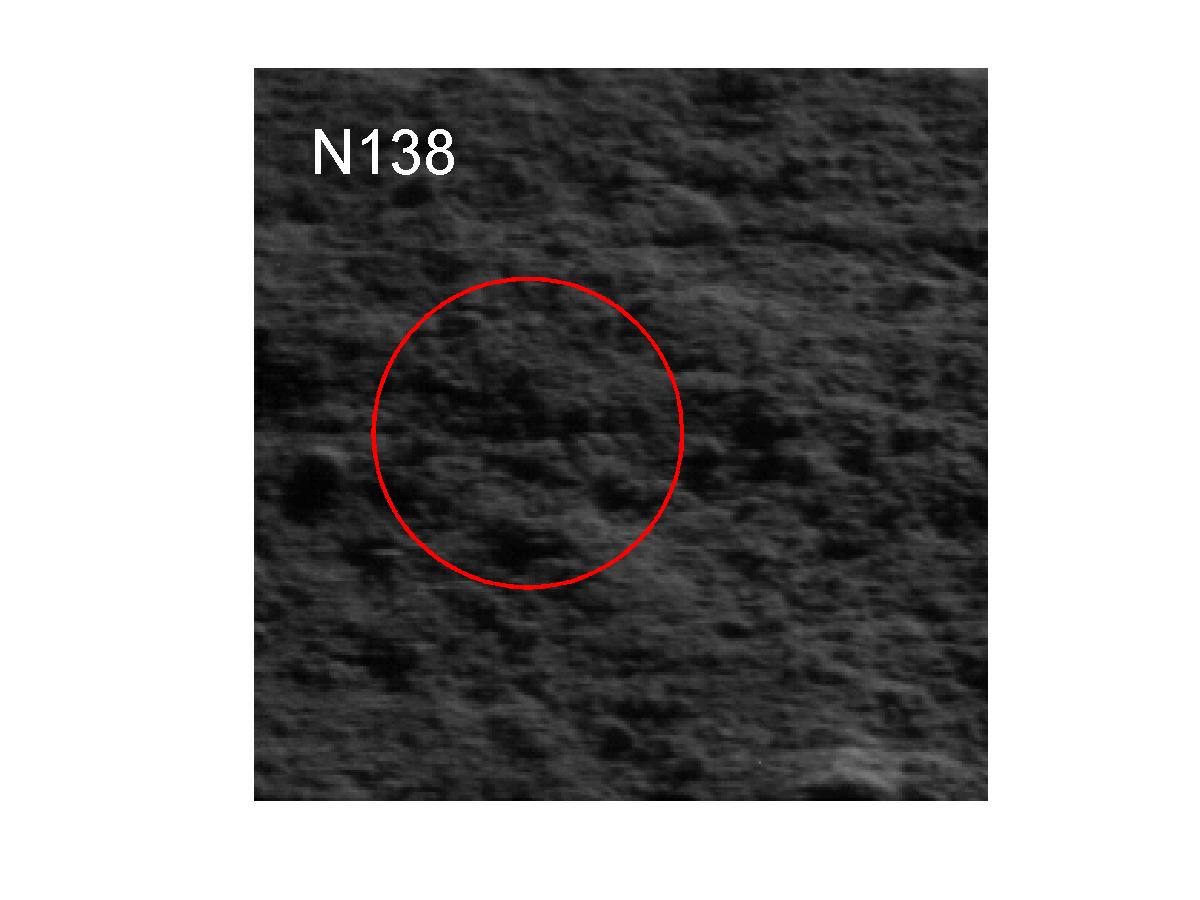

Supplement: Supplementary file 1 — Supplementary Information 1. [file 41598_2021_93694_MOESM1_ESM.zip › Supplementary_images of the spectral observations/N138.jpeg]

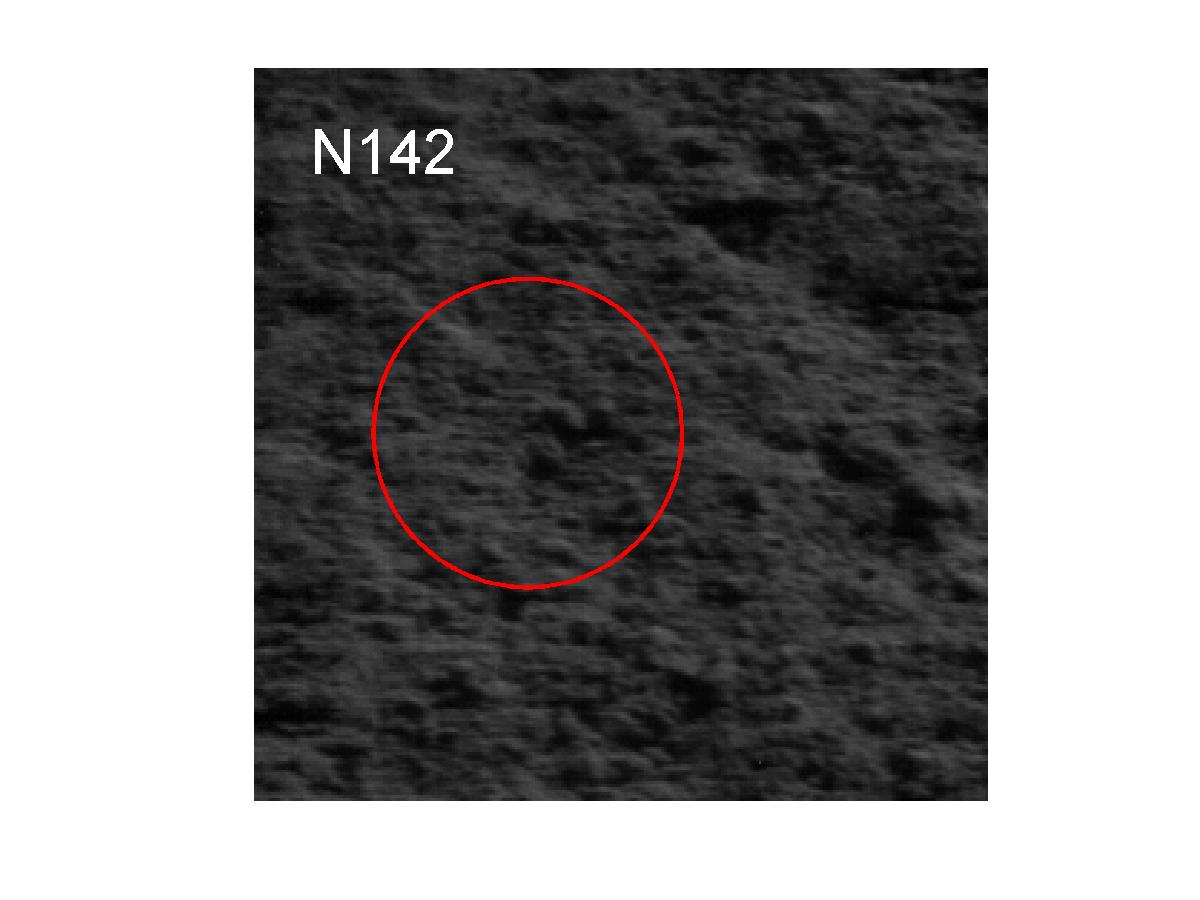

Supplement: Supplementary file 1 — Supplementary Information 1. [file 41598_2021_93694_MOESM1_ESM.zip › Supplementary_images of the spectral observations/N142.jpeg]

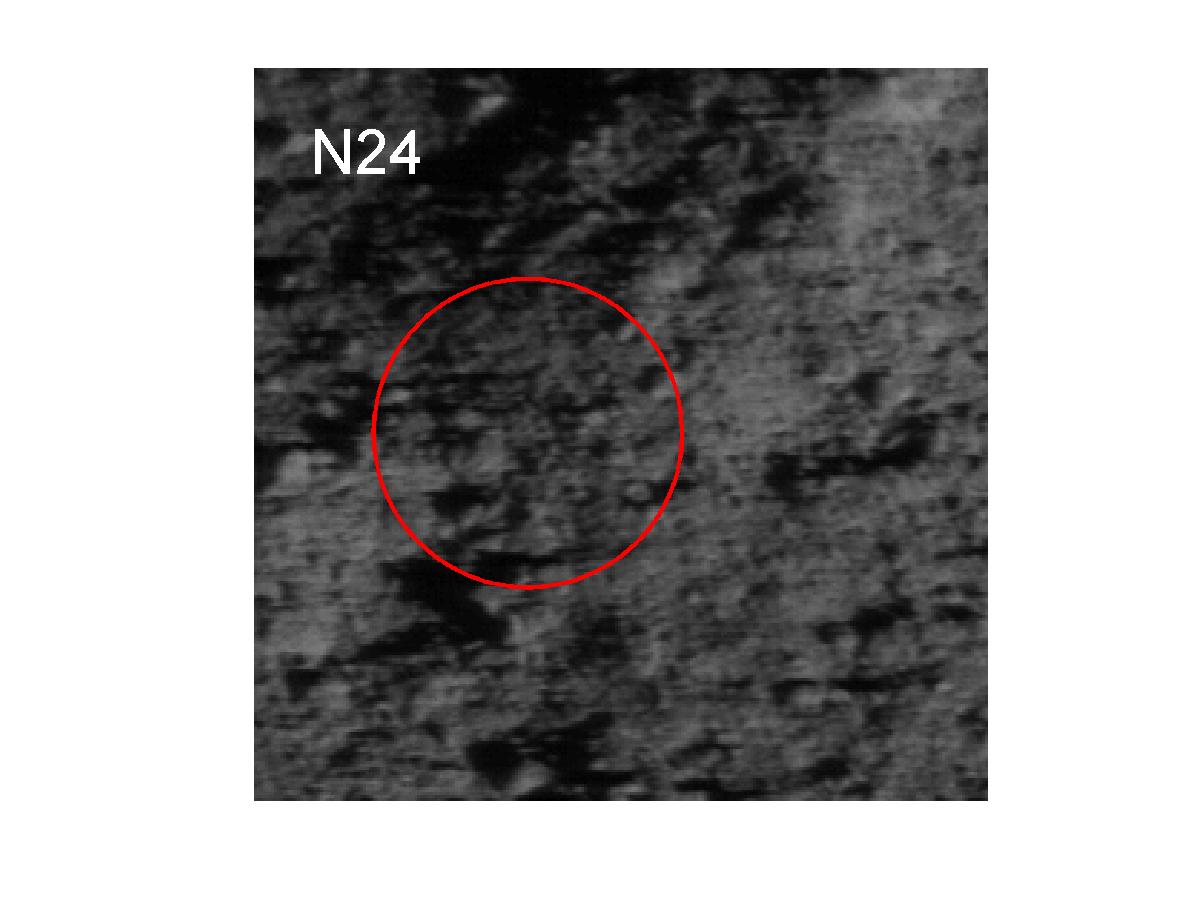

Supplement: Supplementary file 1 — Supplementary Information 1. [file 41598_2021_93694_MOESM1_ESM.zip › Supplementary_images of the spectral observations/N24.jpeg]

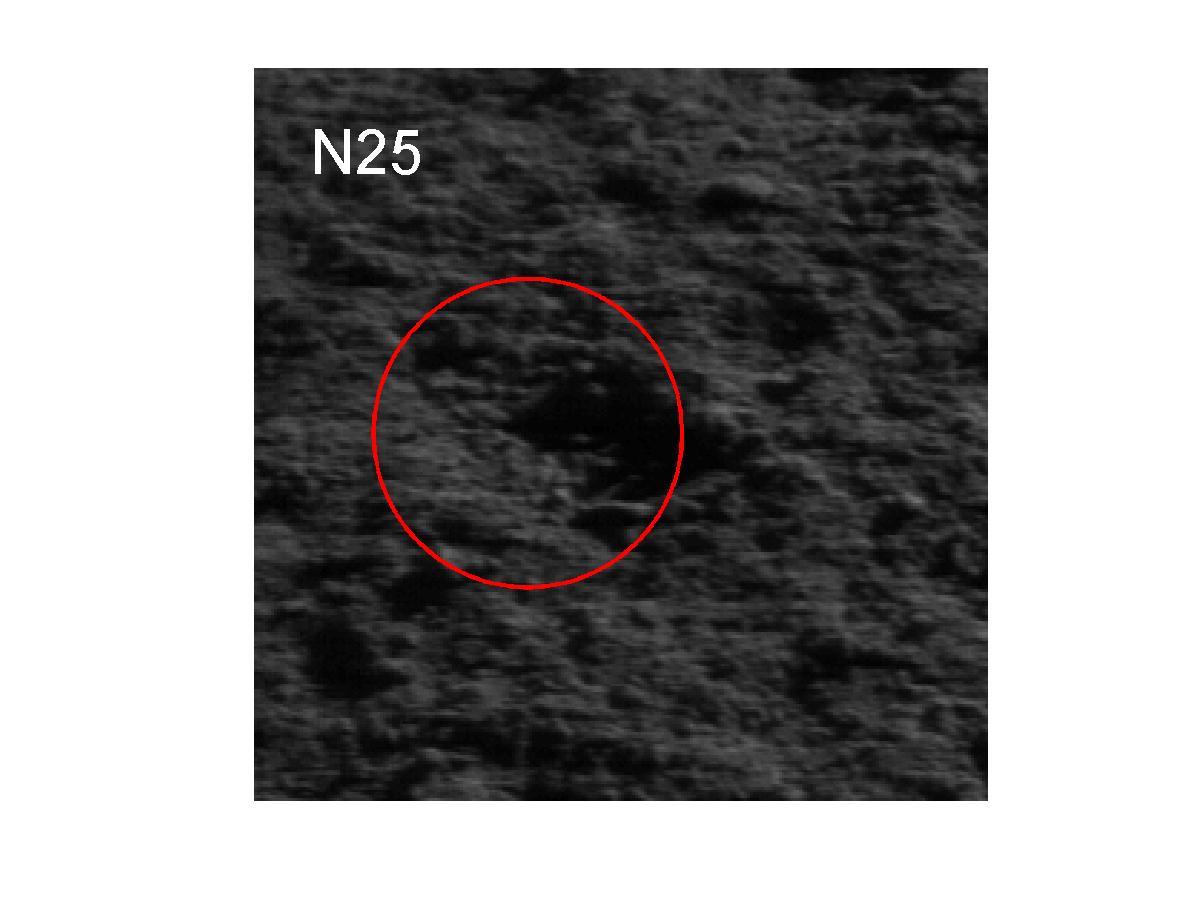

Supplement: Supplementary file 1 — Supplementary Information 1. [file 41598_2021_93694_MOESM1_ESM.zip › Supplementary_images of the spectral observations/N25.jpeg]

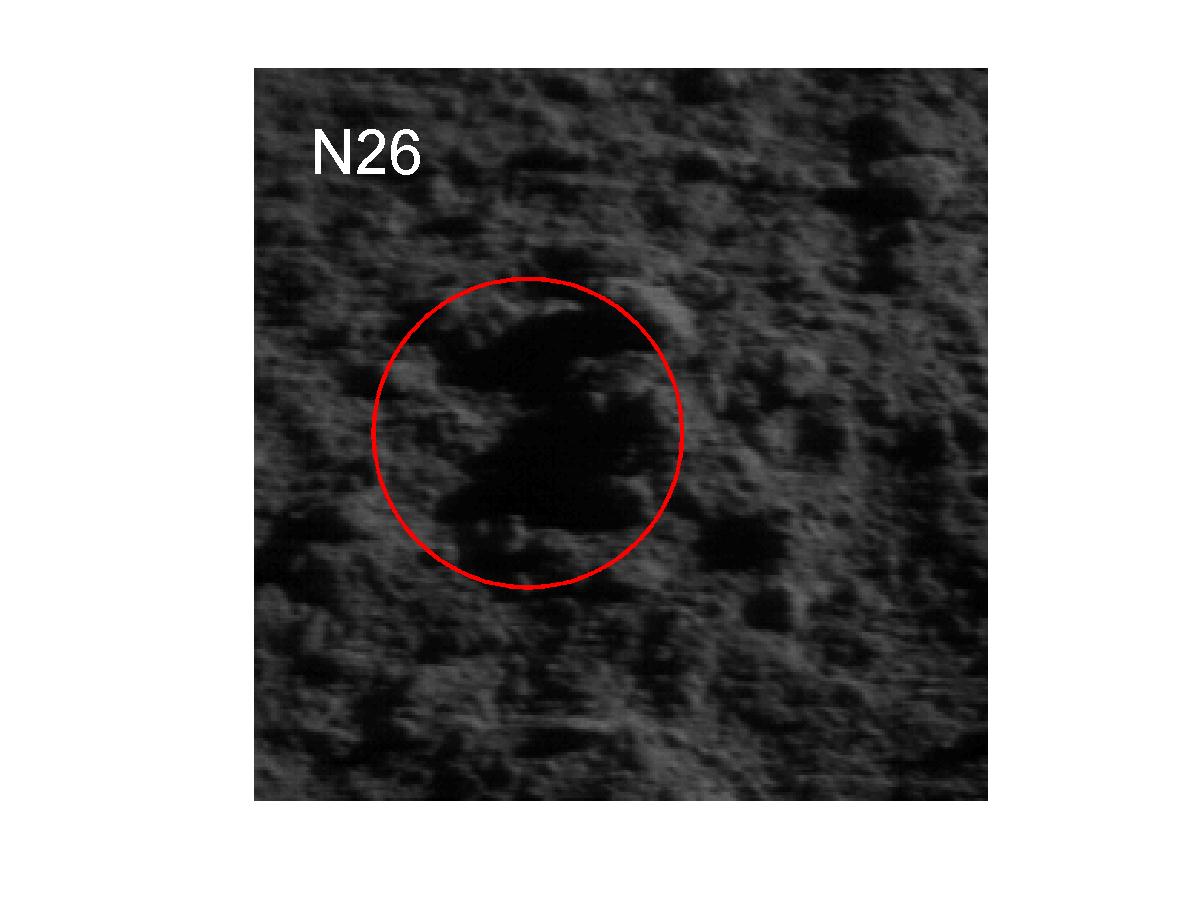

Supplement: Supplementary file 1 — Supplementary Information 1. [file 41598_2021_93694_MOESM1_ESM.zip › Supplementary_images of the spectral observations/N26.jpeg]

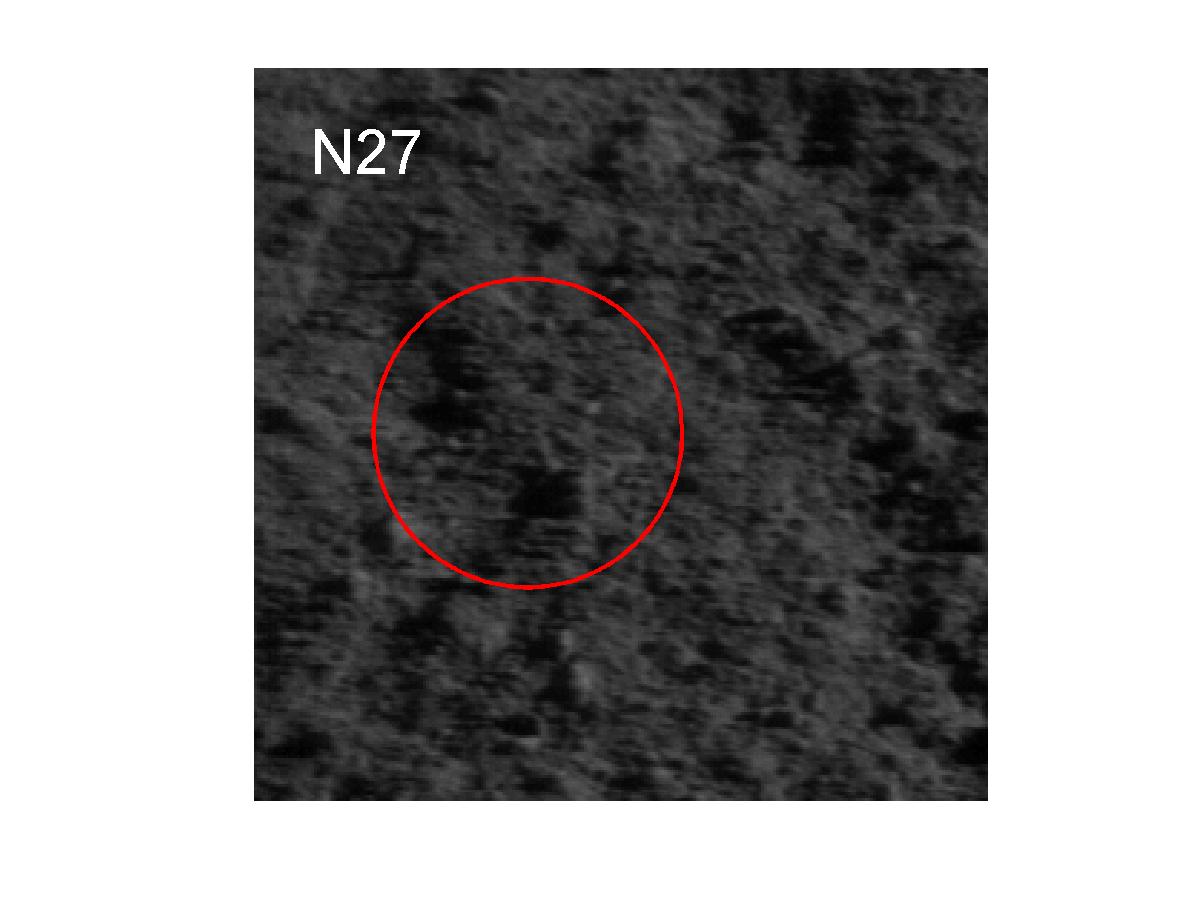

Supplement: Supplementary file 1 — Supplementary Information 1. [file 41598_2021_93694_MOESM1_ESM.zip › Supplementary_images of the spectral observations/N27.jpeg]

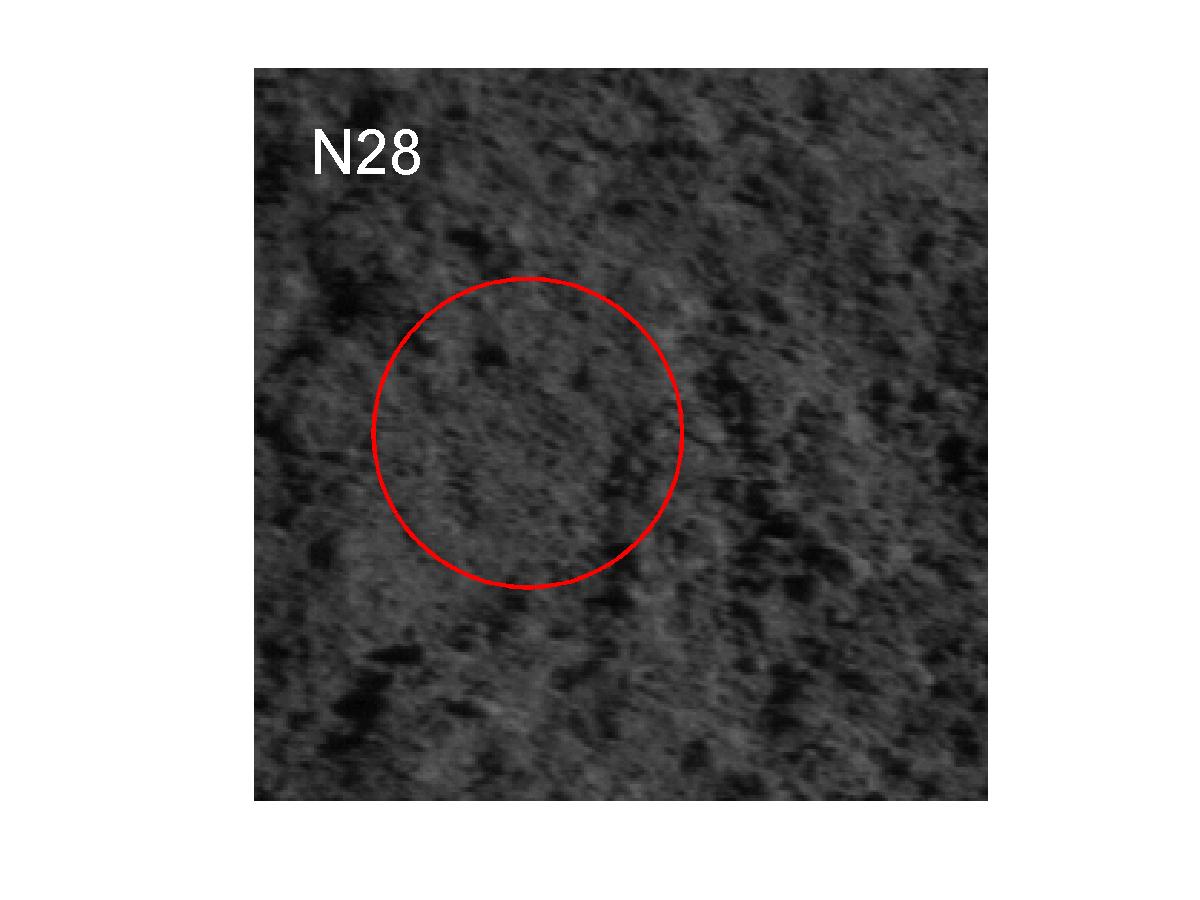

Supplement: Supplementary file 1 — Supplementary Information 1. [file 41598_2021_93694_MOESM1_ESM.zip › Supplementary_images of the spectral observations/N28.jpeg]

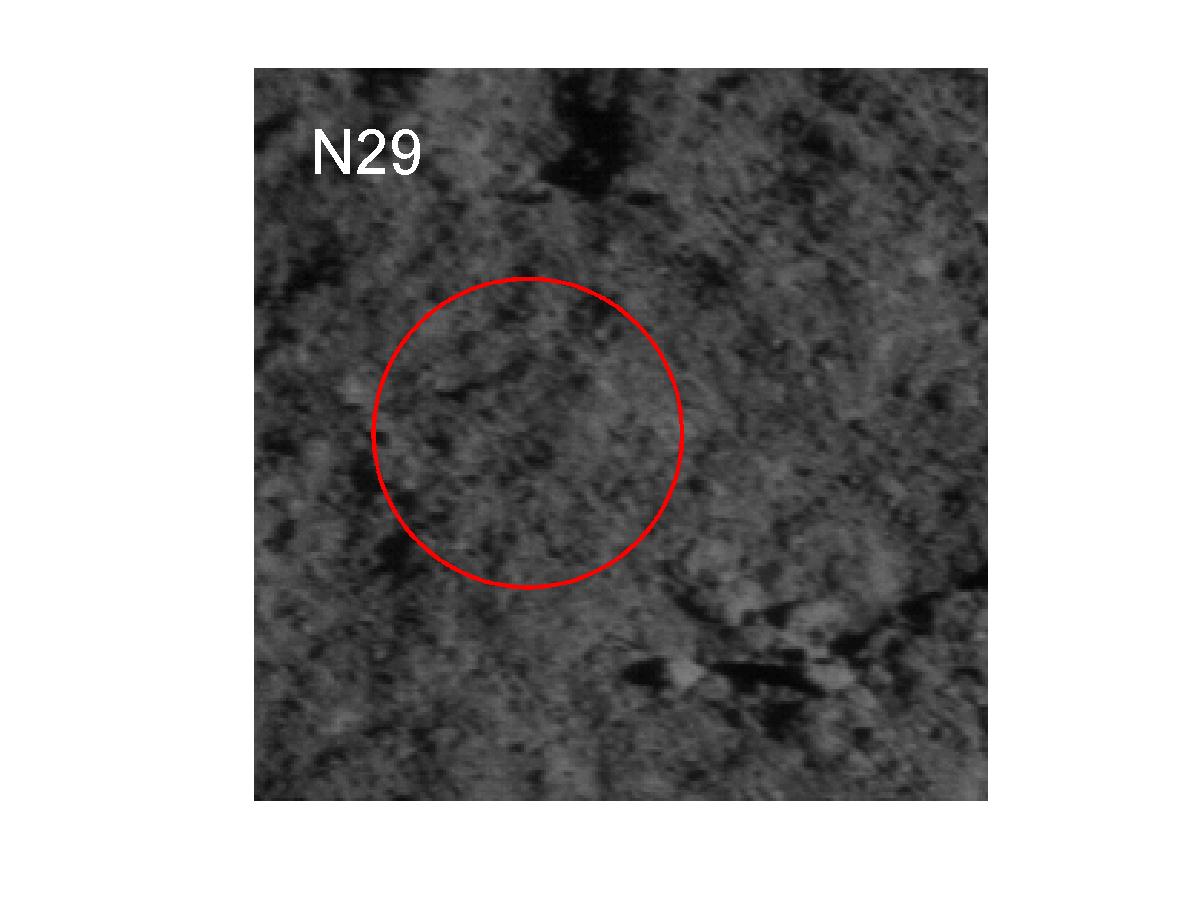

Supplement: Supplementary file 1 — Supplementary Information 1. [file 41598_2021_93694_MOESM1_ESM.zip › Supplementary_images of the spectral observations/N29.jpeg]

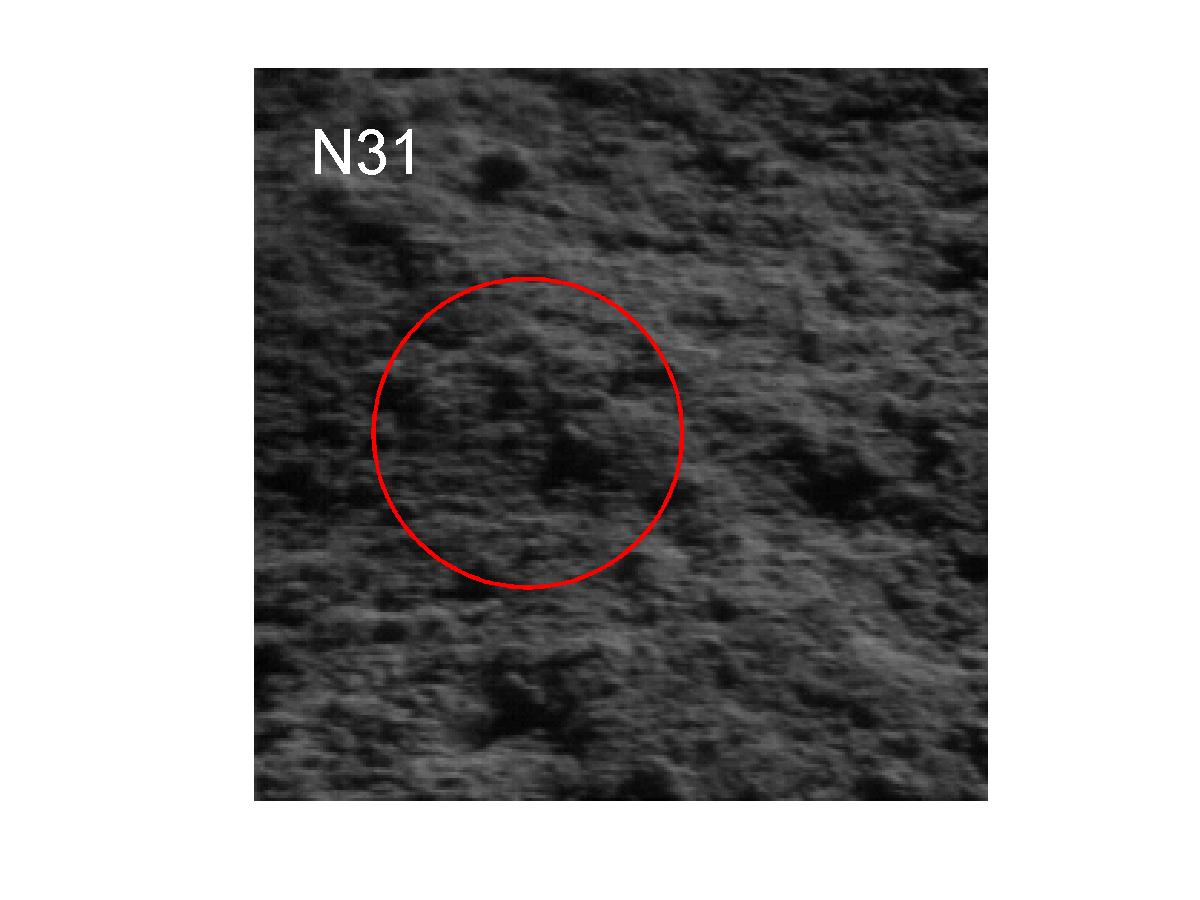

Supplement: Supplementary file 1 — Supplementary Information 1. [file 41598_2021_93694_MOESM1_ESM.zip › Supplementary_images of the spectral observations/N31.jpeg]

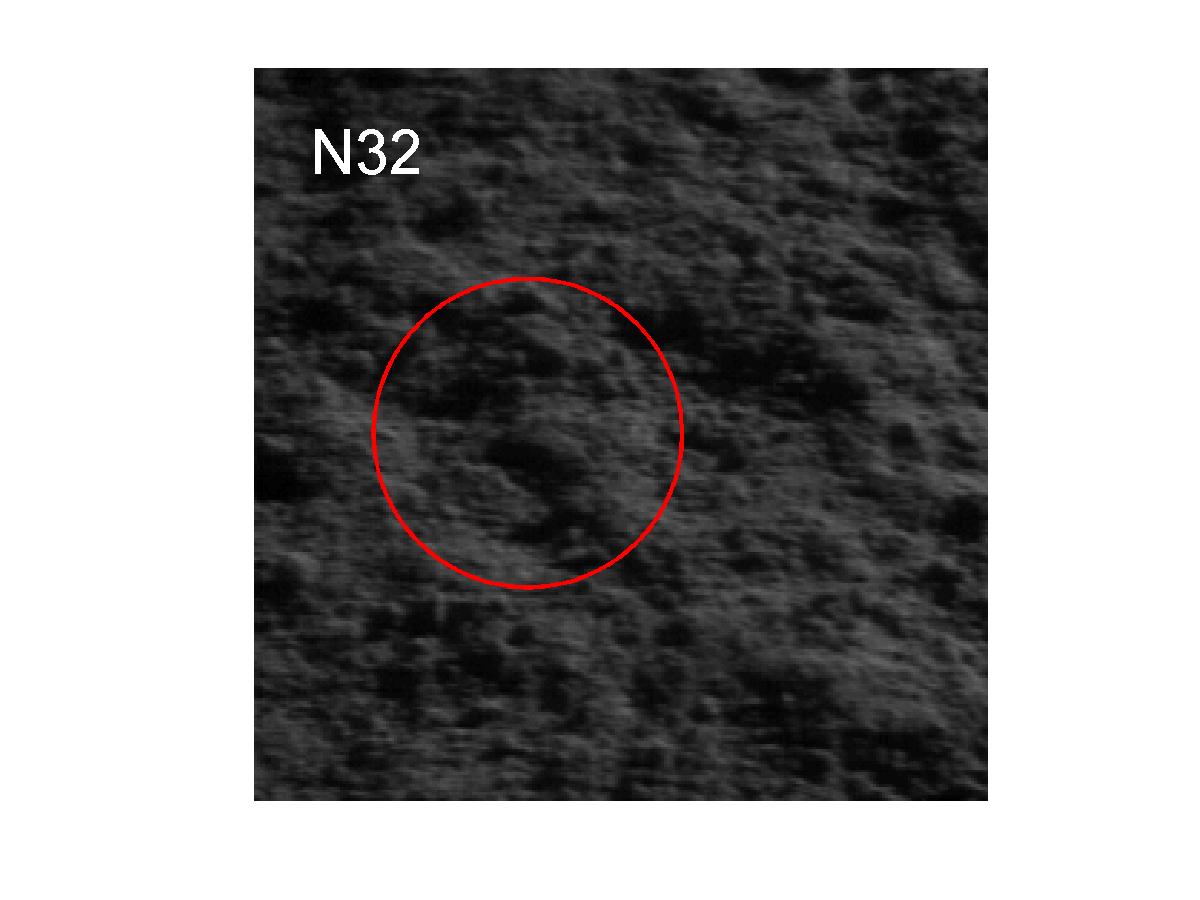

Supplement: Supplementary file 1 — Supplementary Information 1. [file 41598_2021_93694_MOESM1_ESM.zip › Supplementary_images of the spectral observations/N32.jpeg]

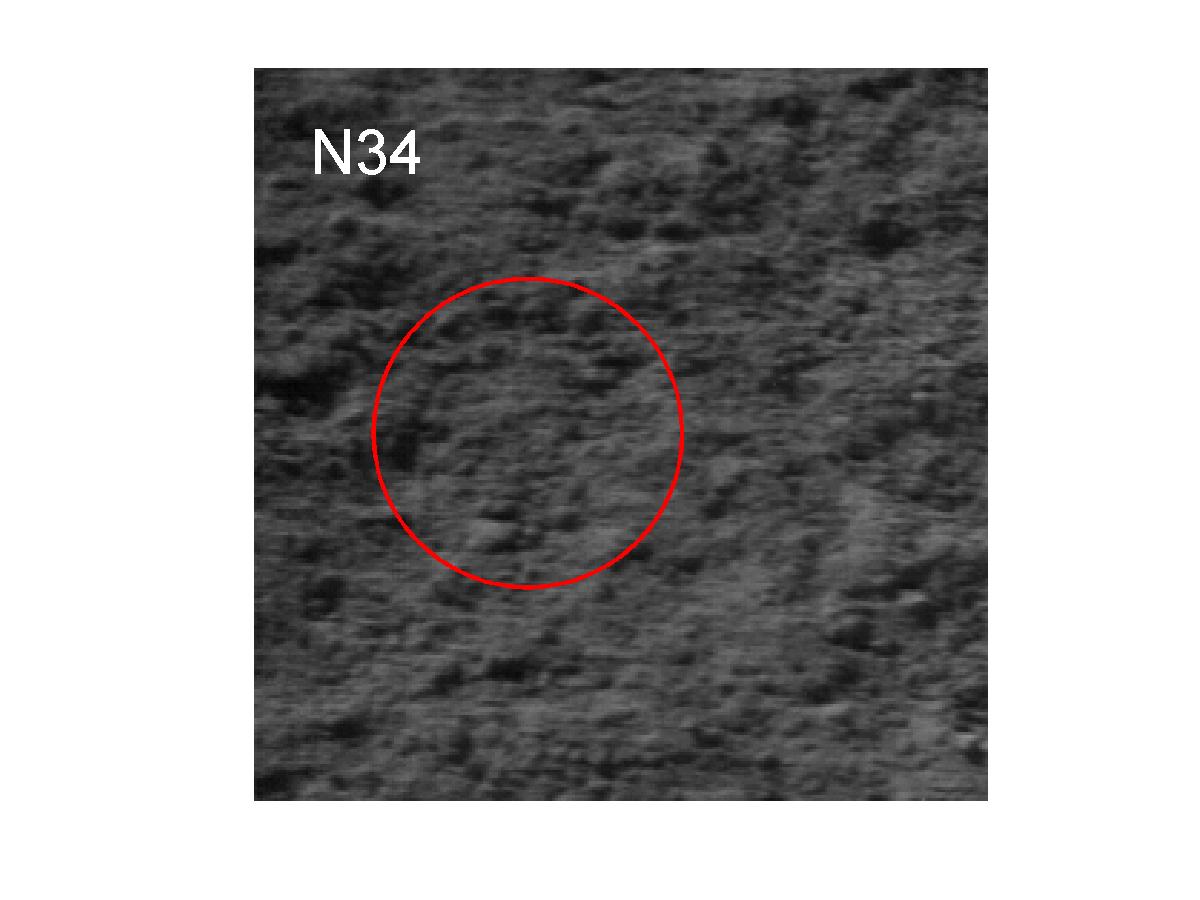

Supplement: Supplementary file 1 — Supplementary Information 1. [file 41598_2021_93694_MOESM1_ESM.zip › Supplementary_images of the spectral observations/N34.jpeg]

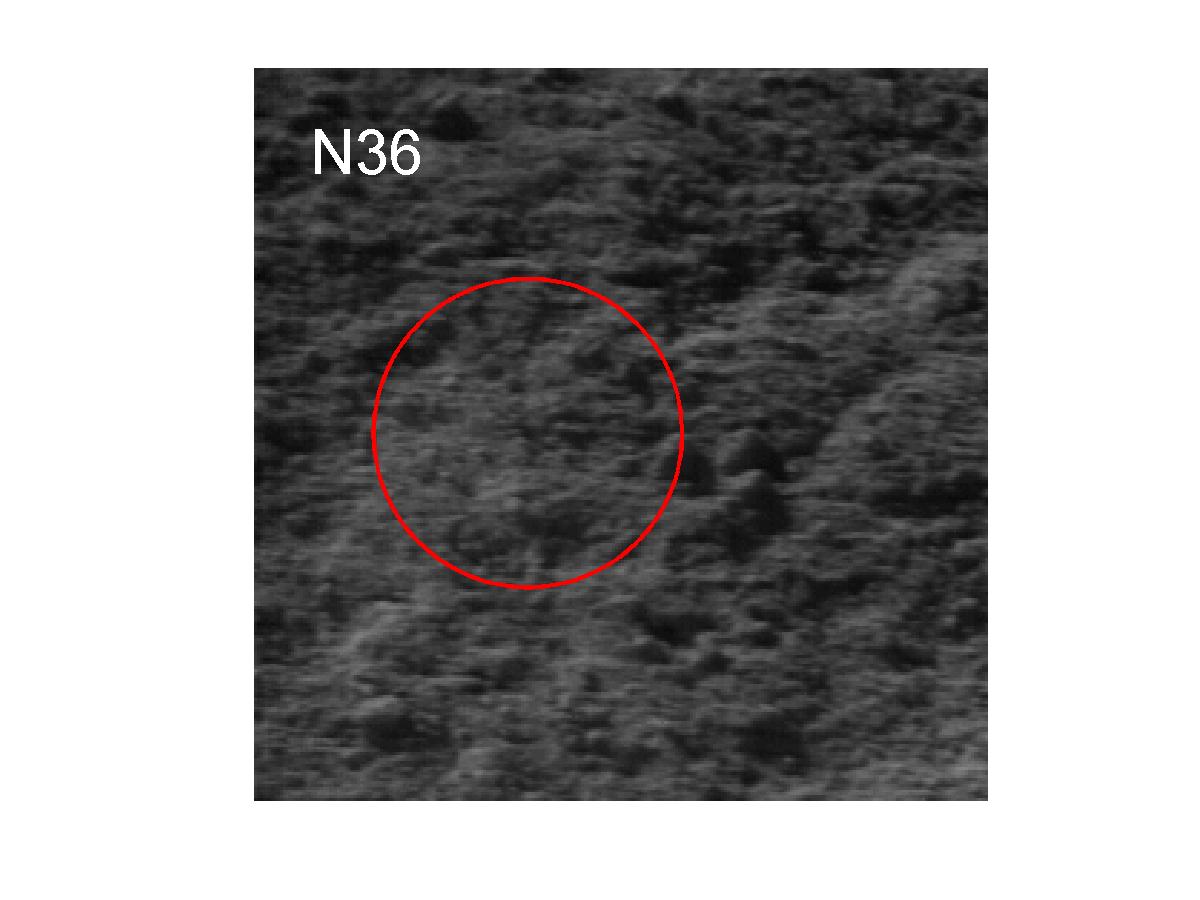

Supplement: Supplementary file 1 — Supplementary Information 1. [file 41598_2021_93694_MOESM1_ESM.zip › Supplementary_images of the spectral observations/N36.jpeg]

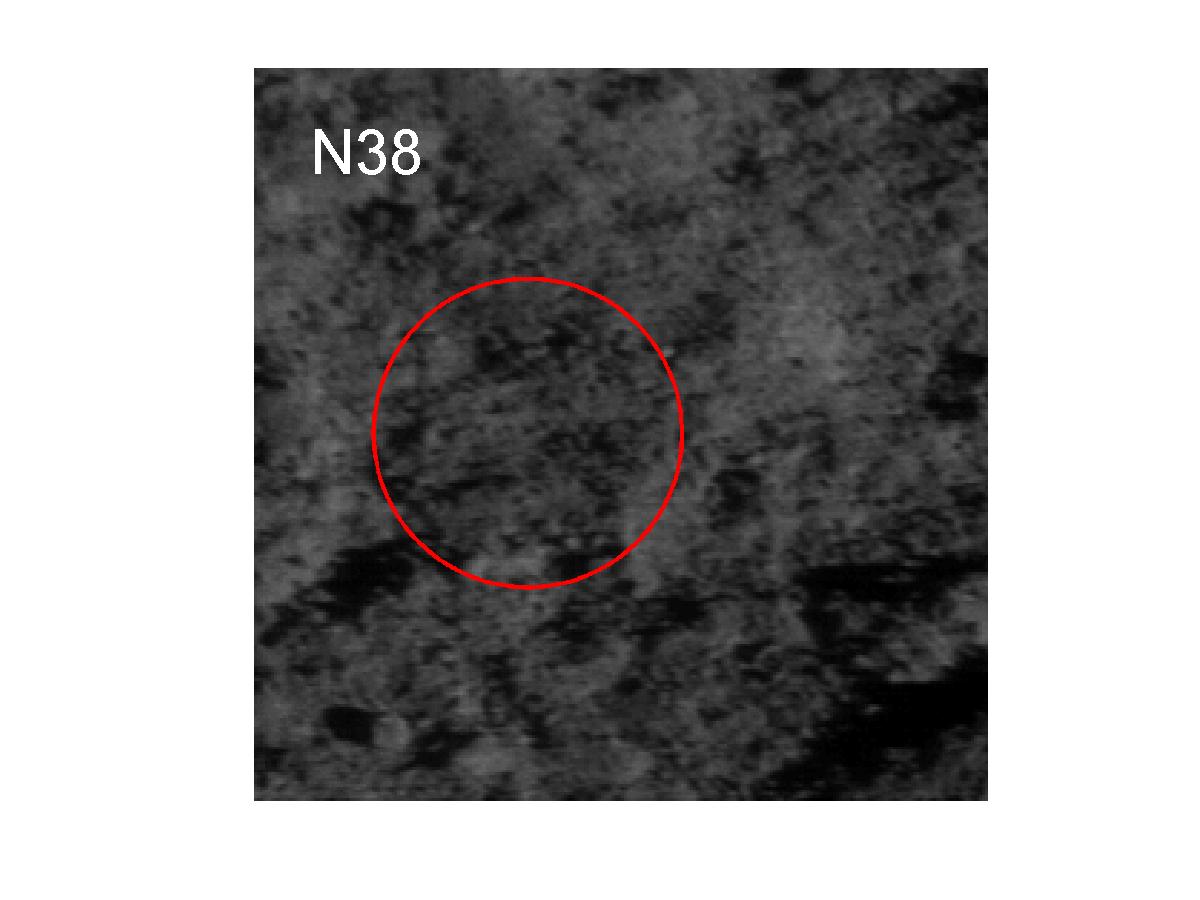

Supplement: Supplementary file 1 — Supplementary Information 1. [file 41598_2021_93694_MOESM1_ESM.zip › Supplementary_images of the spectral observations/N38.jpeg]

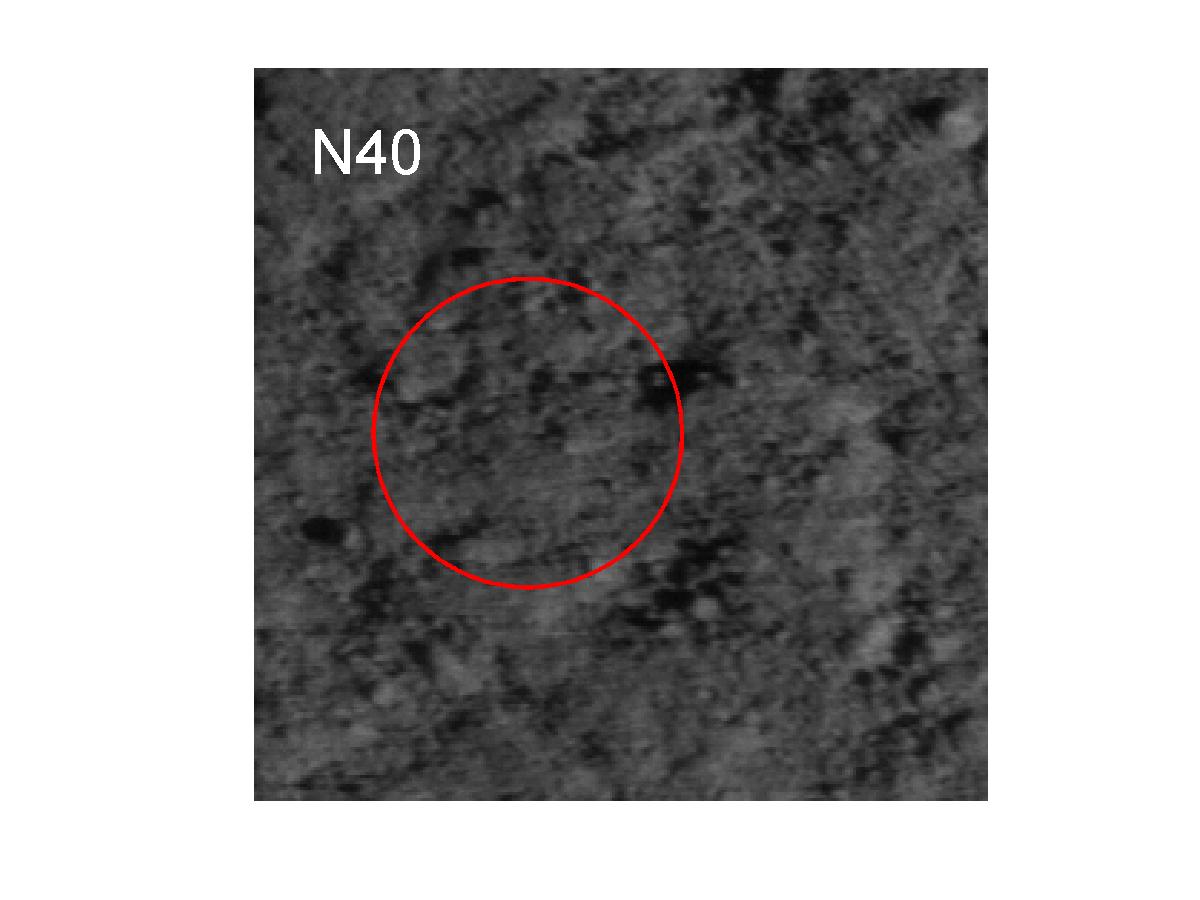

Supplement: Supplementary file 1 — Supplementary Information 1. [file 41598_2021_93694_MOESM1_ESM.zip › Supplementary_images of the spectral observations/N40.jpeg]

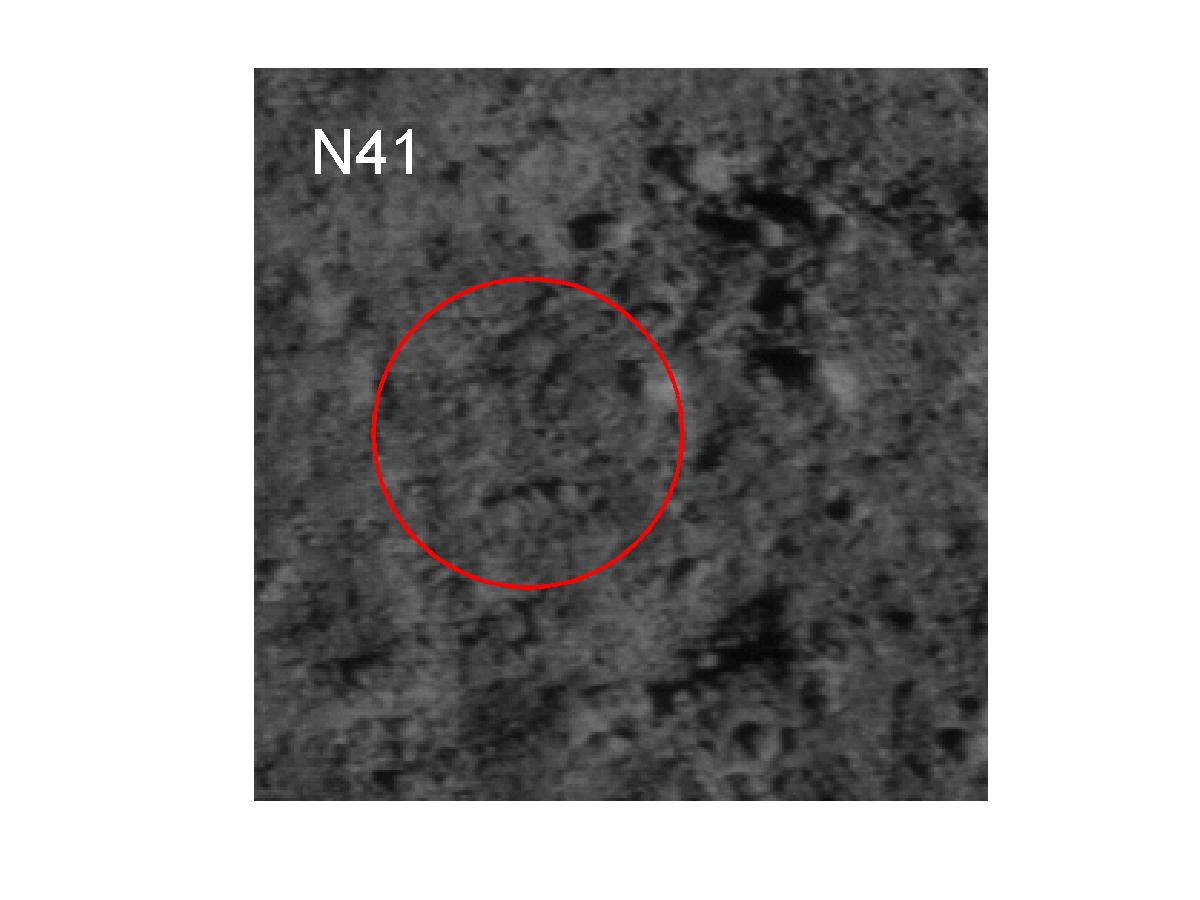

Supplement: Supplementary file 1 — Supplementary Information 1. [file 41598_2021_93694_MOESM1_ESM.zip › Supplementary_images of the spectral observations/N41.jpeg]

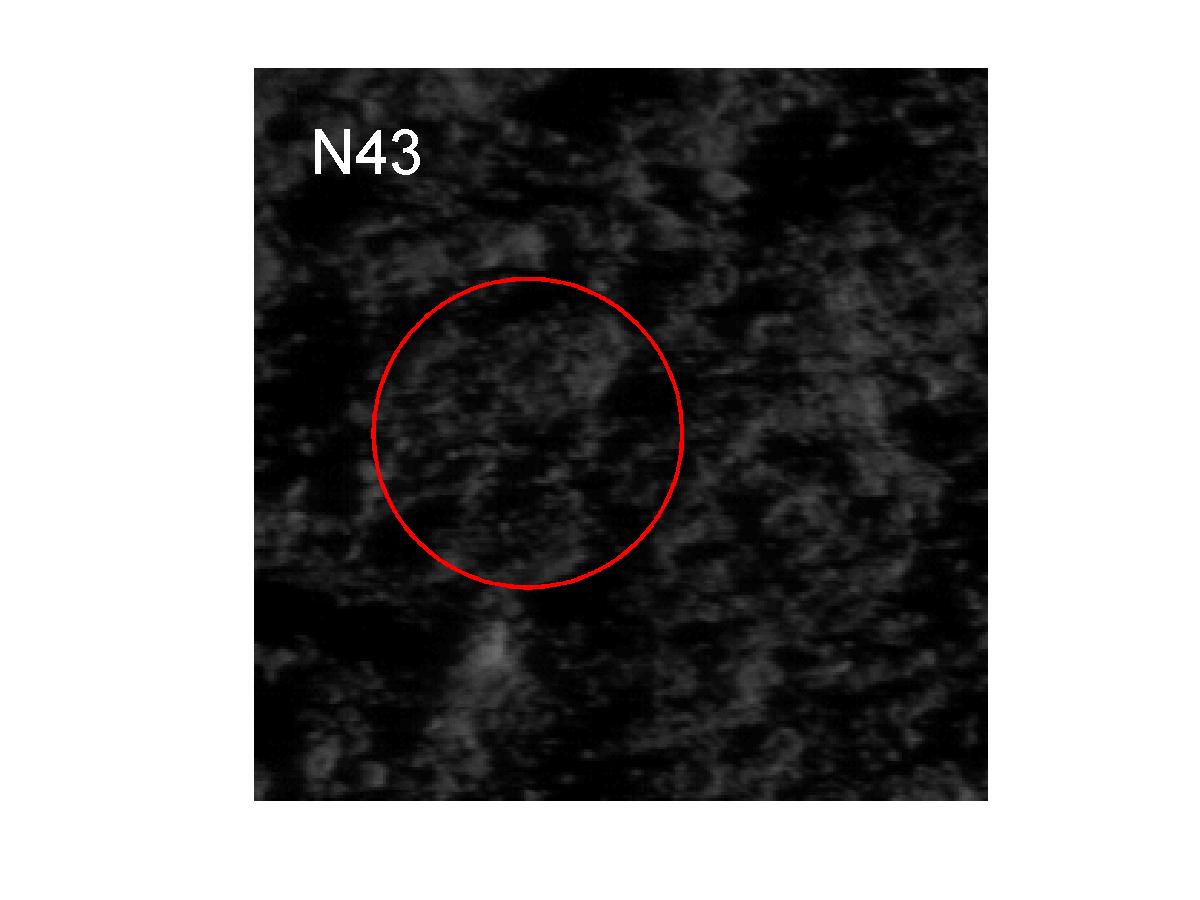

Supplement: Supplementary file 1 — Supplementary Information 1. [file 41598_2021_93694_MOESM1_ESM.zip › Supplementary_images of the spectral observations/N43.jpeg]

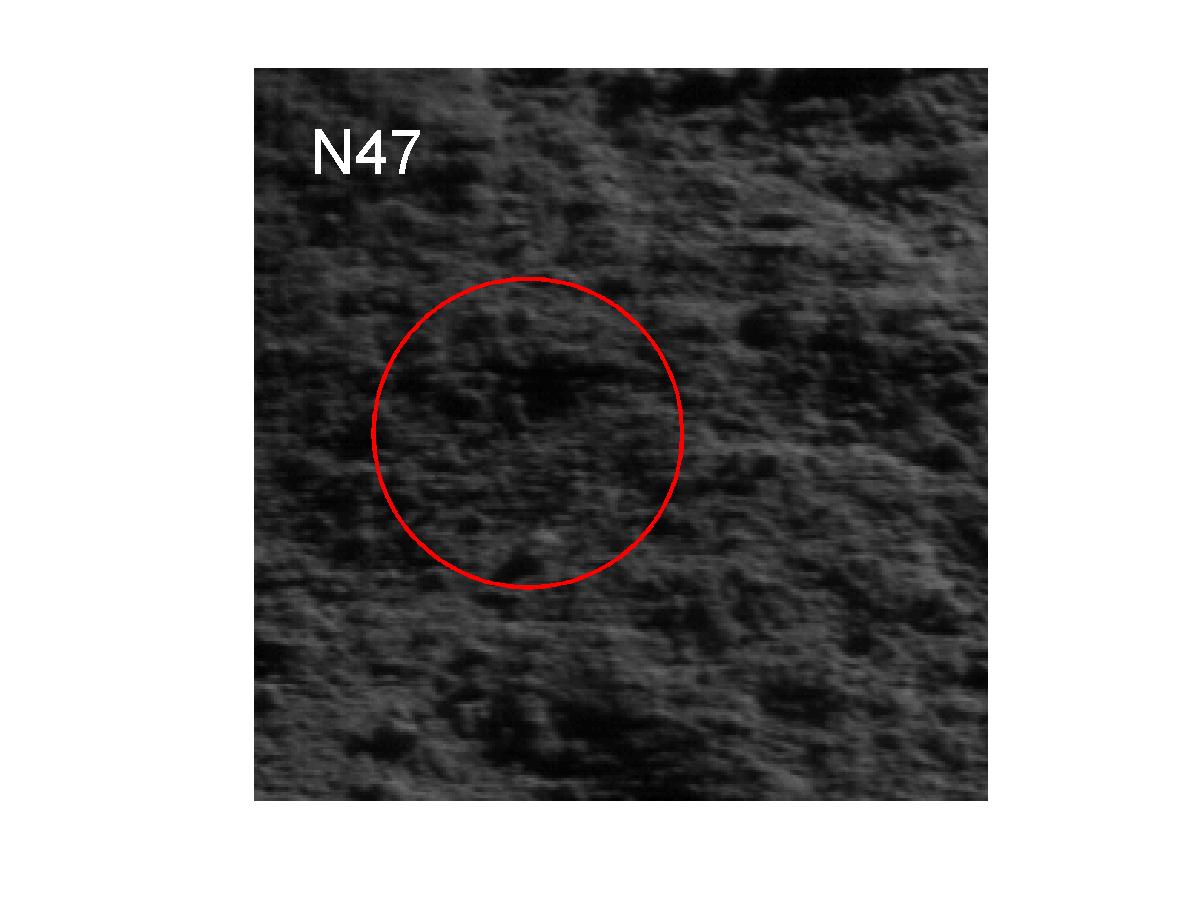

Supplement: Supplementary file 1 — Supplementary Information 1. [file 41598_2021_93694_MOESM1_ESM.zip › Supplementary_images of the spectral observations/N47.jpeg]

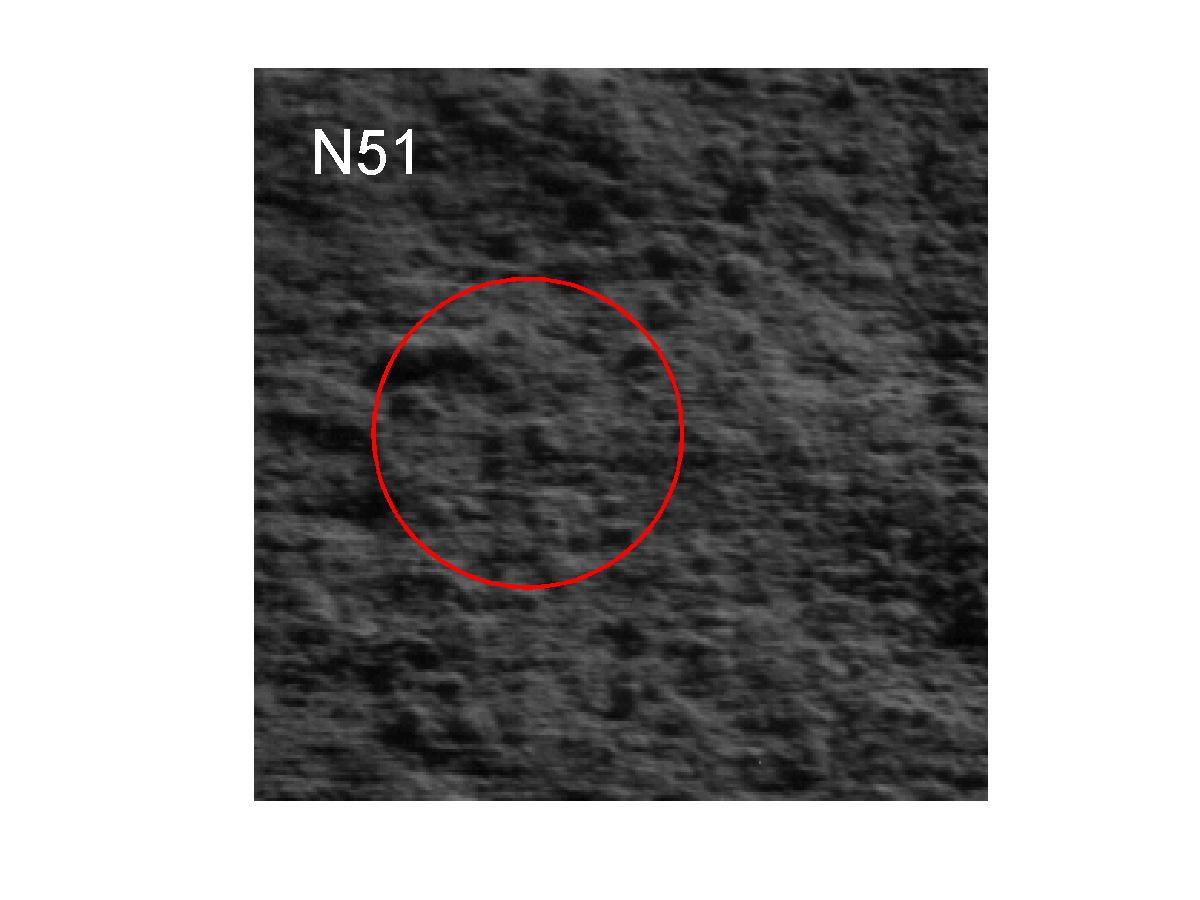

Supplement: Supplementary file 1 — Supplementary Information 1. [file 41598_2021_93694_MOESM1_ESM.zip › Supplementary_images of the spectral observations/N51.jpeg]

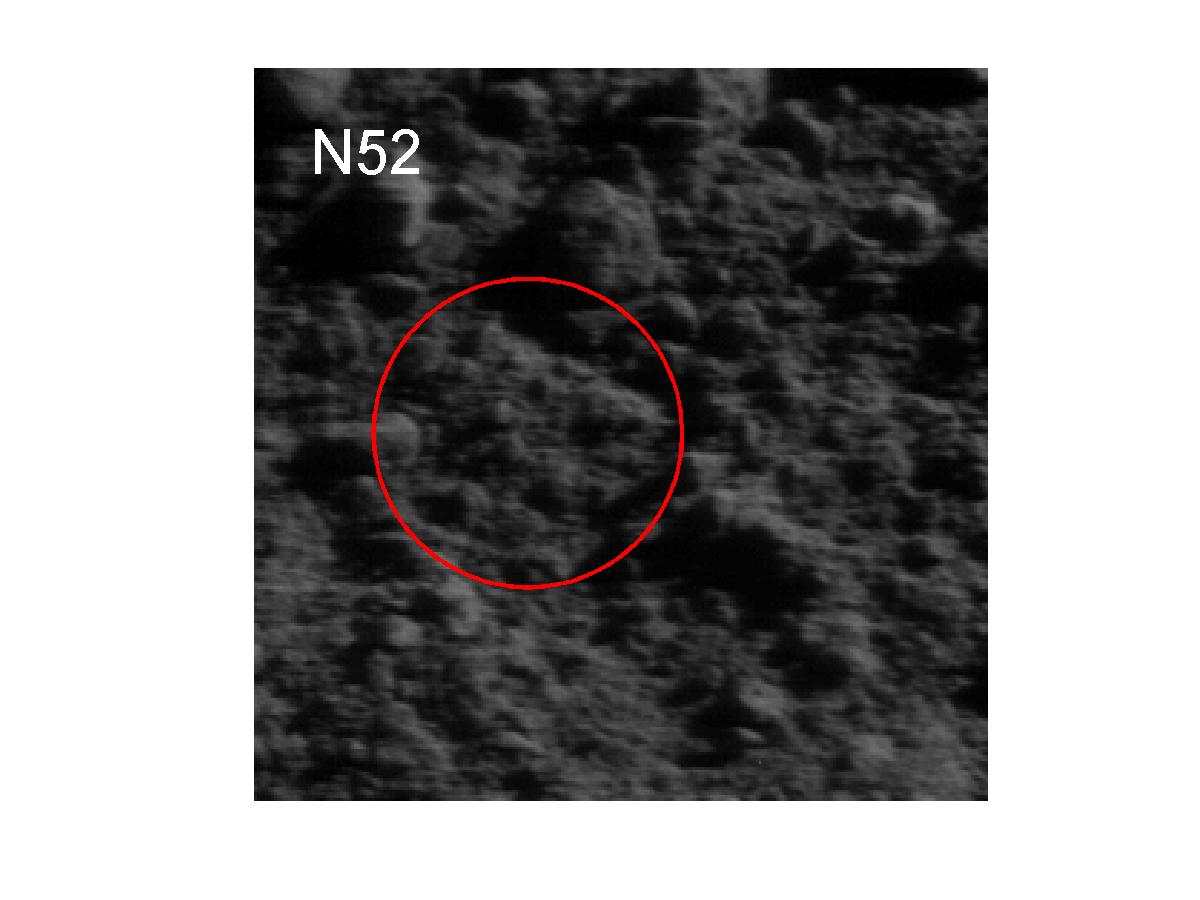

Supplement: Supplementary file 1 — Supplementary Information 1. [file 41598_2021_93694_MOESM1_ESM.zip › Supplementary_images of the spectral observations/N52.jpeg]

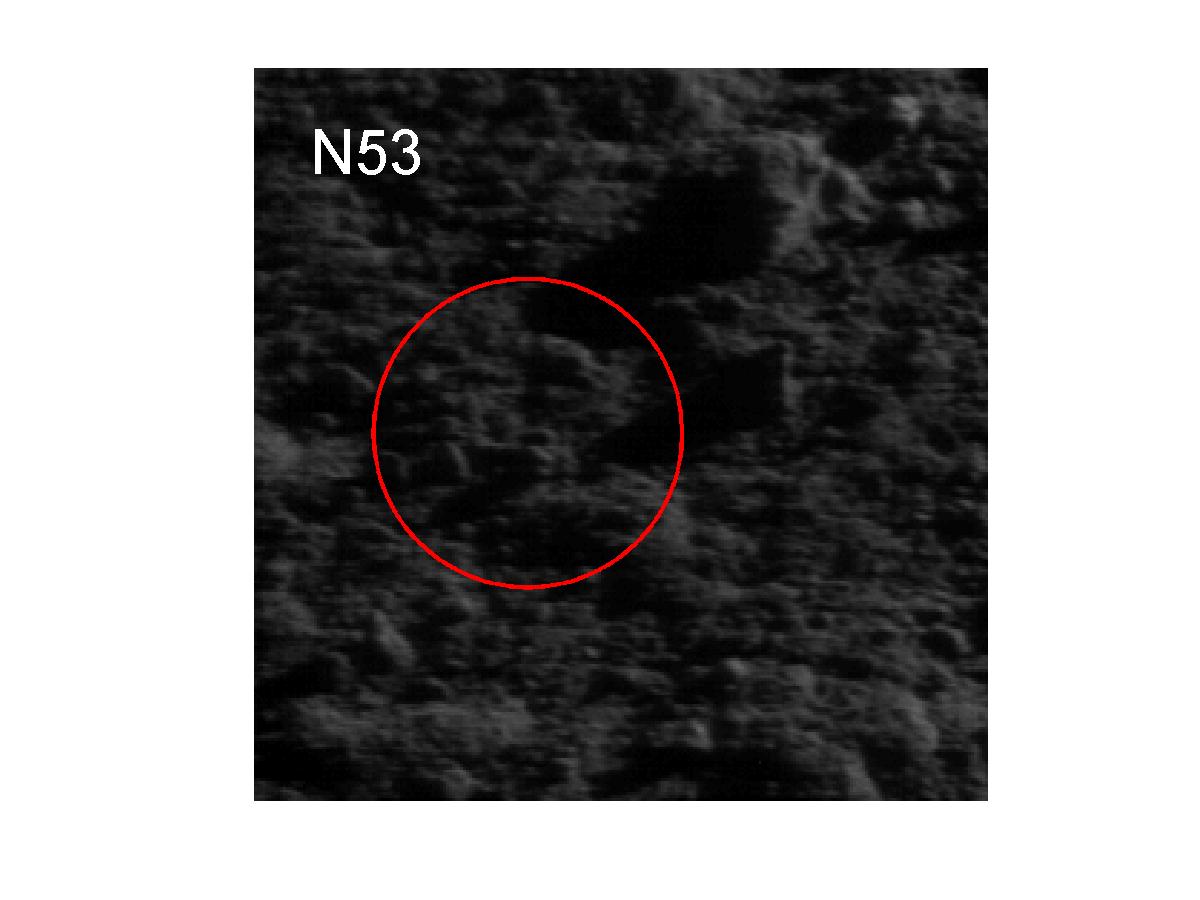

Supplement: Supplementary file 1 — Supplementary Information 1. [file 41598_2021_93694_MOESM1_ESM.zip › Supplementary_images of the spectral observations/N53.jpeg]

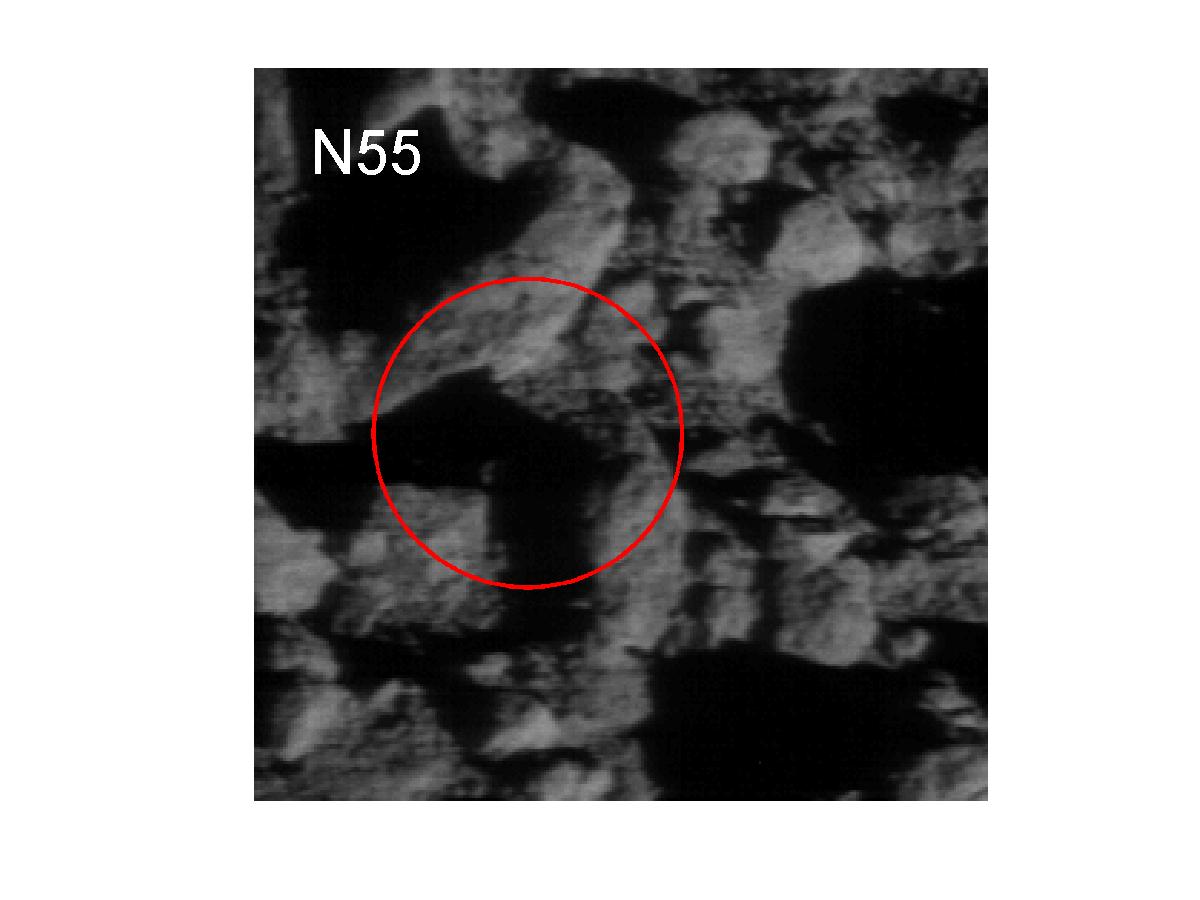

Supplement: Supplementary file 1 — Supplementary Information 1. [file 41598_2021_93694_MOESM1_ESM.zip › Supplementary_images of the spectral observations/N55.jpeg]

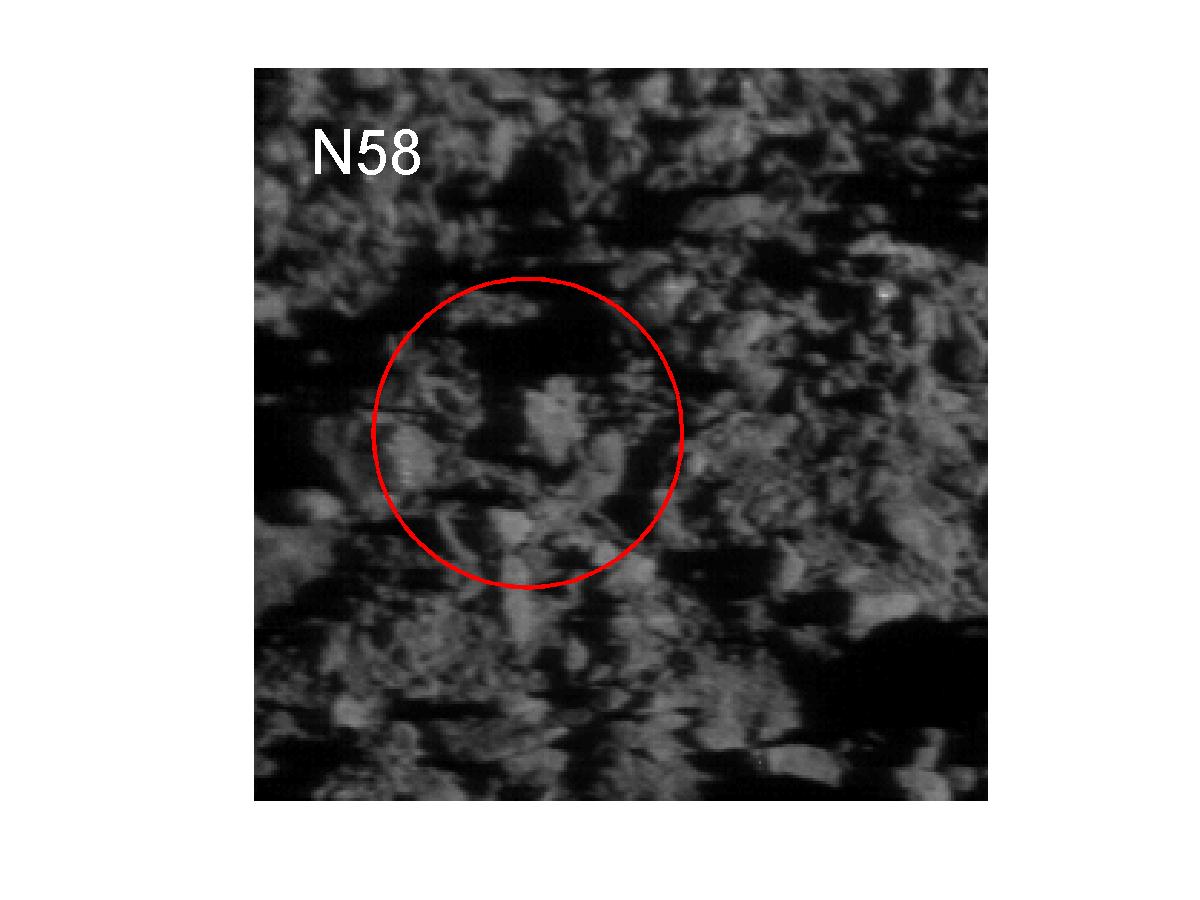

Supplement: Supplementary file 1 — Supplementary Information 1. [file 41598_2021_93694_MOESM1_ESM.zip › Supplementary_images of the spectral observations/N58.jpeg]

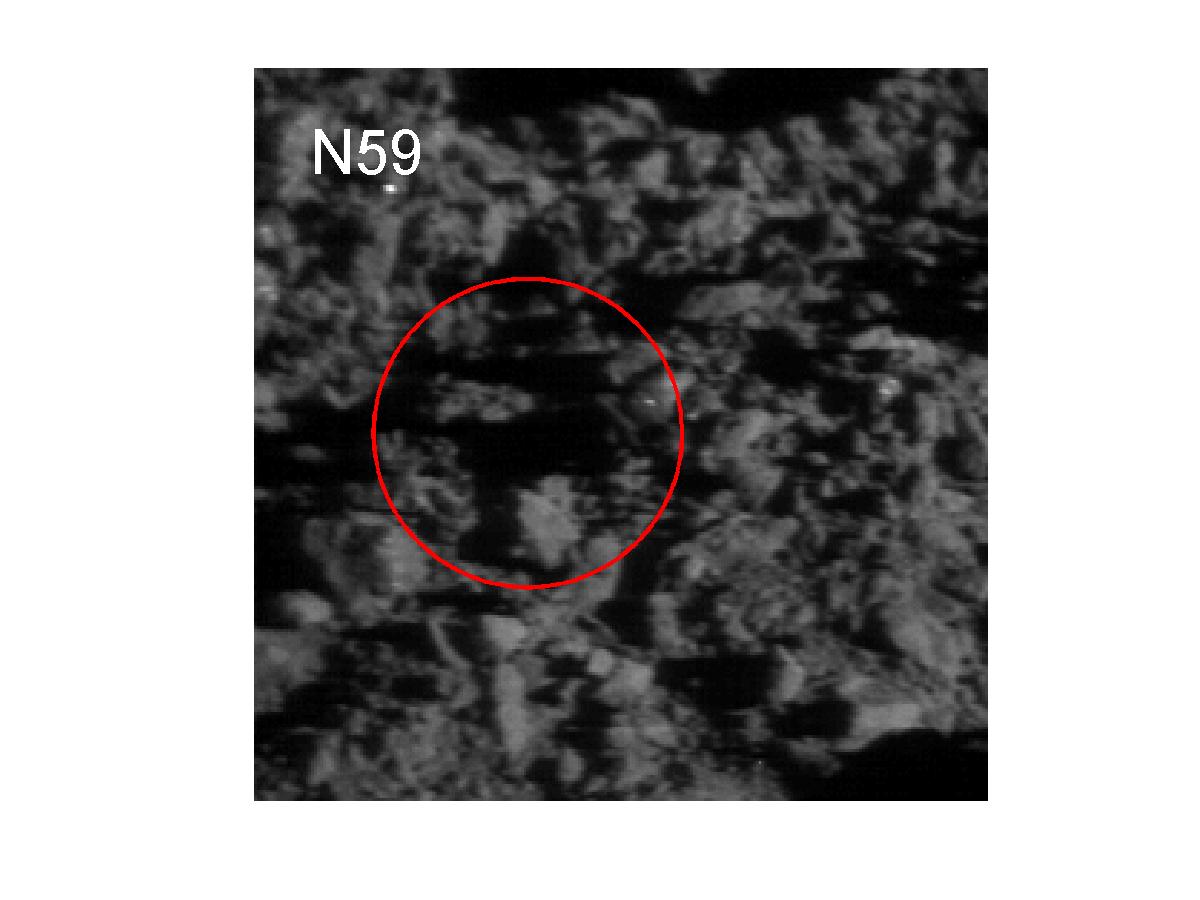

Supplement: Supplementary file 1 — Supplementary Information 1. [file 41598_2021_93694_MOESM1_ESM.zip › Supplementary_images of the spectral observations/N59.jpeg]

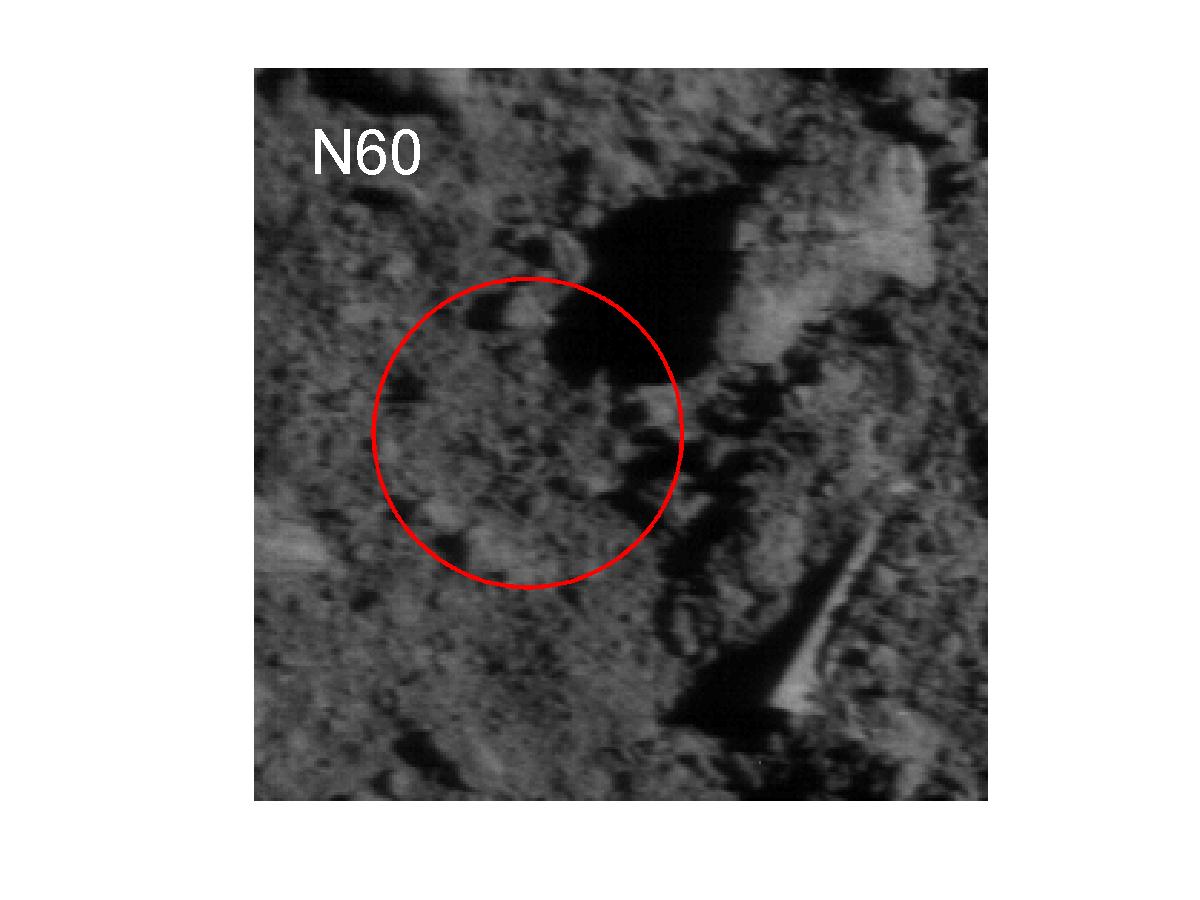

Supplement: Supplementary file 1 — Supplementary Information 1. [file 41598_2021_93694_MOESM1_ESM.zip › Supplementary_images of the spectral observations/N60.jpeg]

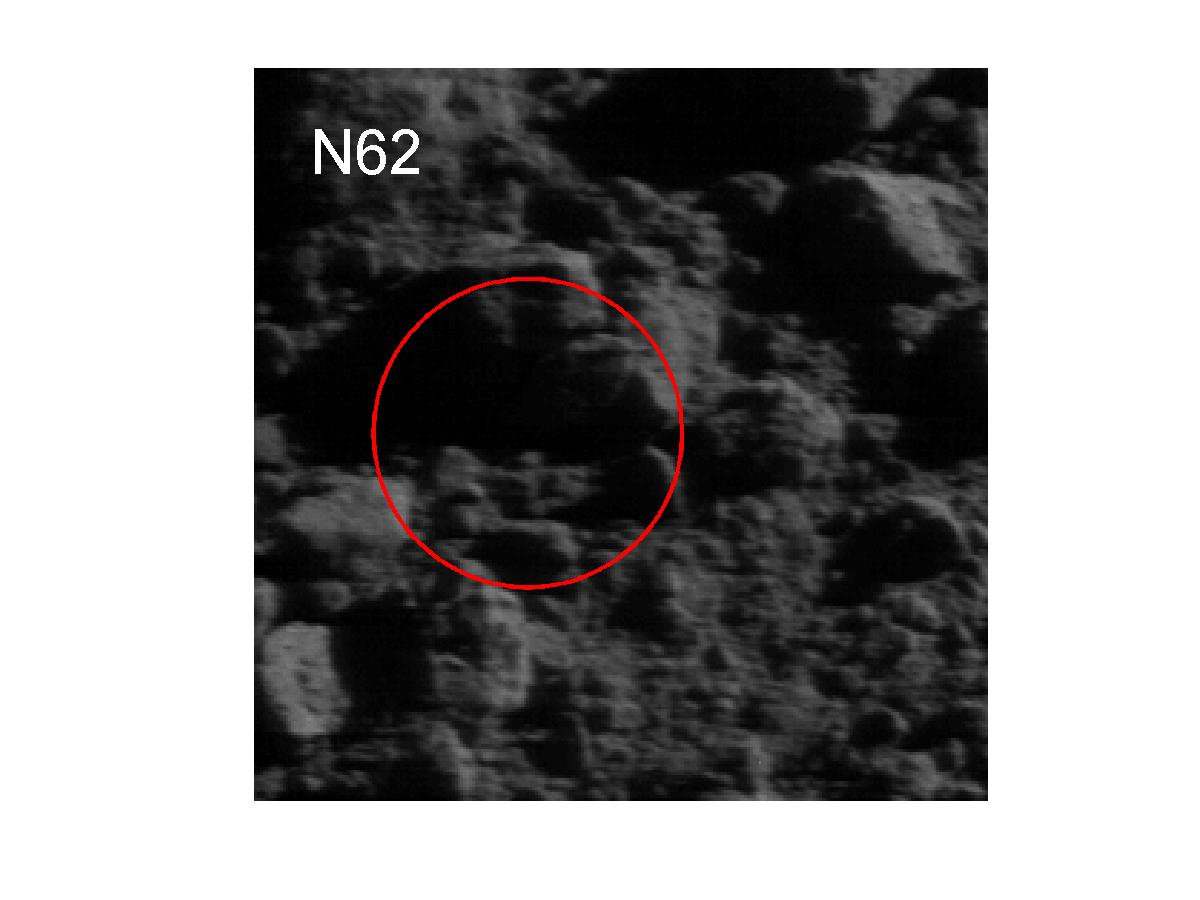

Supplement: Supplementary file 1 — Supplementary Information 1. [file 41598_2021_93694_MOESM1_ESM.zip › Supplementary_images of the spectral observations/N62.jpeg]

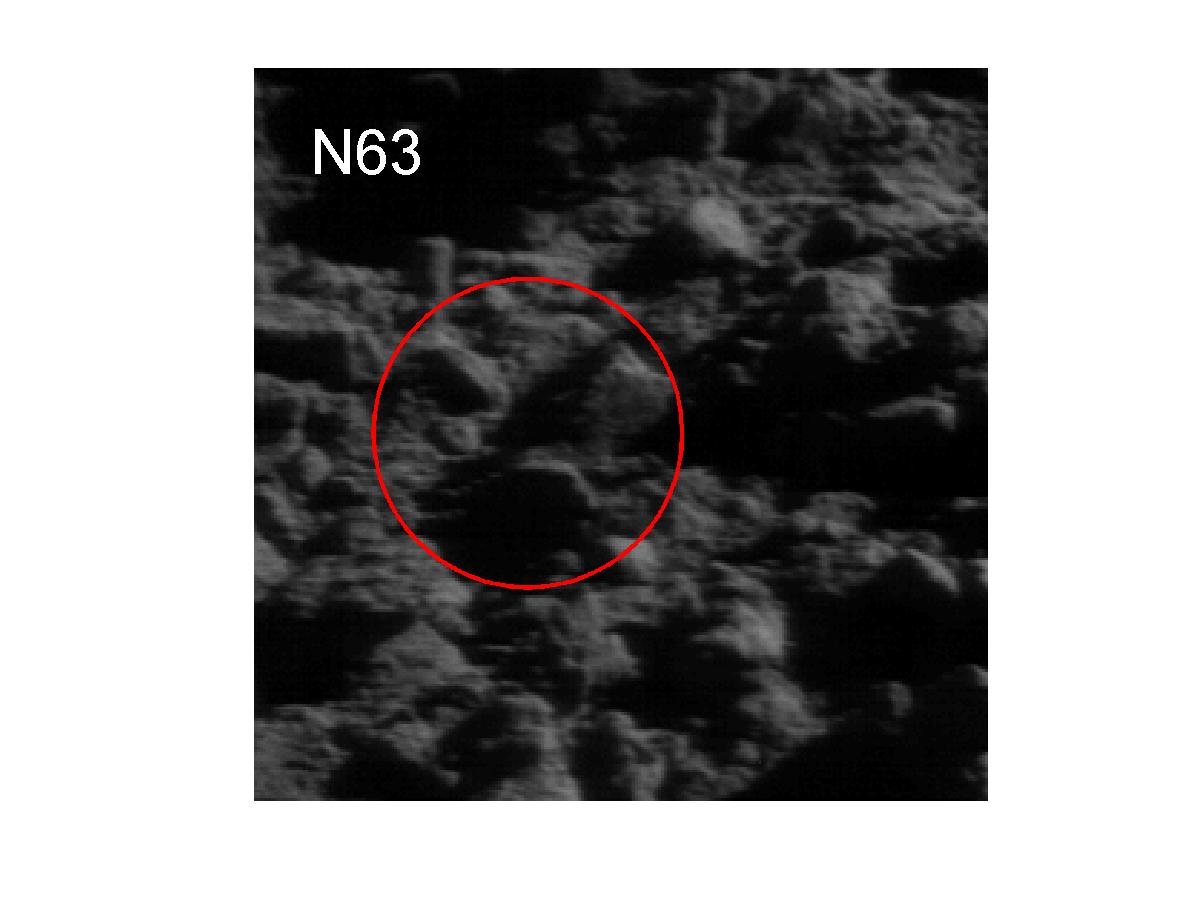

Supplement: Supplementary file 1 — Supplementary Information 1. [file 41598_2021_93694_MOESM1_ESM.zip › Supplementary_images of the spectral observations/N63.jpeg]

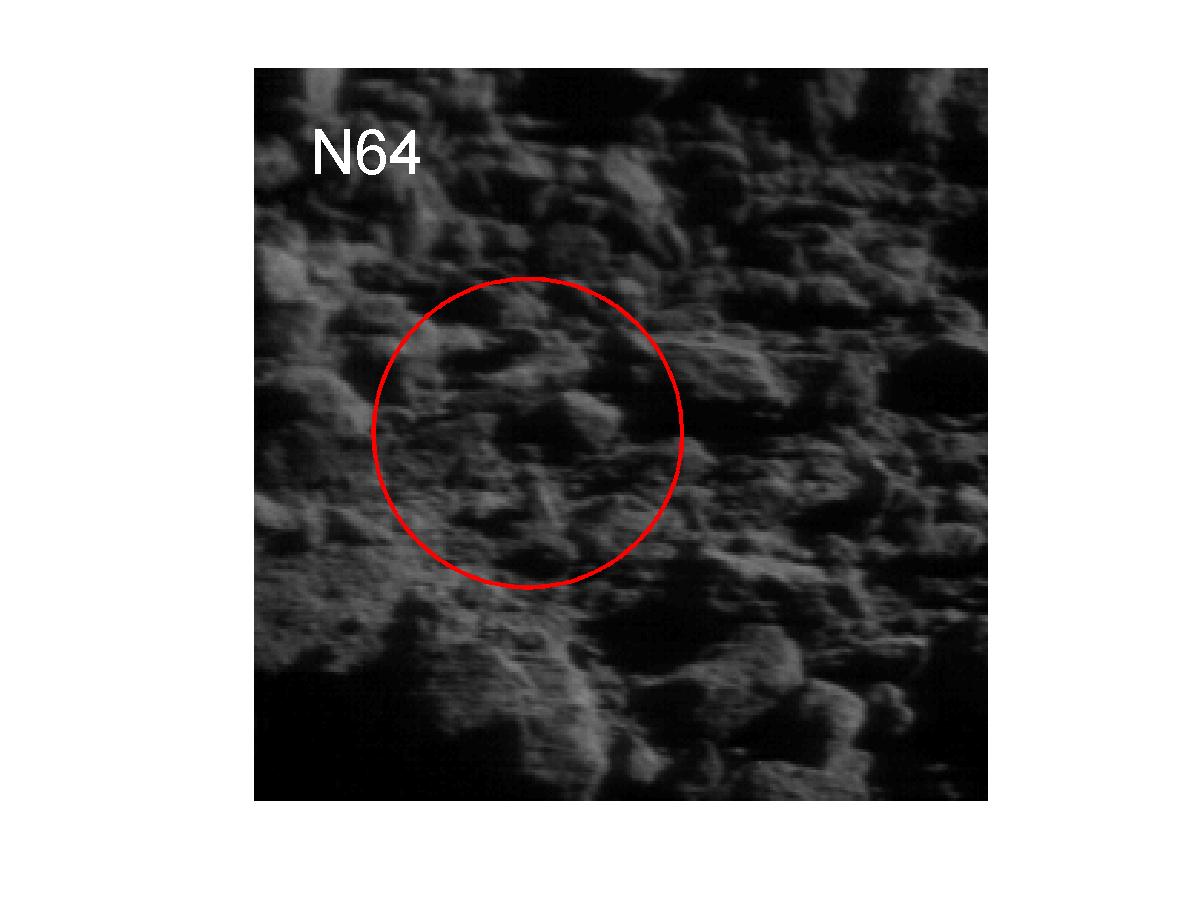

Supplement: Supplementary file 1 — Supplementary Information 1. [file 41598_2021_93694_MOESM1_ESM.zip › Supplementary_images of the spectral observations/N64.jpeg]

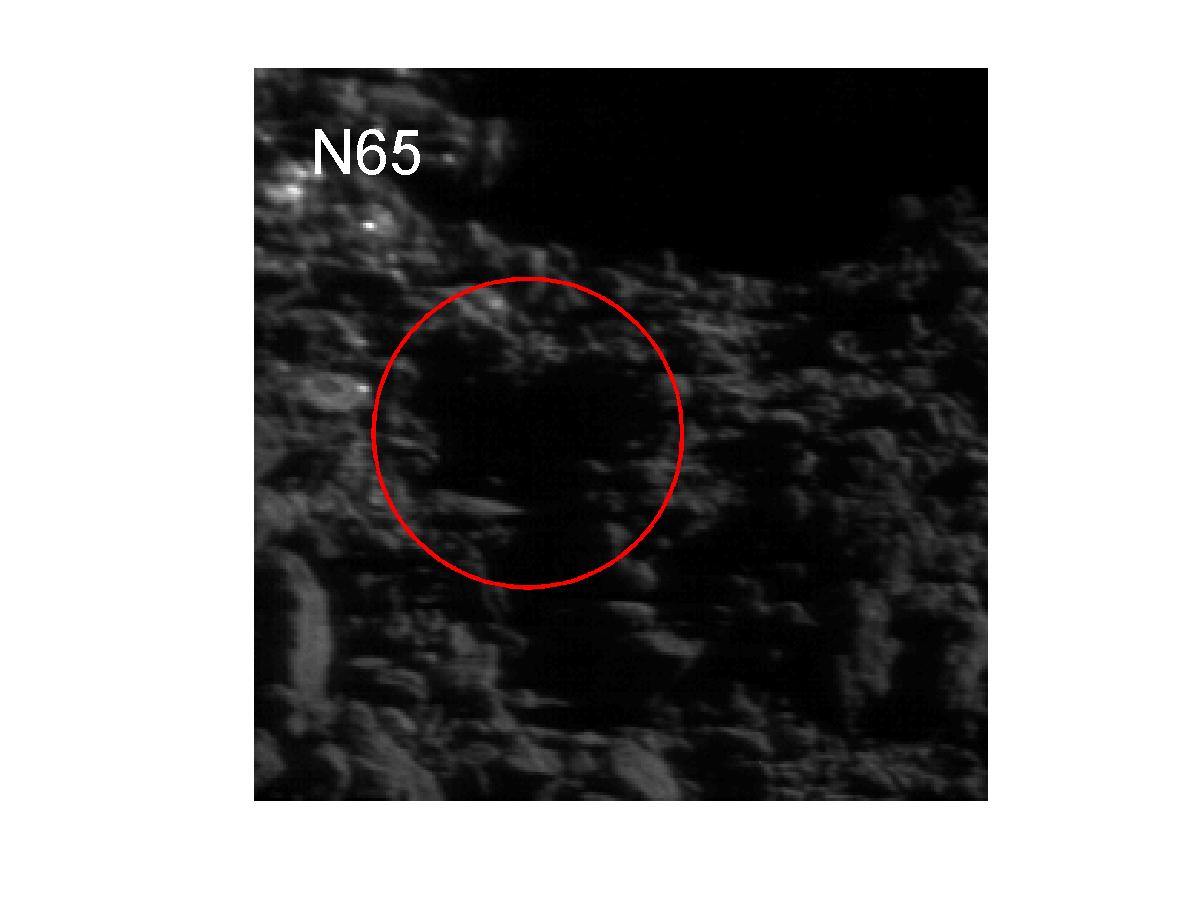

Supplement: Supplementary file 1 — Supplementary Information 1. [file 41598_2021_93694_MOESM1_ESM.zip › Supplementary_images of the spectral observations/N65.jpeg]

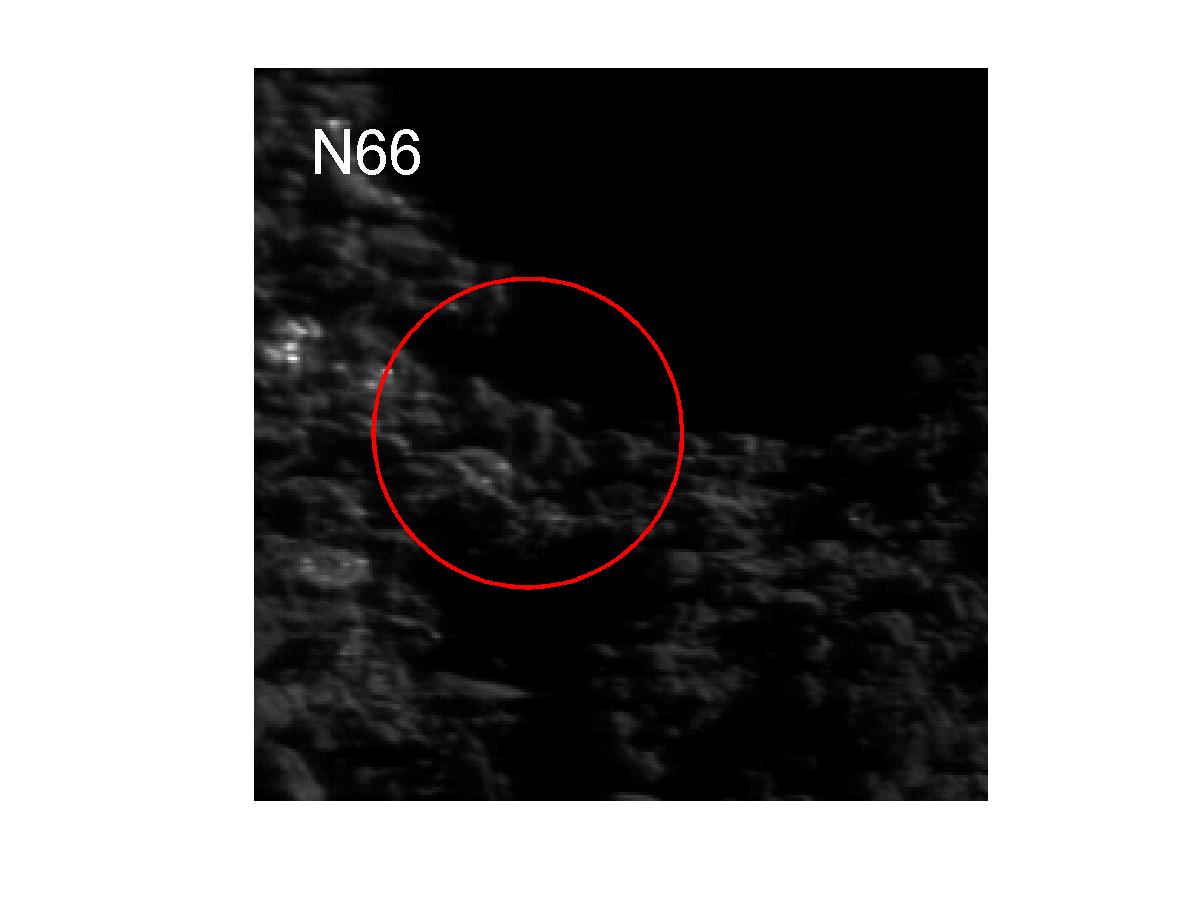

Supplement: Supplementary file 1 — Supplementary Information 1. [file 41598_2021_93694_MOESM1_ESM.zip › Supplementary_images of the spectral observations/N66.jpeg]

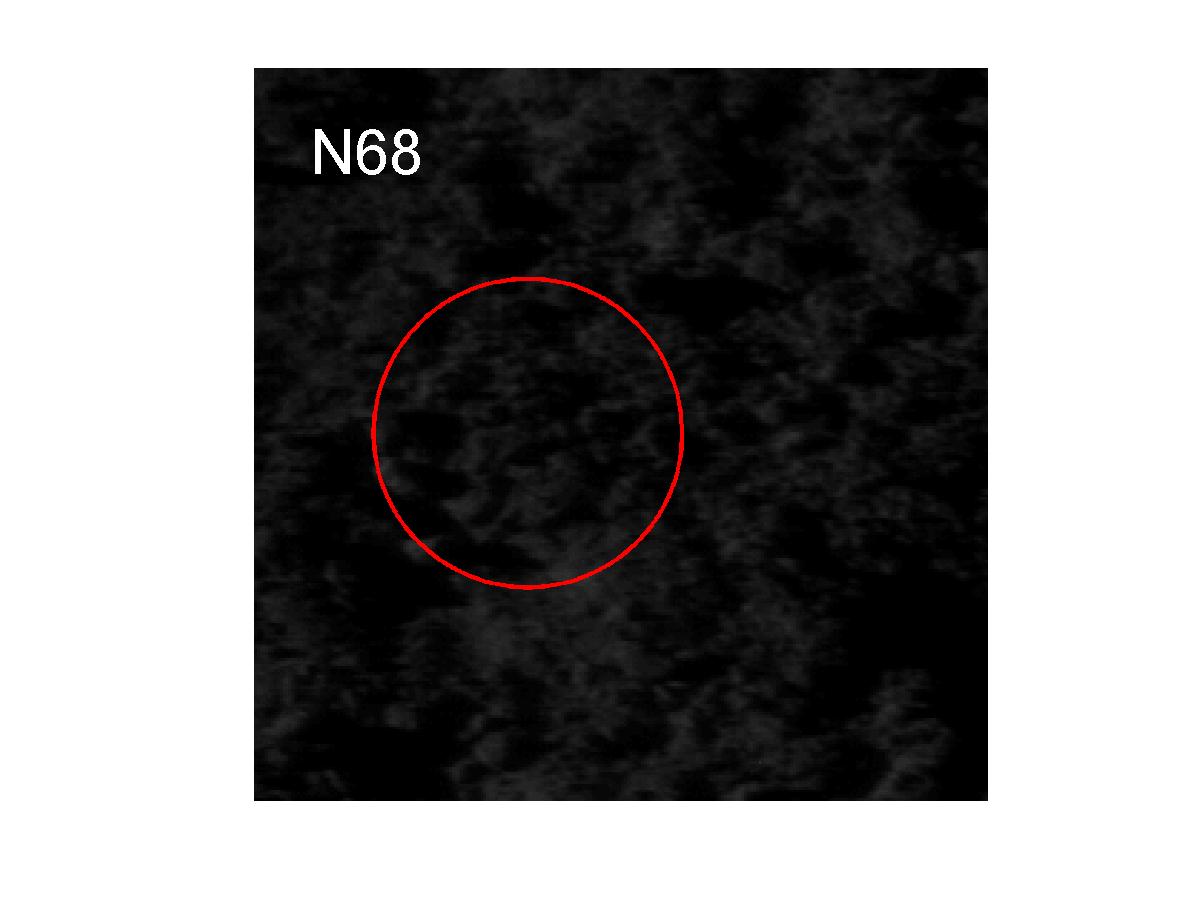

Supplement: Supplementary file 1 — Supplementary Information 1. [file 41598_2021_93694_MOESM1_ESM.zip › Supplementary_images of the spectral observations/N68.jpeg]

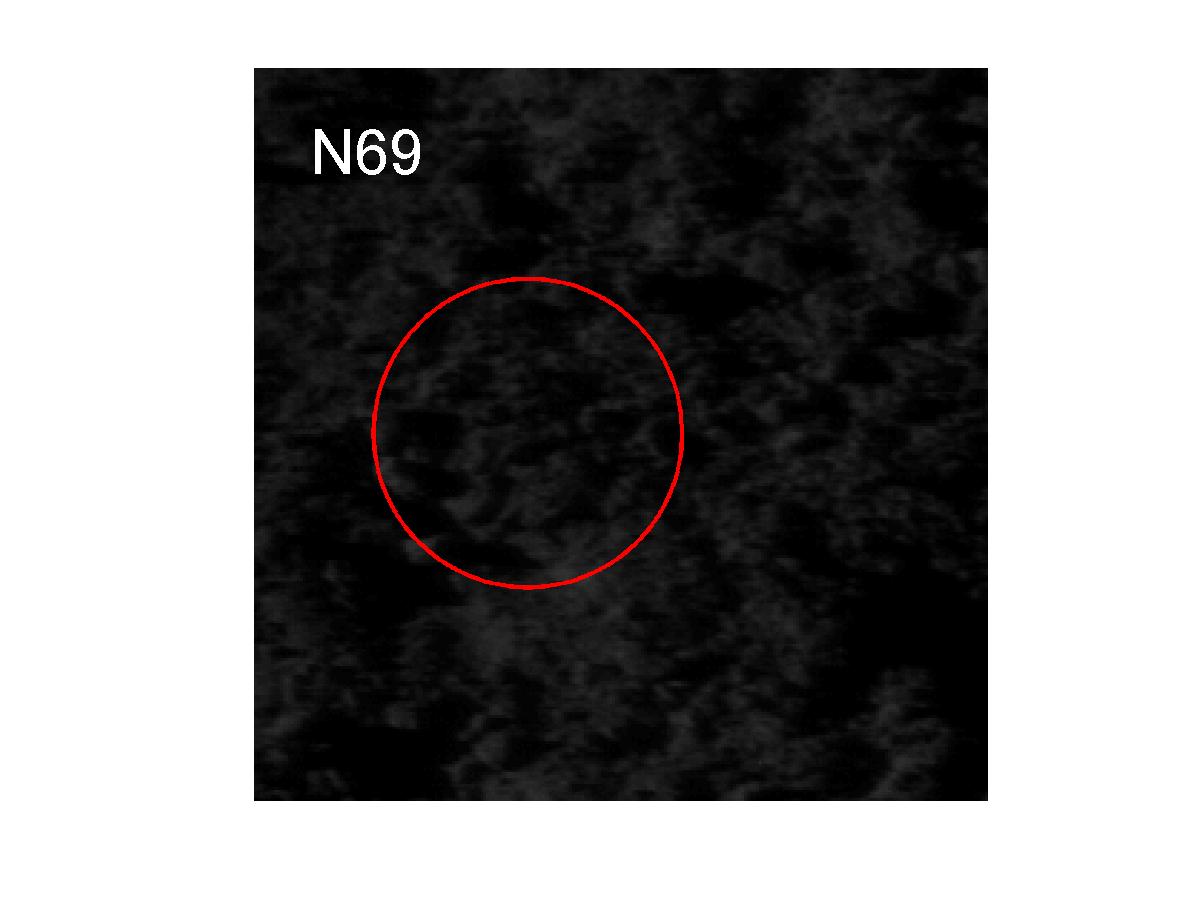

Supplement: Supplementary file 1 — Supplementary Information 1. [file 41598_2021_93694_MOESM1_ESM.zip › Supplementary_images of the spectral observations/N69.jpeg]

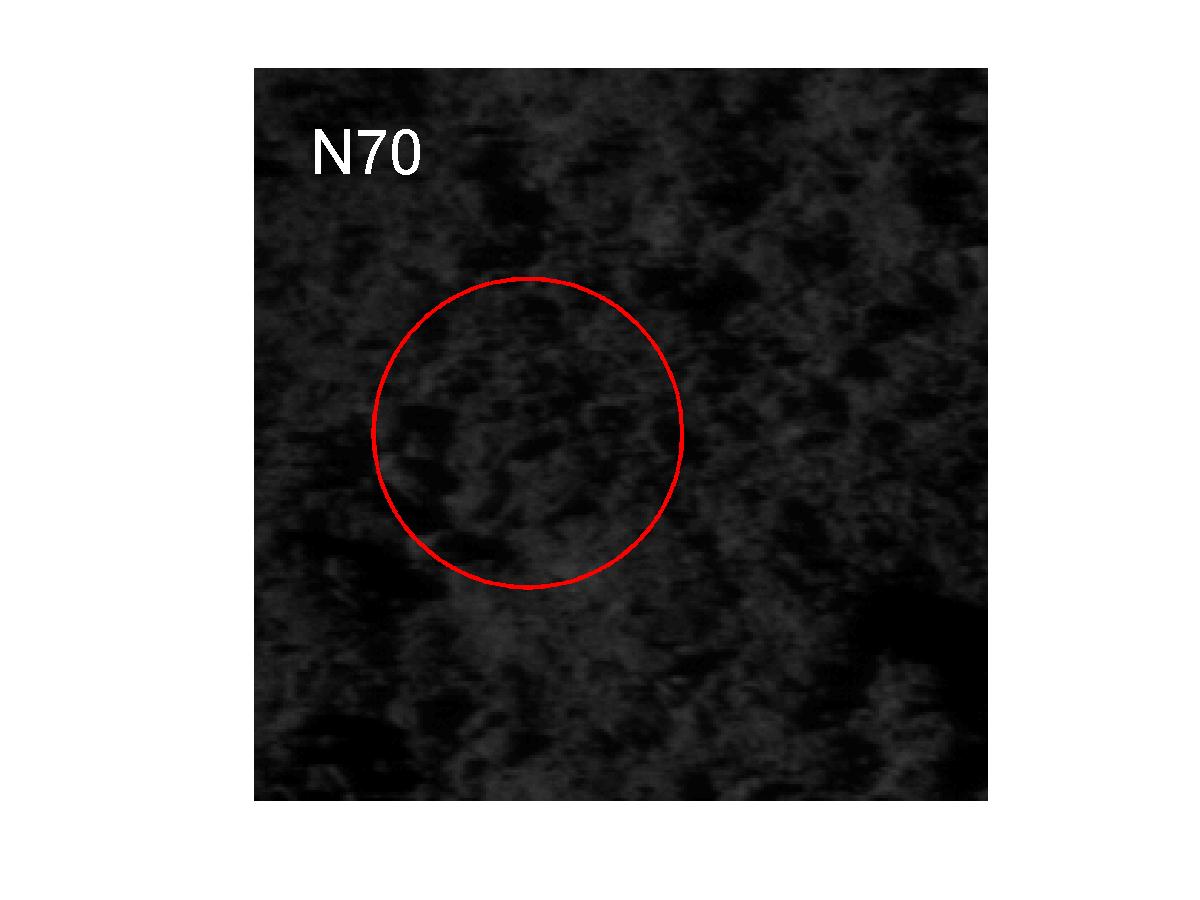

Supplement: Supplementary file 1 — Supplementary Information 1. [file 41598_2021_93694_MOESM1_ESM.zip › Supplementary_images of the spectral observations/N70.jpeg]

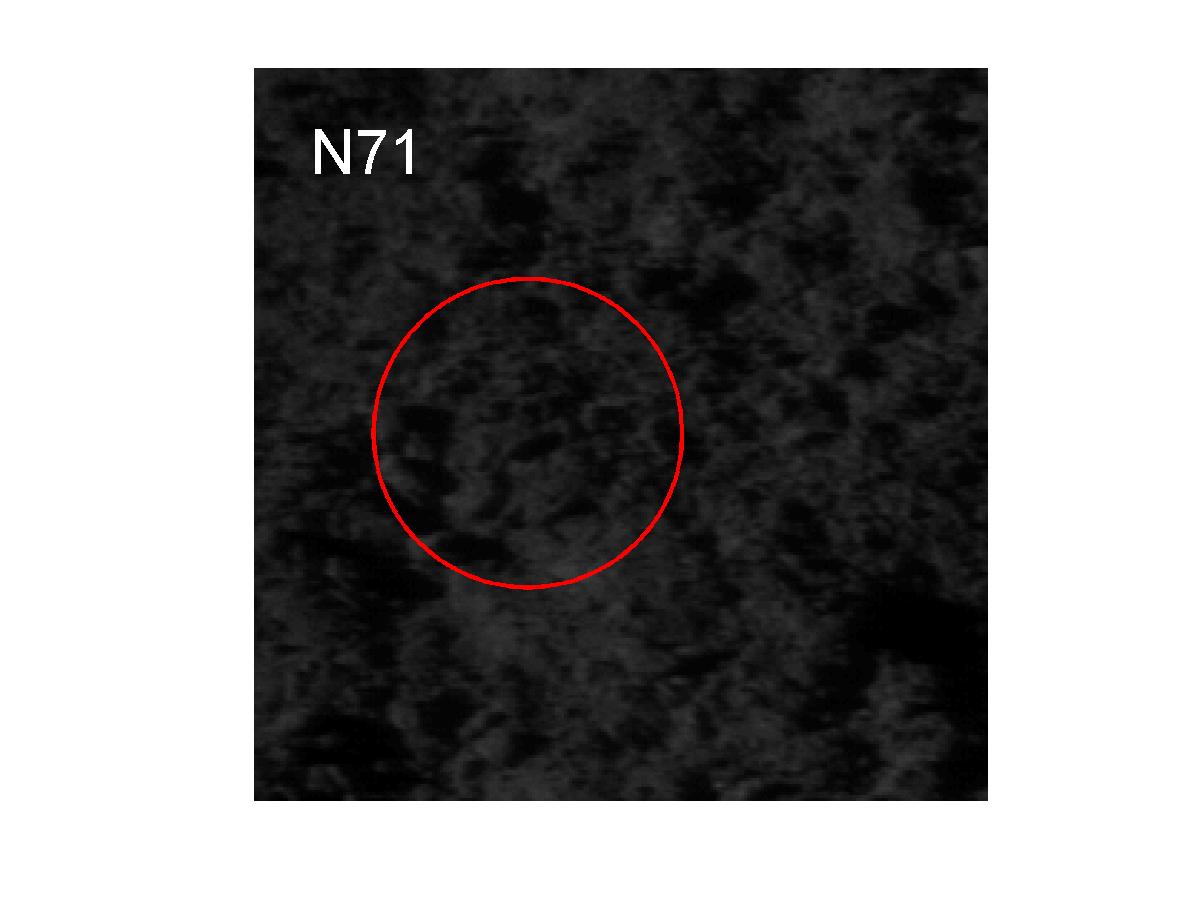

Supplement: Supplementary file 1 — Supplementary Information 1. [file 41598_2021_93694_MOESM1_ESM.zip › Supplementary_images of the spectral observations/N71.jpeg]

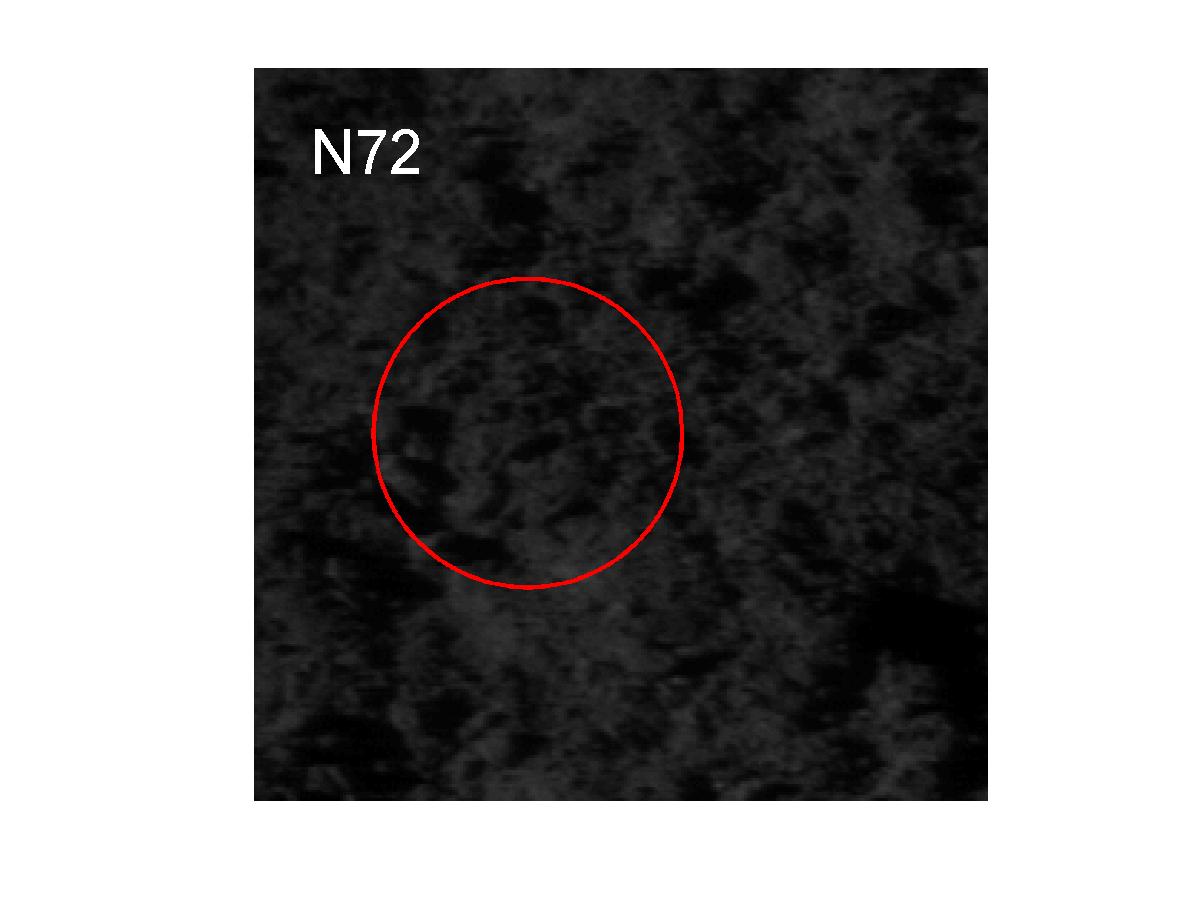

Supplement: Supplementary file 1 — Supplementary Information 1. [file 41598_2021_93694_MOESM1_ESM.zip › Supplementary_images of the spectral observations/N72.jpeg]

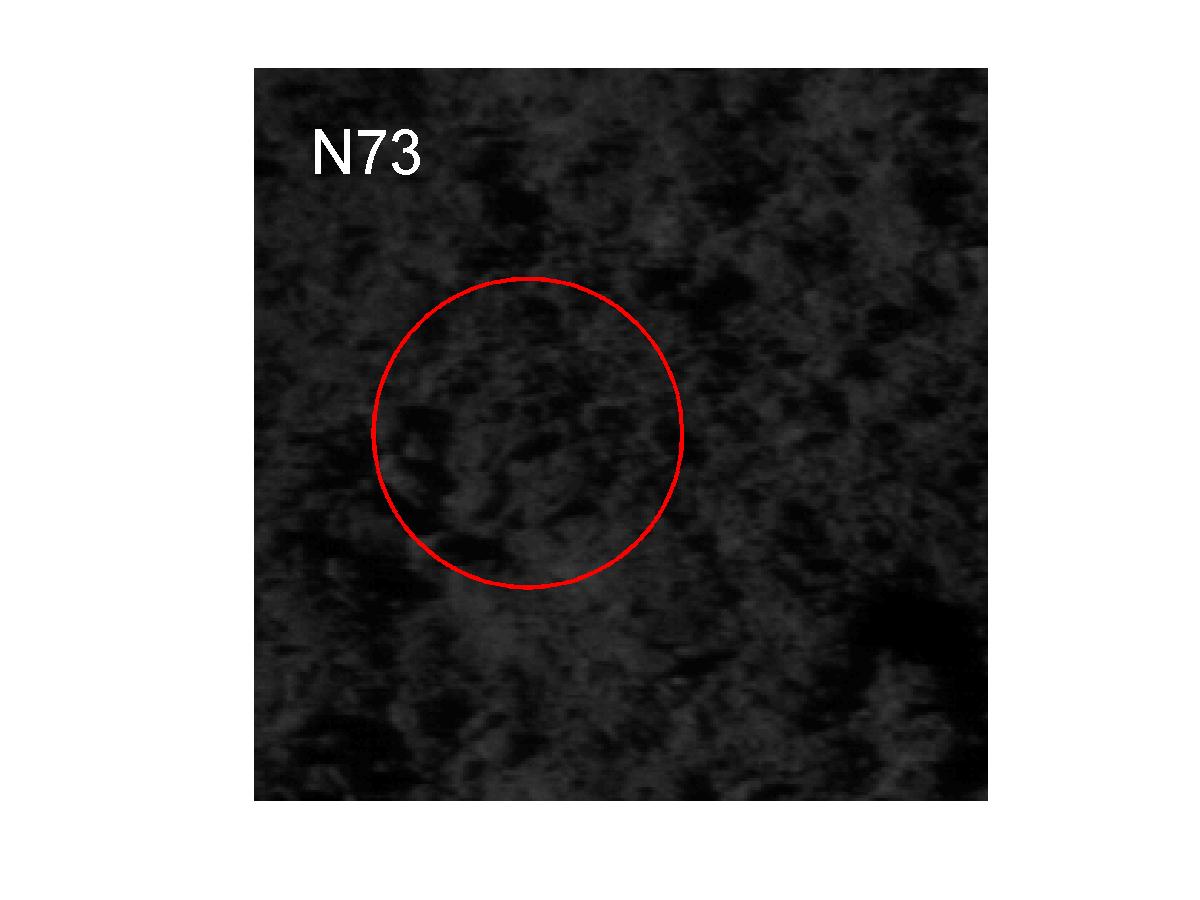

Supplement: Supplementary file 1 — Supplementary Information 1. [file 41598_2021_93694_MOESM1_ESM.zip › Supplementary_images of the spectral observations/N73.jpeg]

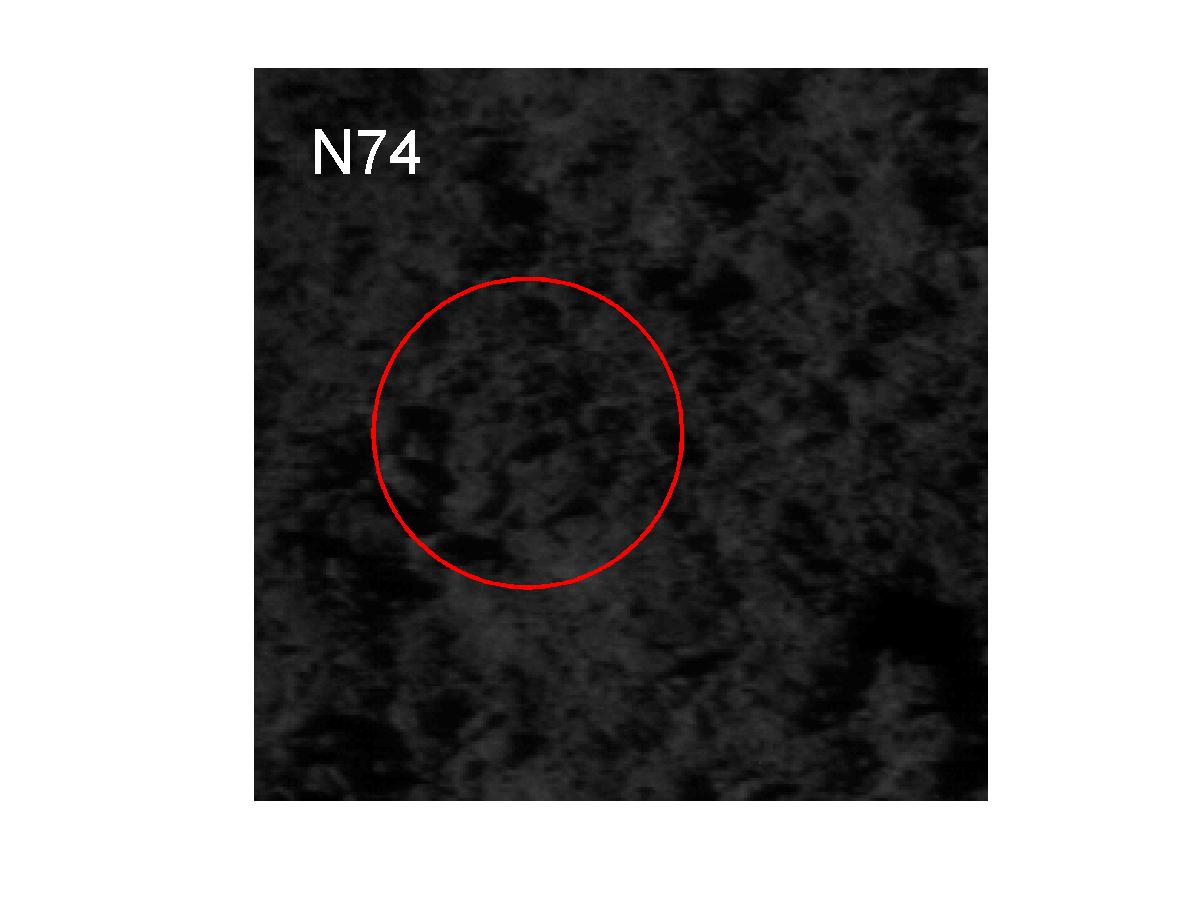

Supplement: Supplementary file 1 — Supplementary Information 1. [file 41598_2021_93694_MOESM1_ESM.zip › Supplementary_images of the spectral observations/N74.jpeg]

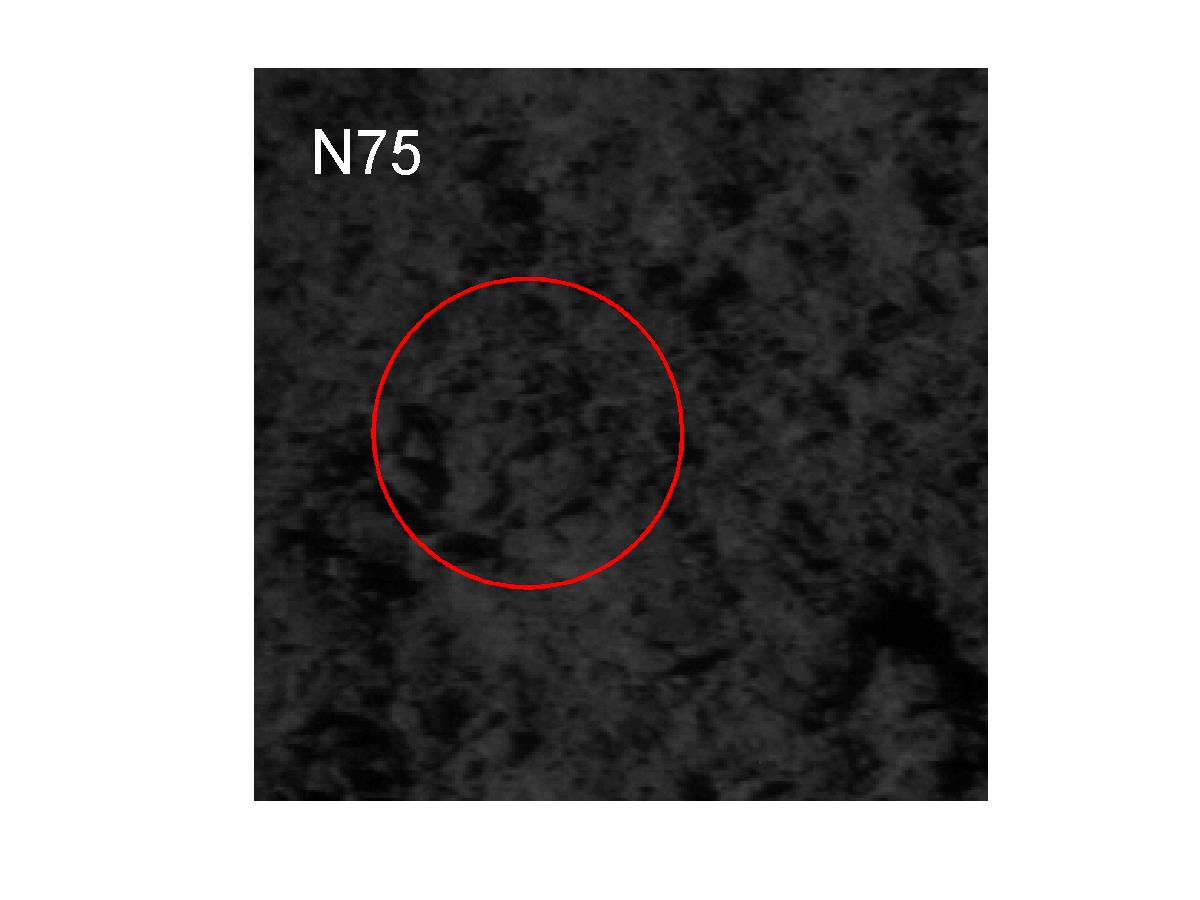

Supplement: Supplementary file 1 — Supplementary Information 1. [file 41598_2021_93694_MOESM1_ESM.zip › Supplementary_images of the spectral observations/N75.jpeg]

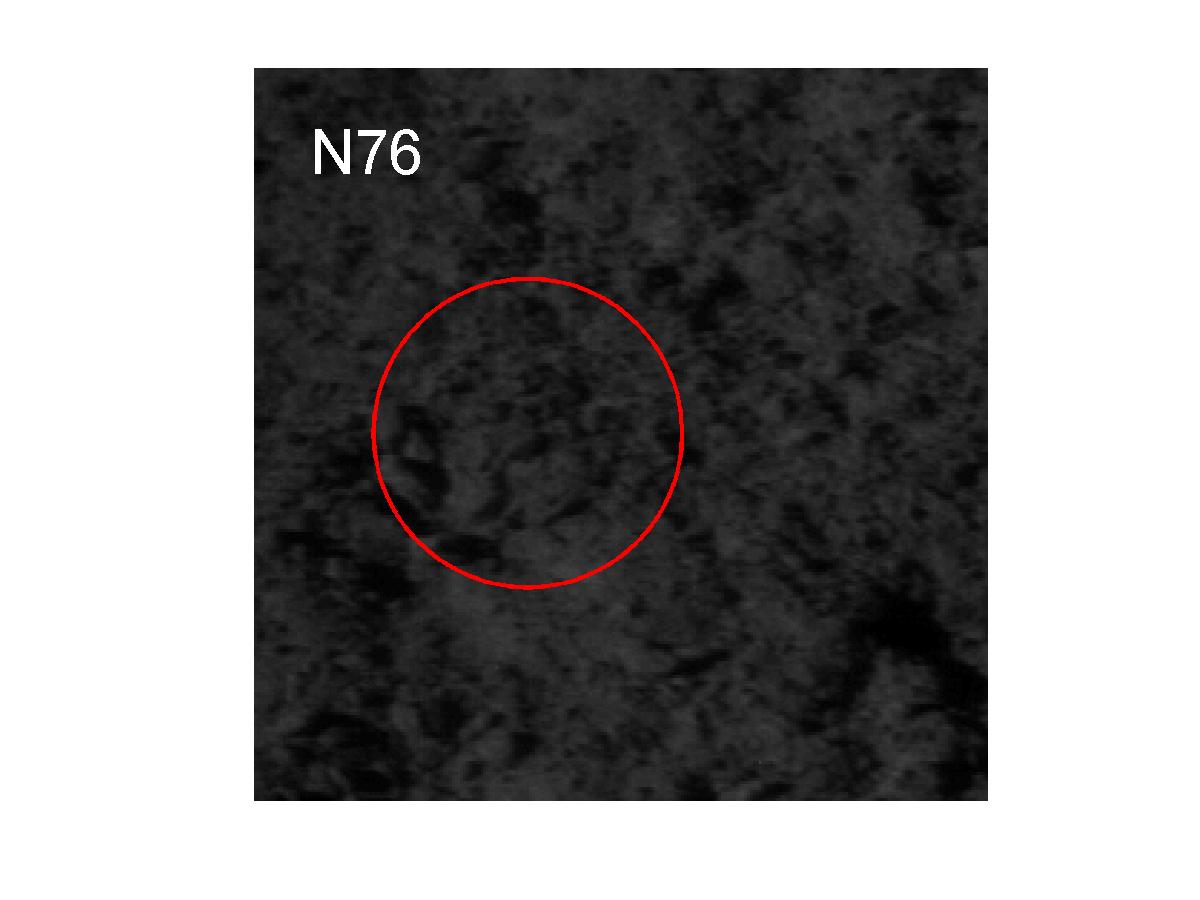

Supplement: Supplementary file 1 — Supplementary Information 1. [file 41598_2021_93694_MOESM1_ESM.zip › Supplementary_images of the spectral observations/N76.jpeg]

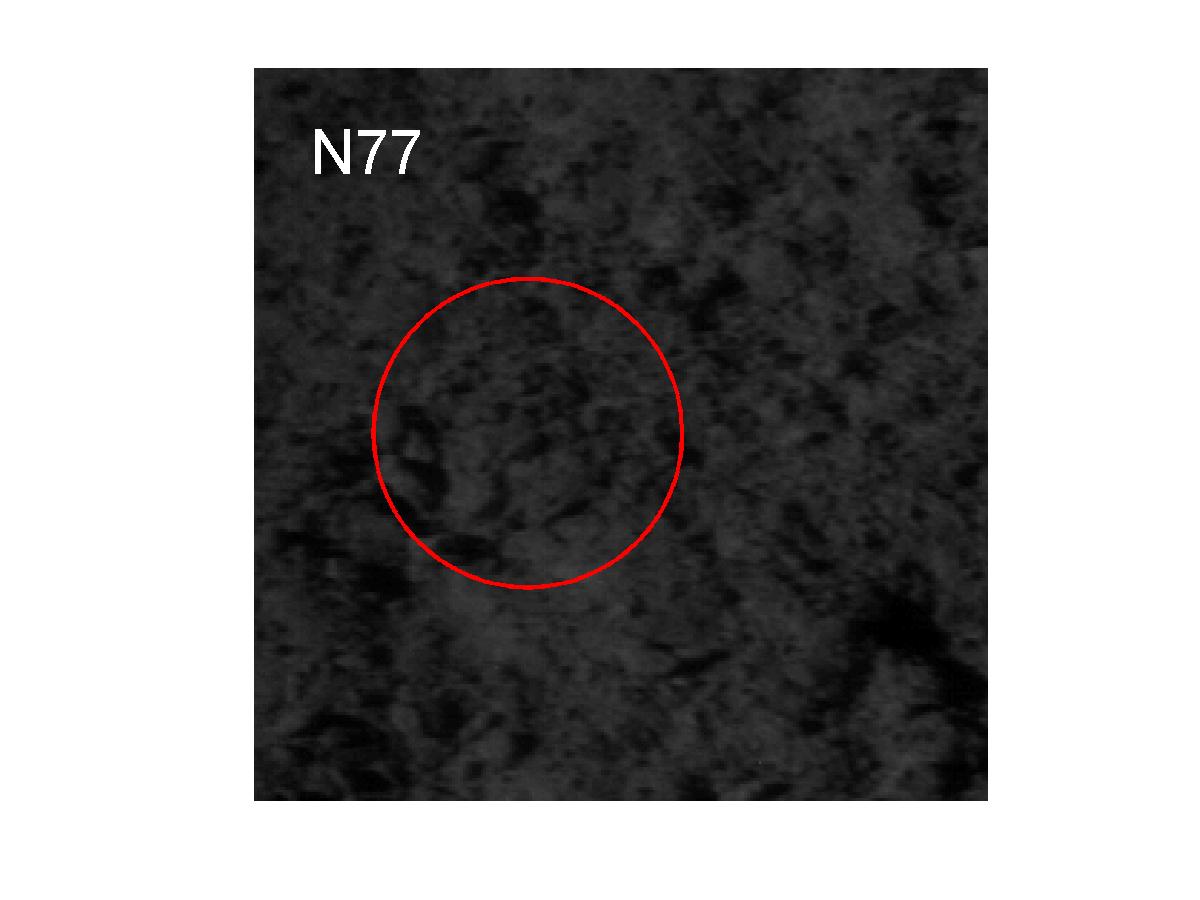

Supplement: Supplementary file 1 — Supplementary Information 1. [file 41598_2021_93694_MOESM1_ESM.zip › Supplementary_images of the spectral observations/N77.jpeg]

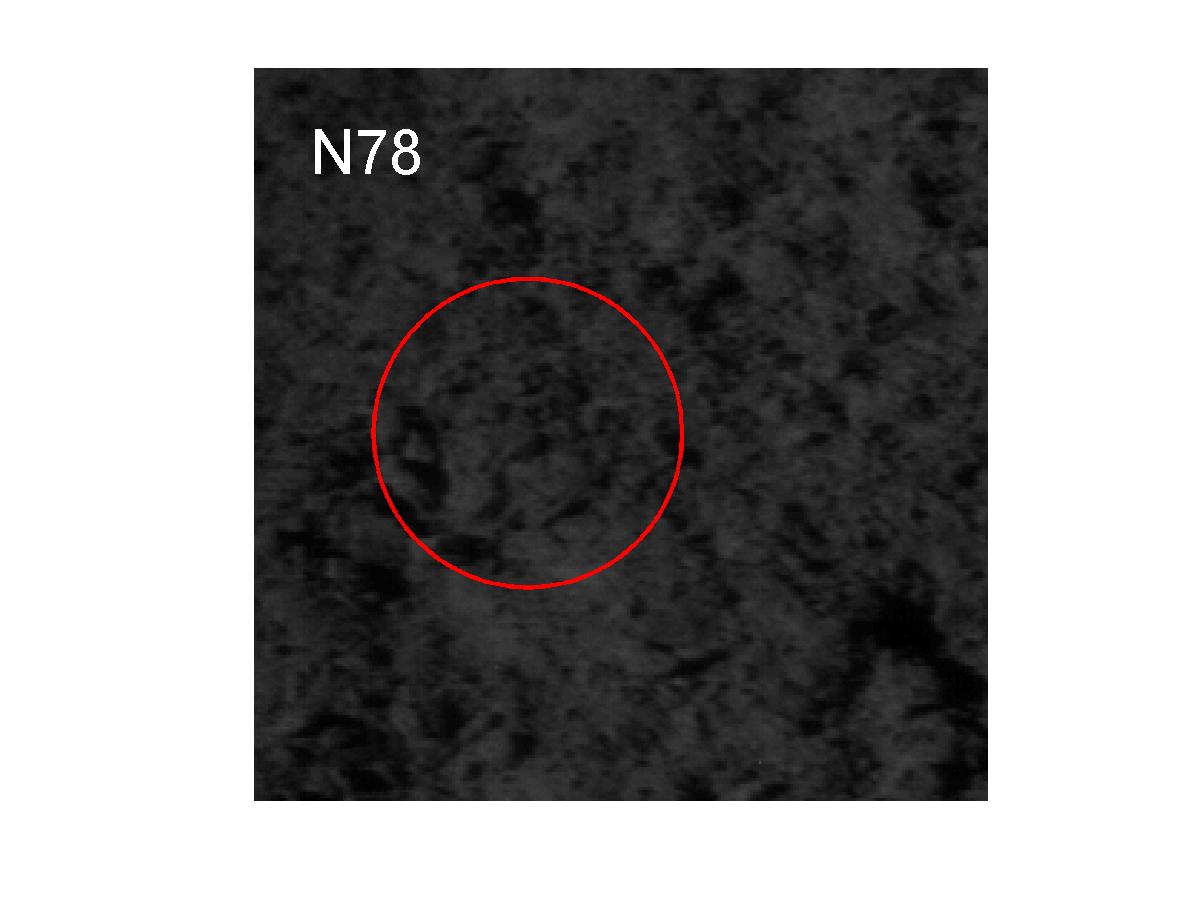

Supplement: Supplementary file 1 — Supplementary Information 1. [file 41598_2021_93694_MOESM1_ESM.zip › Supplementary_images of the spectral observations/N78.jpeg]

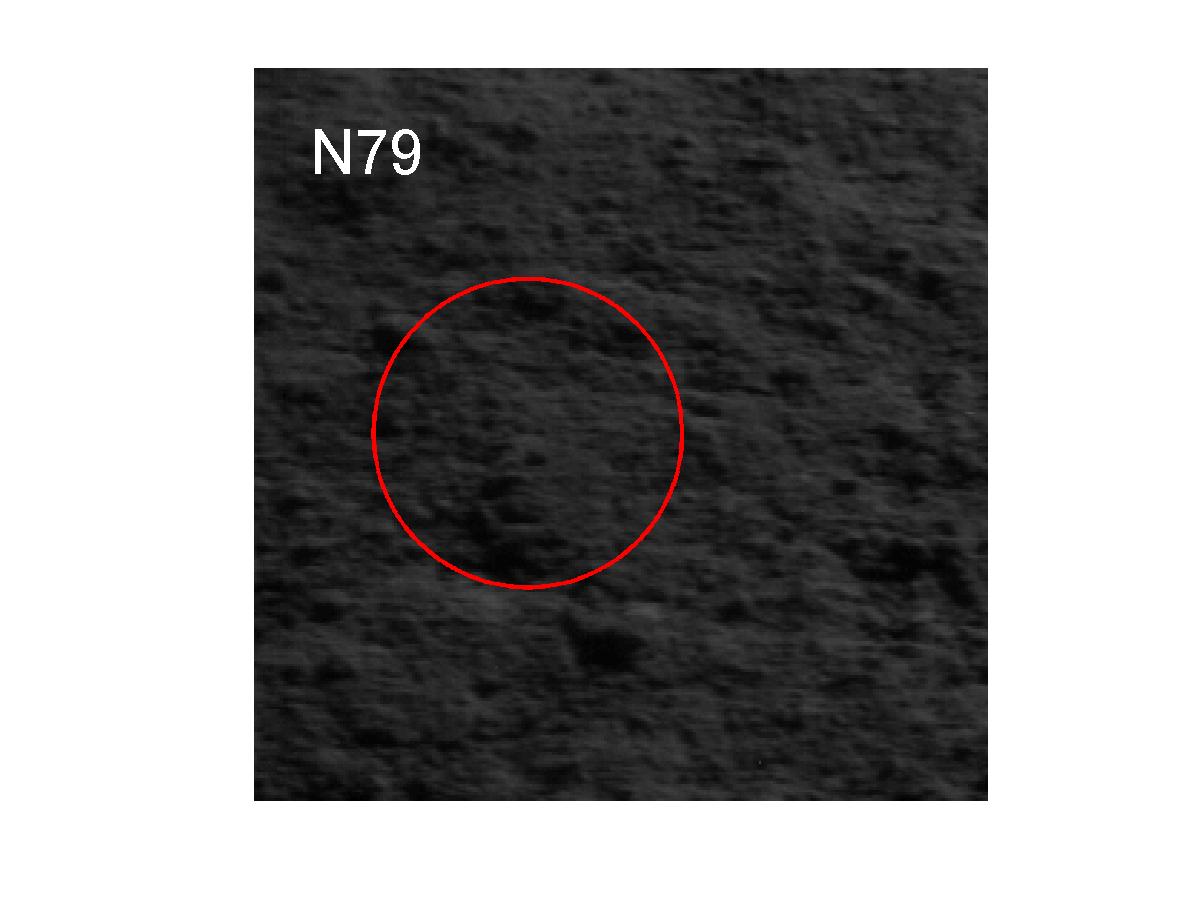

Supplement: Supplementary file 1 — Supplementary Information 1. [file 41598_2021_93694_MOESM1_ESM.zip › Supplementary_images of the spectral observations/N79.jpeg]

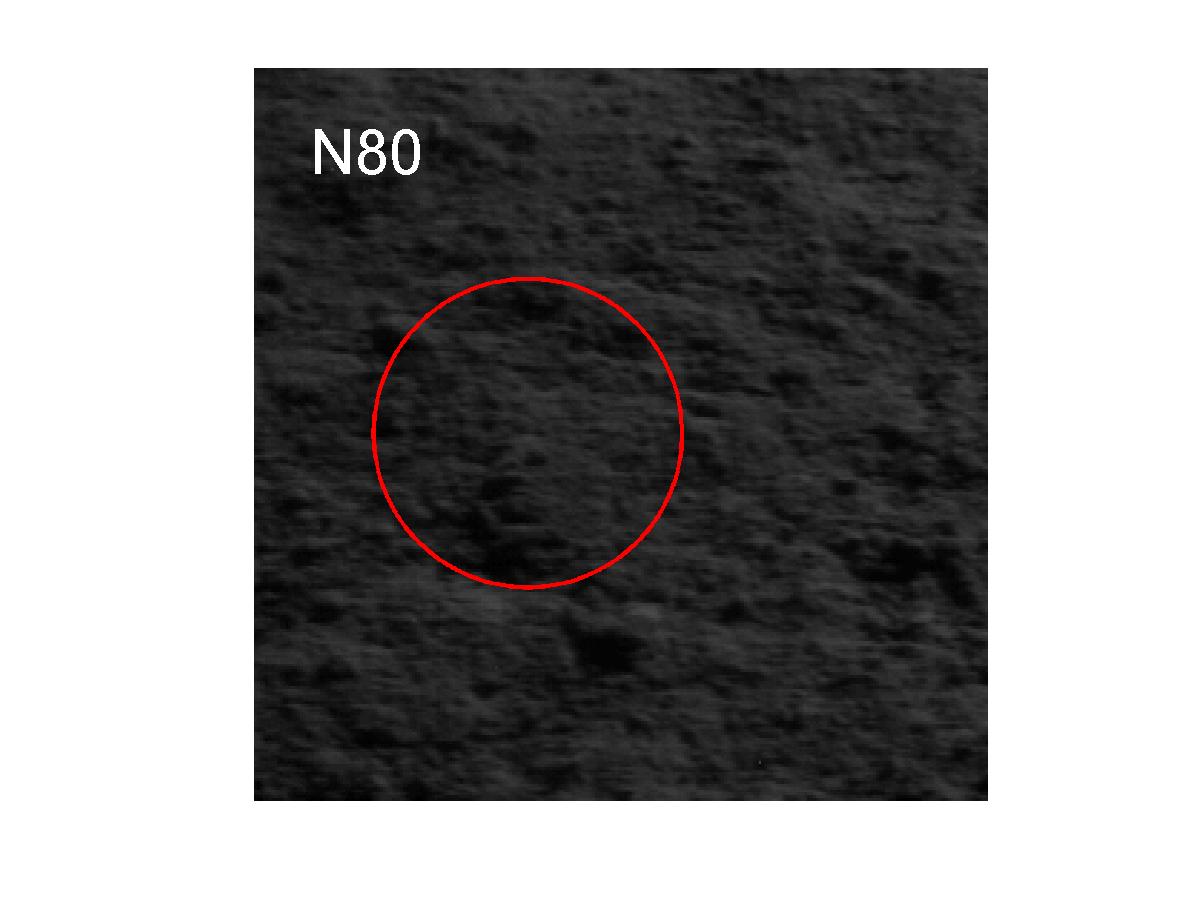

Supplement: Supplementary file 1 — Supplementary Information 1. [file 41598_2021_93694_MOESM1_ESM.zip › Supplementary_images of the spectral observations/N80.jpeg]

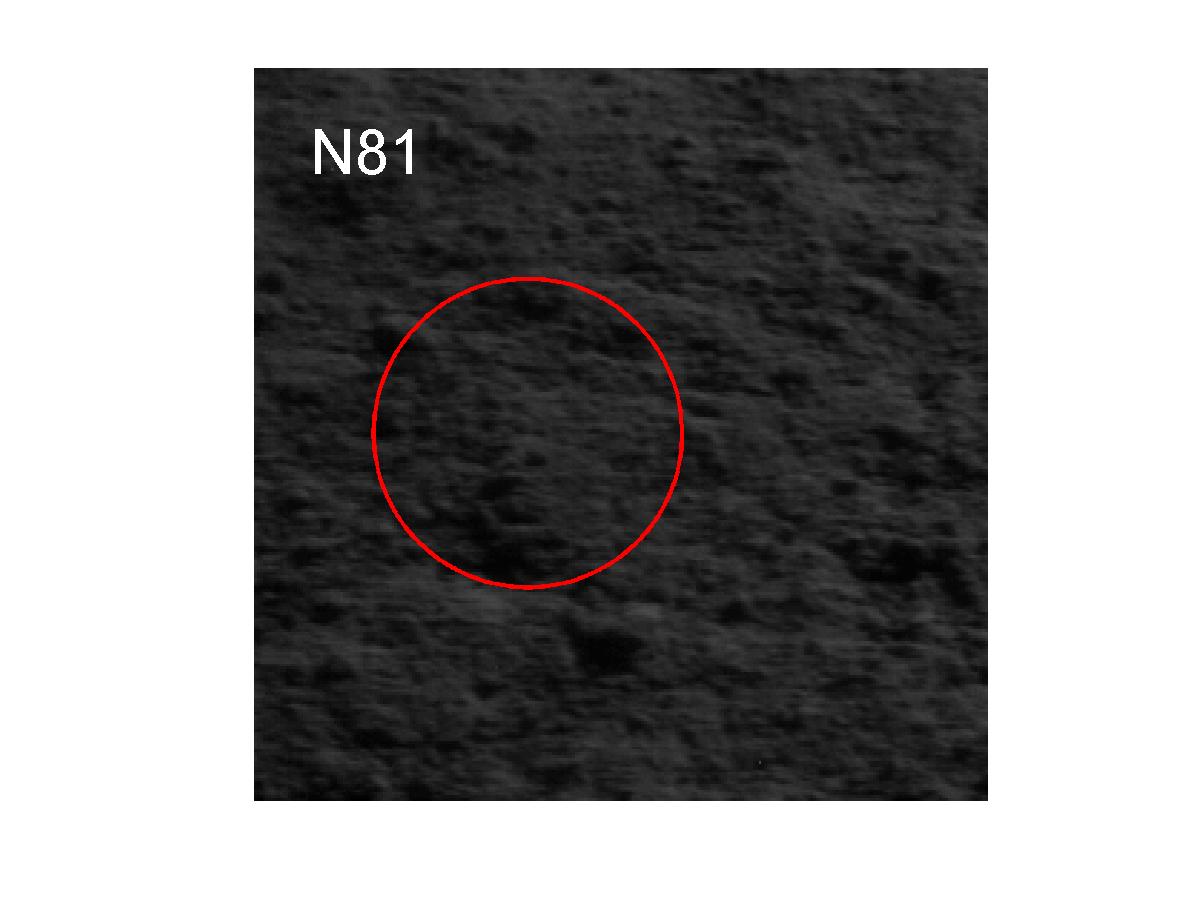

Supplement: Supplementary file 1 — Supplementary Information 1. [file 41598_2021_93694_MOESM1_ESM.zip › Supplementary_images of the spectral observations/N81.jpeg]

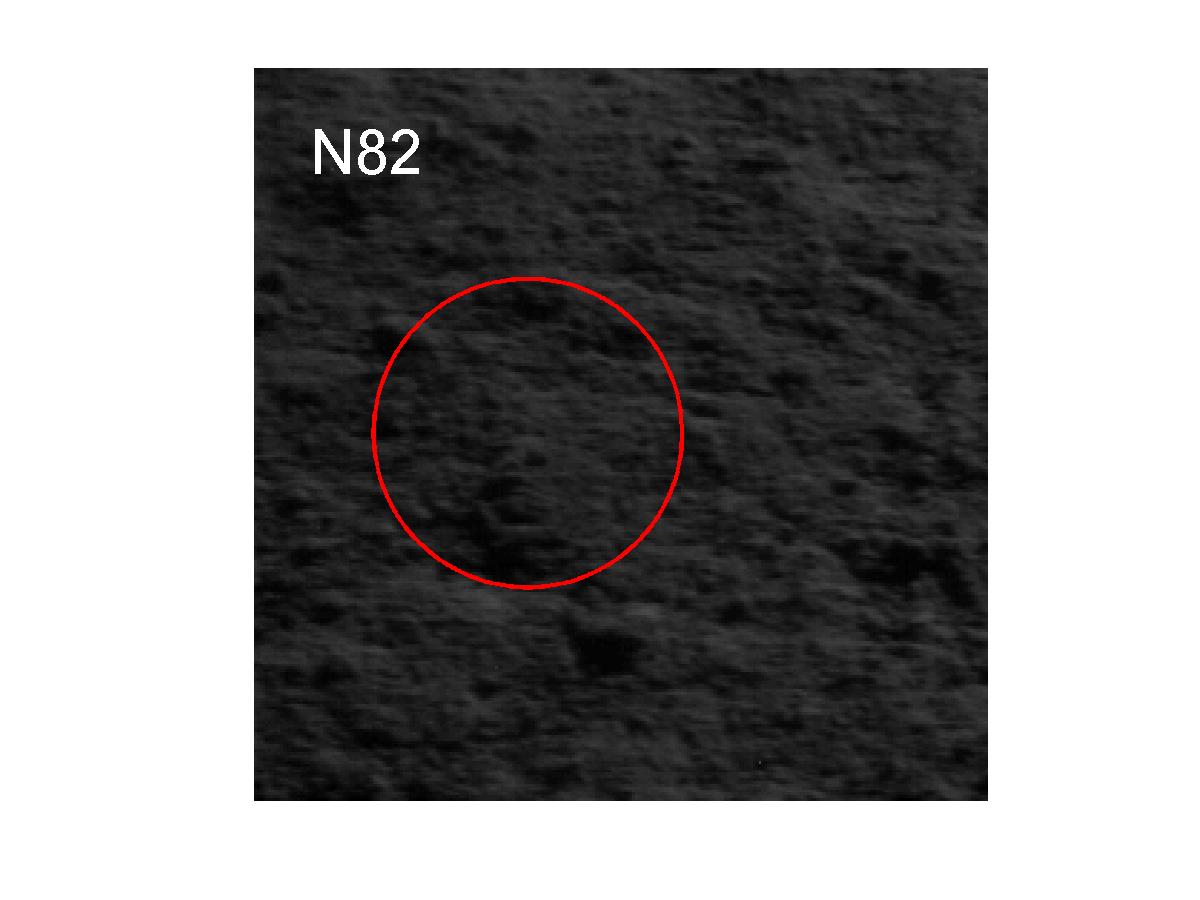

Supplement: Supplementary file 1 — Supplementary Information 1. [file 41598_2021_93694_MOESM1_ESM.zip › Supplementary_images of the spectral observations/N82.jpeg]

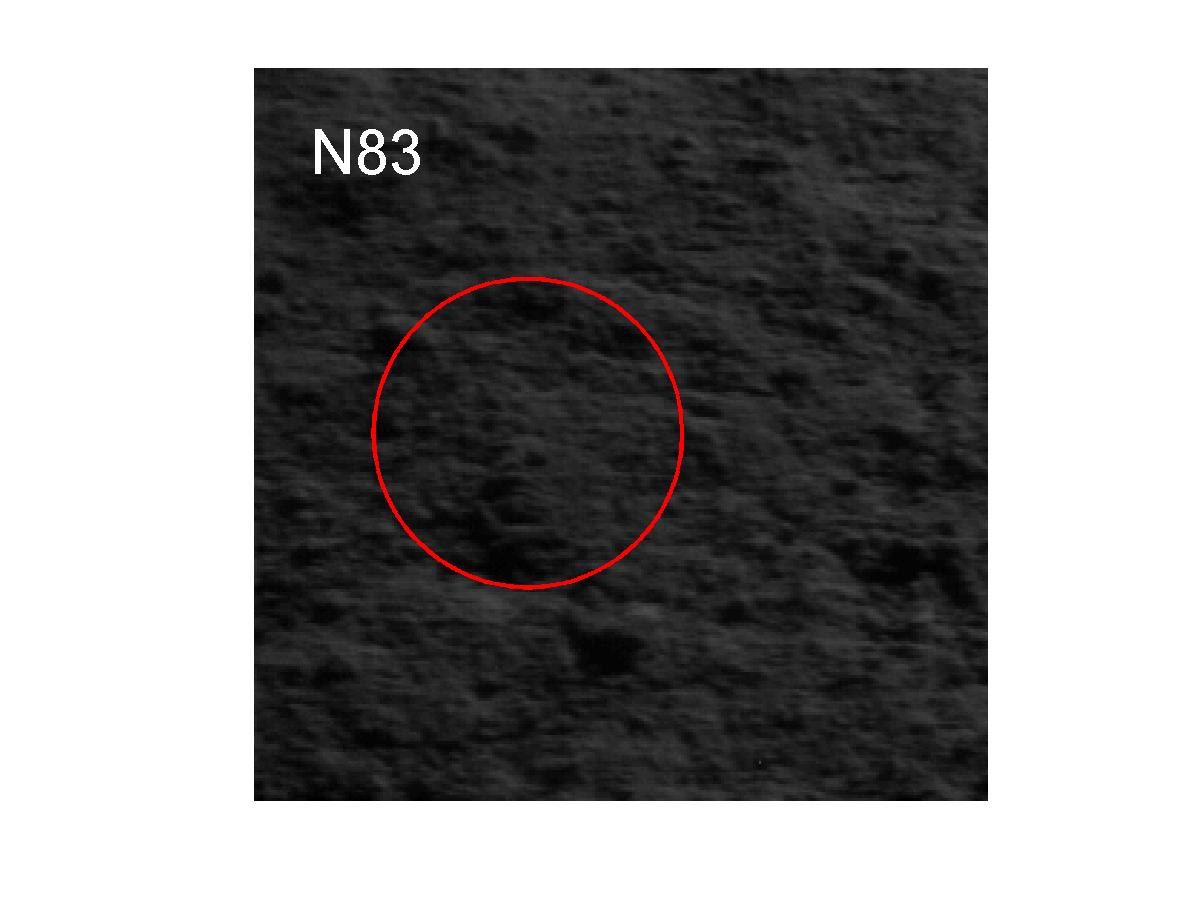

Supplement: Supplementary file 1 — Supplementary Information 1. [file 41598_2021_93694_MOESM1_ESM.zip › Supplementary_images of the spectral observations/N83.jpeg]

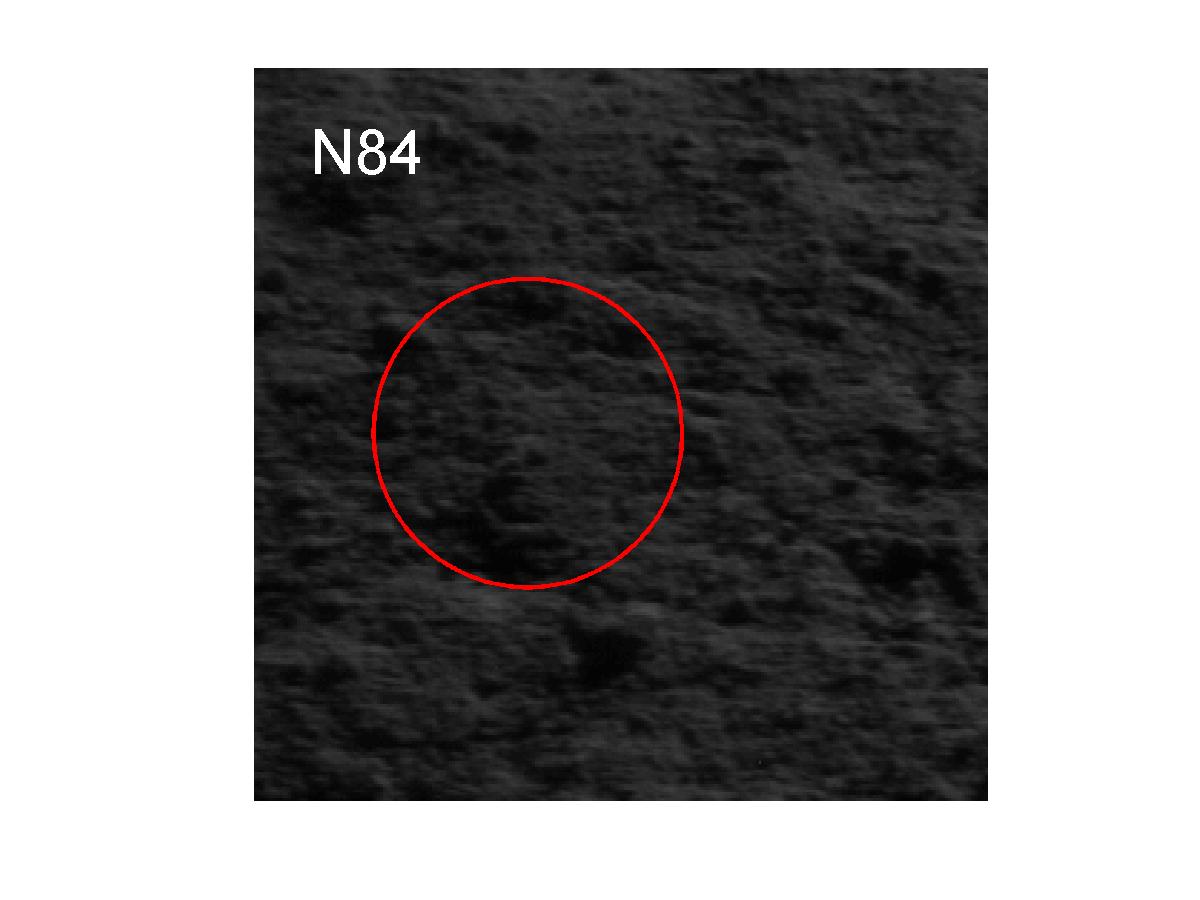

Supplement: Supplementary file 1 — Supplementary Information 1. [file 41598_2021_93694_MOESM1_ESM.zip › Supplementary_images of the spectral observations/N84.jpeg]

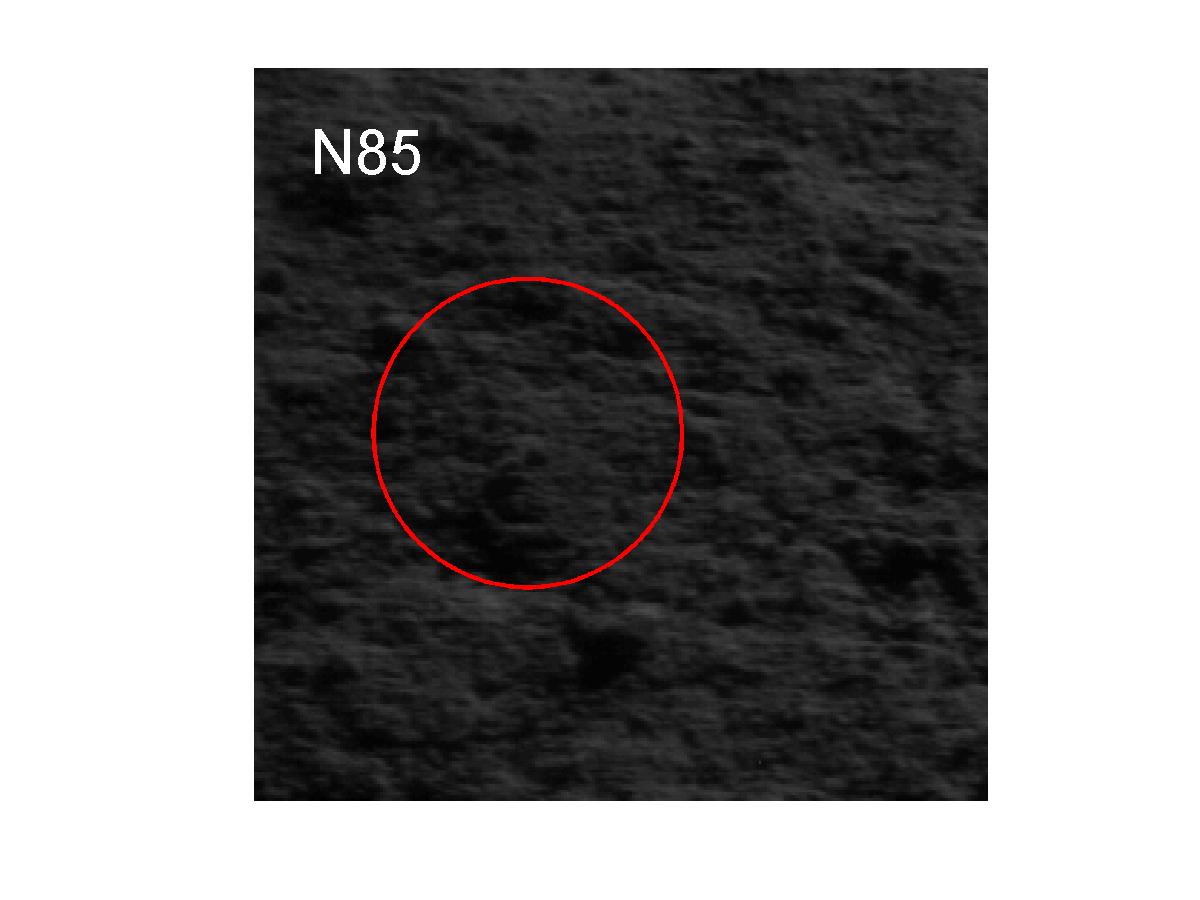

Supplement: Supplementary file 1 — Supplementary Information 1. [file 41598_2021_93694_MOESM1_ESM.zip › Supplementary_images of the spectral observations/N85.jpeg]

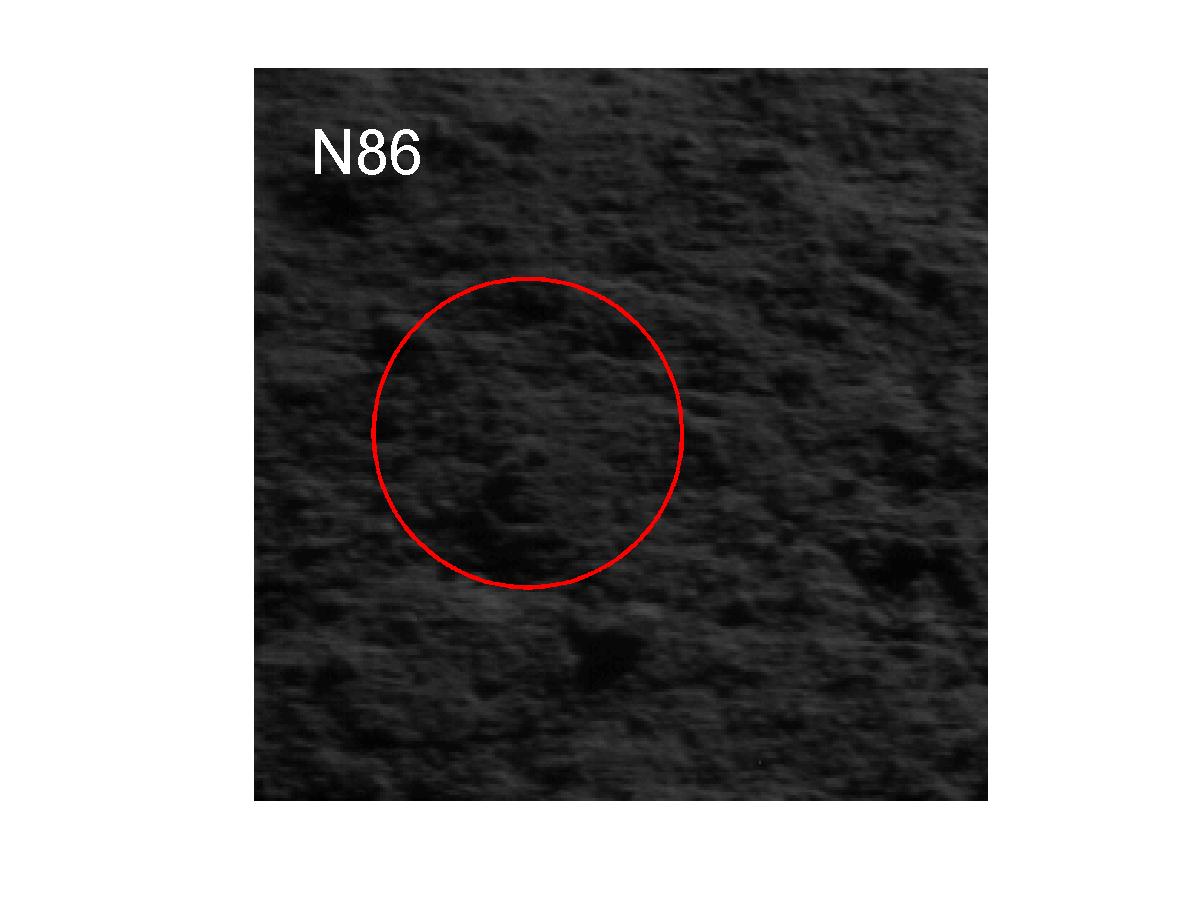

Supplement: Supplementary file 1 — Supplementary Information 1. [file 41598_2021_93694_MOESM1_ESM.zip › Supplementary_images of the spectral observations/N86.jpeg]

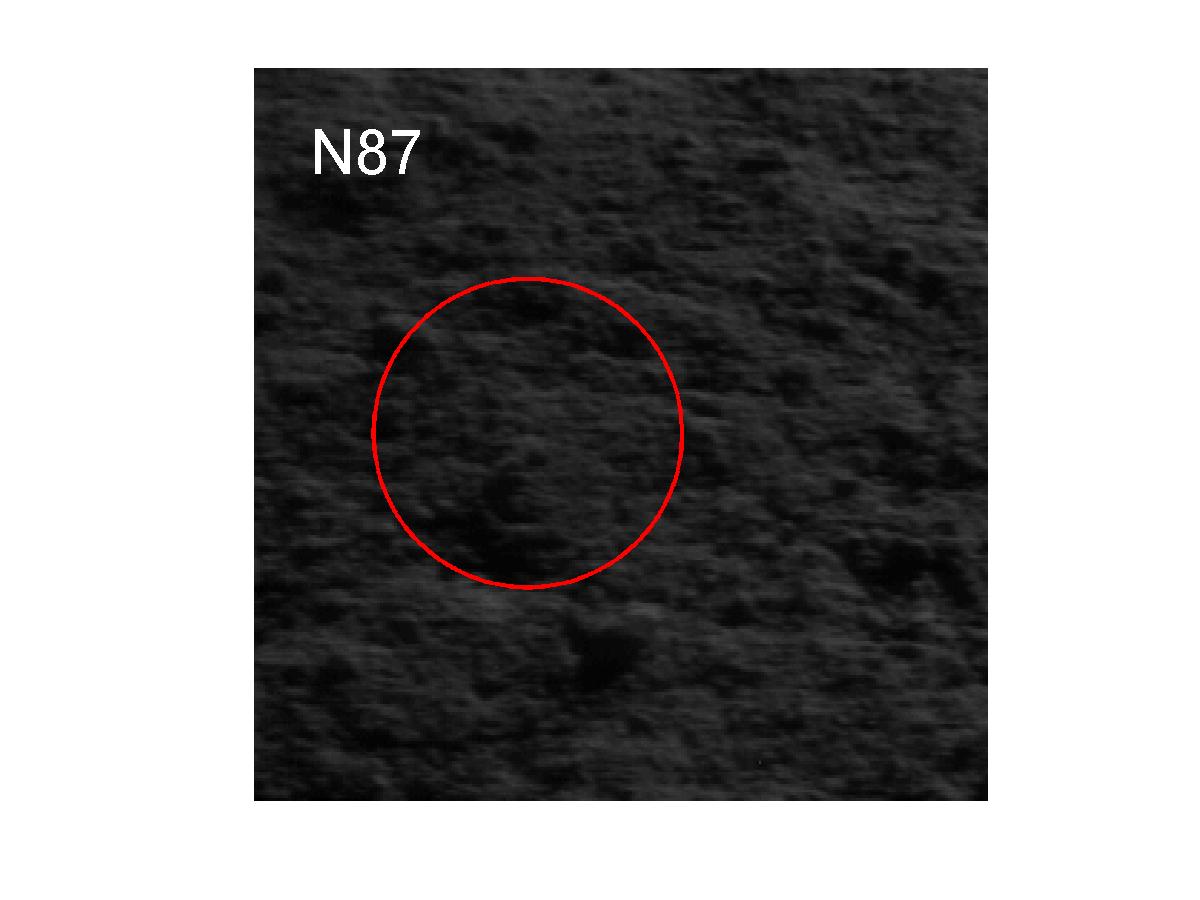

Supplement: Supplementary file 1 — Supplementary Information 1. [file 41598_2021_93694_MOESM1_ESM.zip › Supplementary_images of the spectral observations/N87.jpeg]

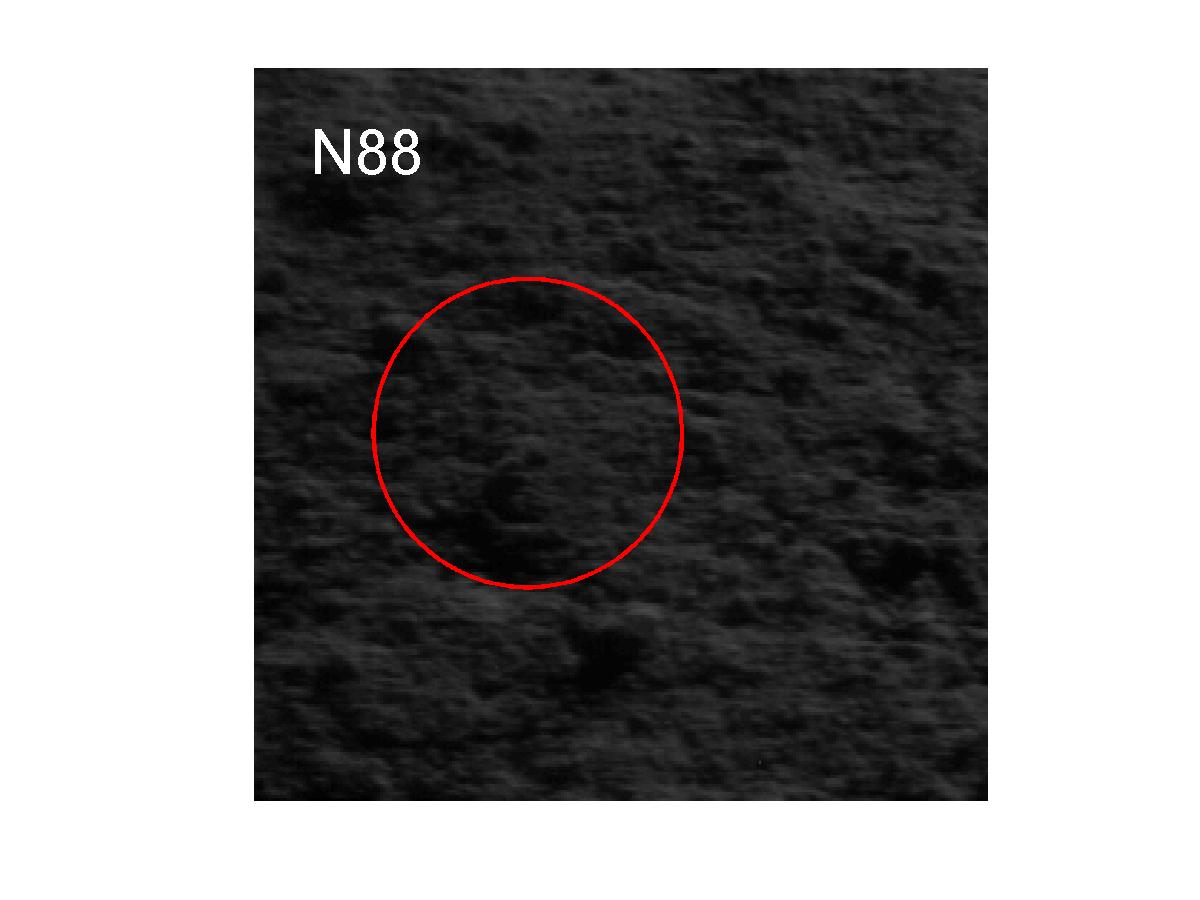

Supplement: Supplementary file 1 — Supplementary Information 1. [file 41598_2021_93694_MOESM1_ESM.zip › Supplementary_images of the spectral observations/N88.jpeg]

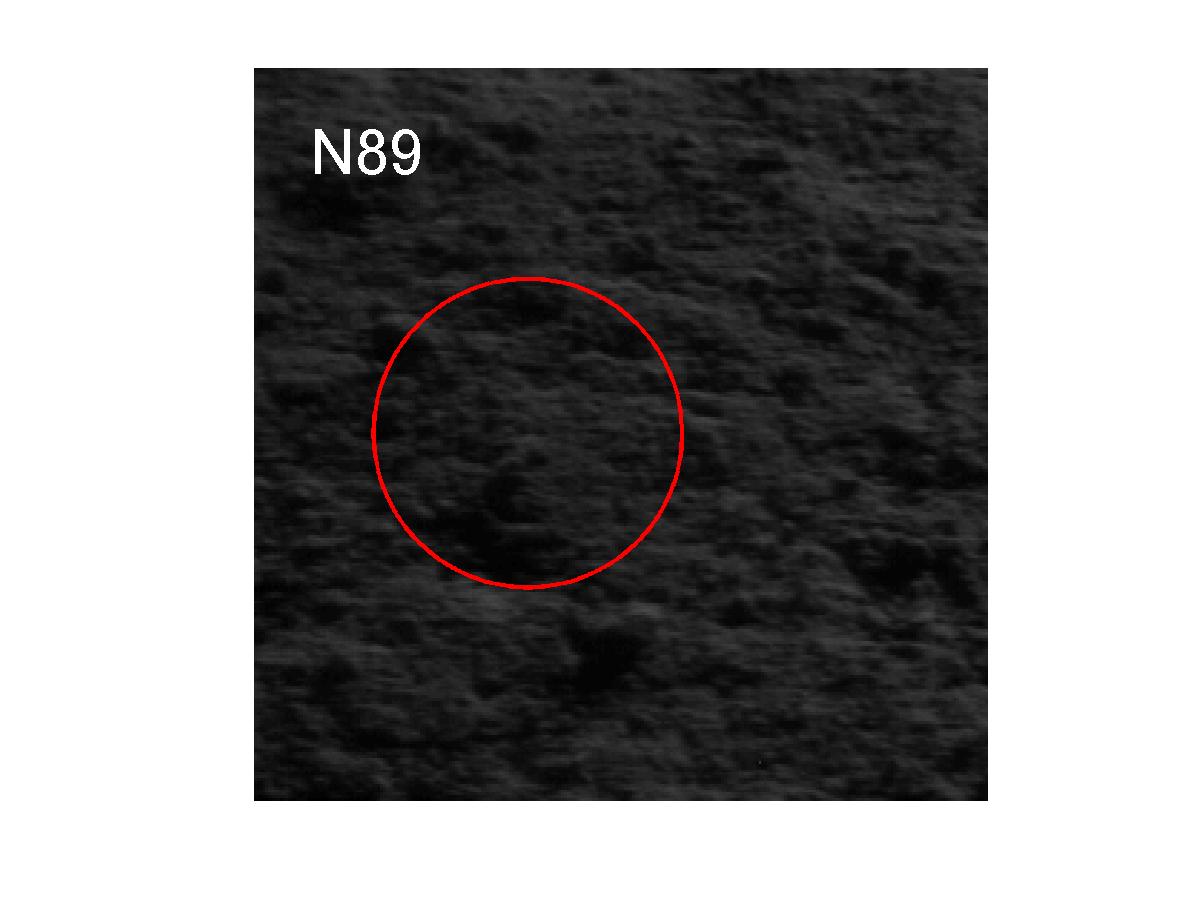

Supplement: Supplementary file 1 — Supplementary Information 1. [file 41598_2021_93694_MOESM1_ESM.zip › Supplementary_images of the spectral observations/N89.jpeg]

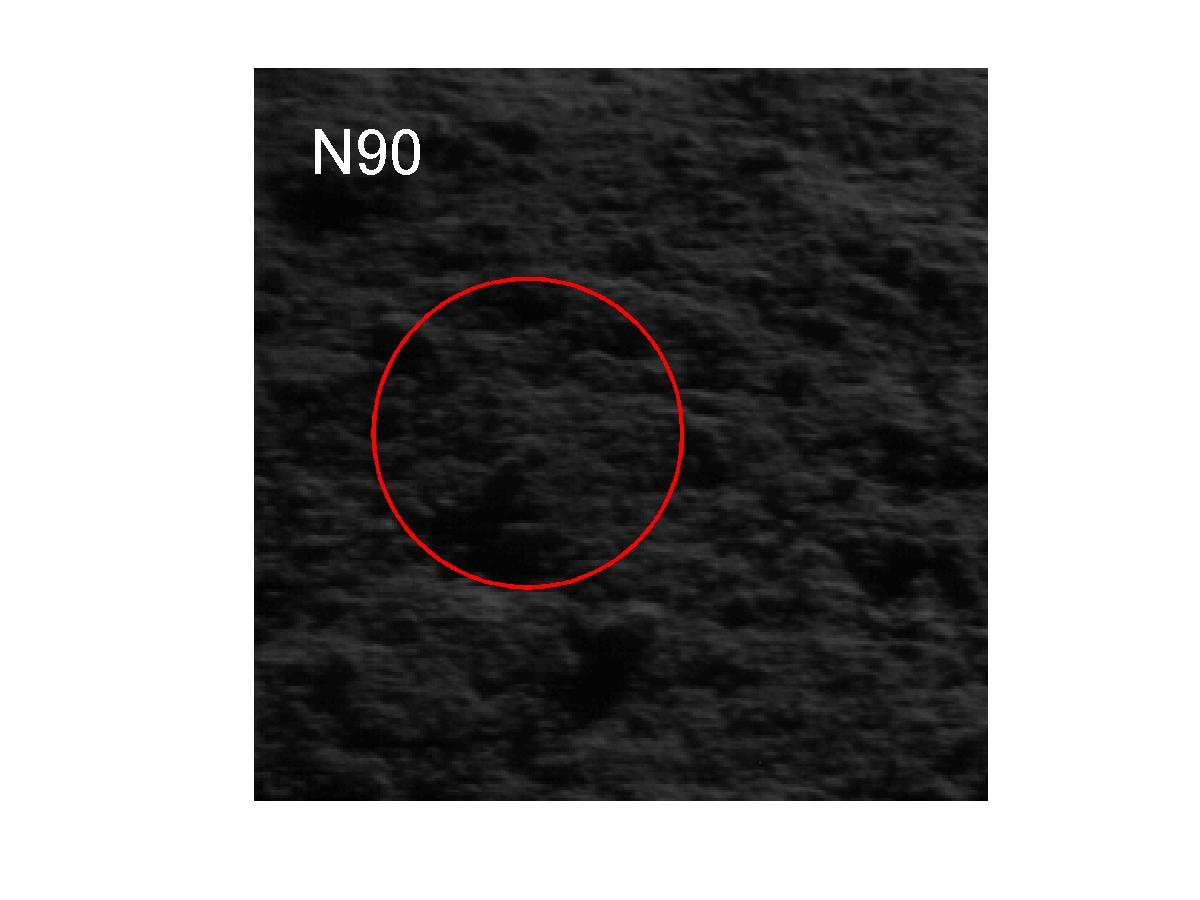

Supplement: Supplementary file 1 — Supplementary Information 1. [file 41598_2021_93694_MOESM1_ESM.zip › Supplementary_images of the spectral observations/N90.jpeg]

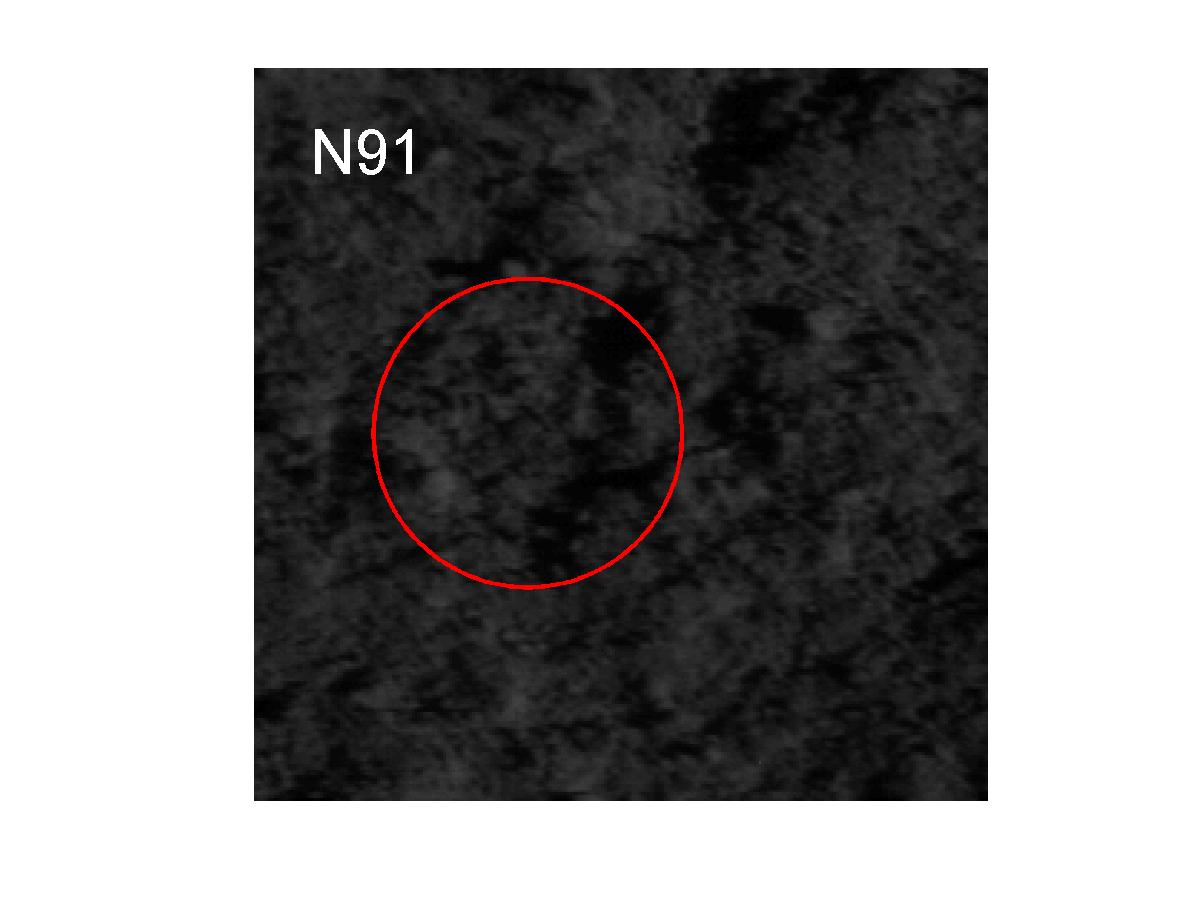

Supplement: Supplementary file 1 — Supplementary Information 1. [file 41598_2021_93694_MOESM1_ESM.zip › Supplementary_images of the spectral observations/N91.jpeg]

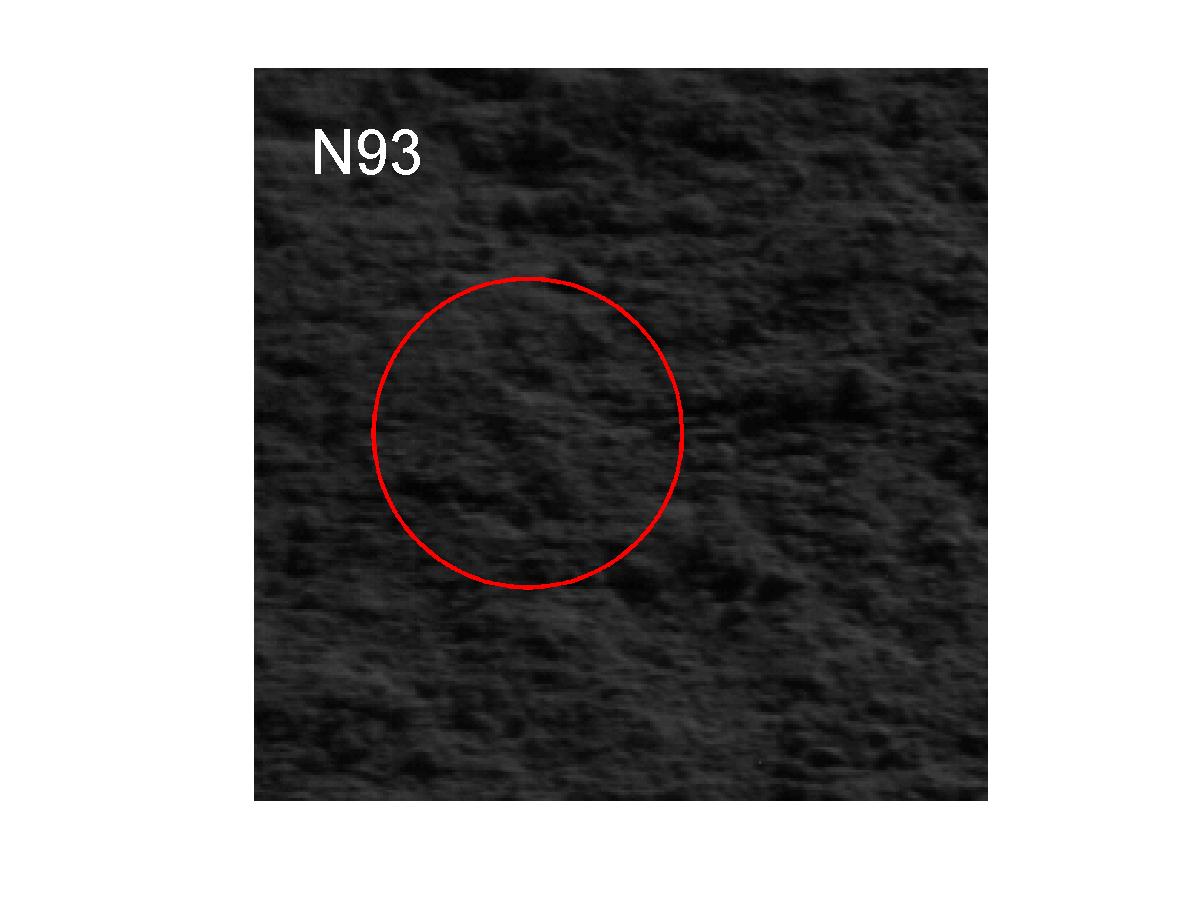

Supplement: Supplementary file 1 — Supplementary Information 1. [file 41598_2021_93694_MOESM1_ESM.zip › Supplementary_images of the spectral observations/N93.jpeg]

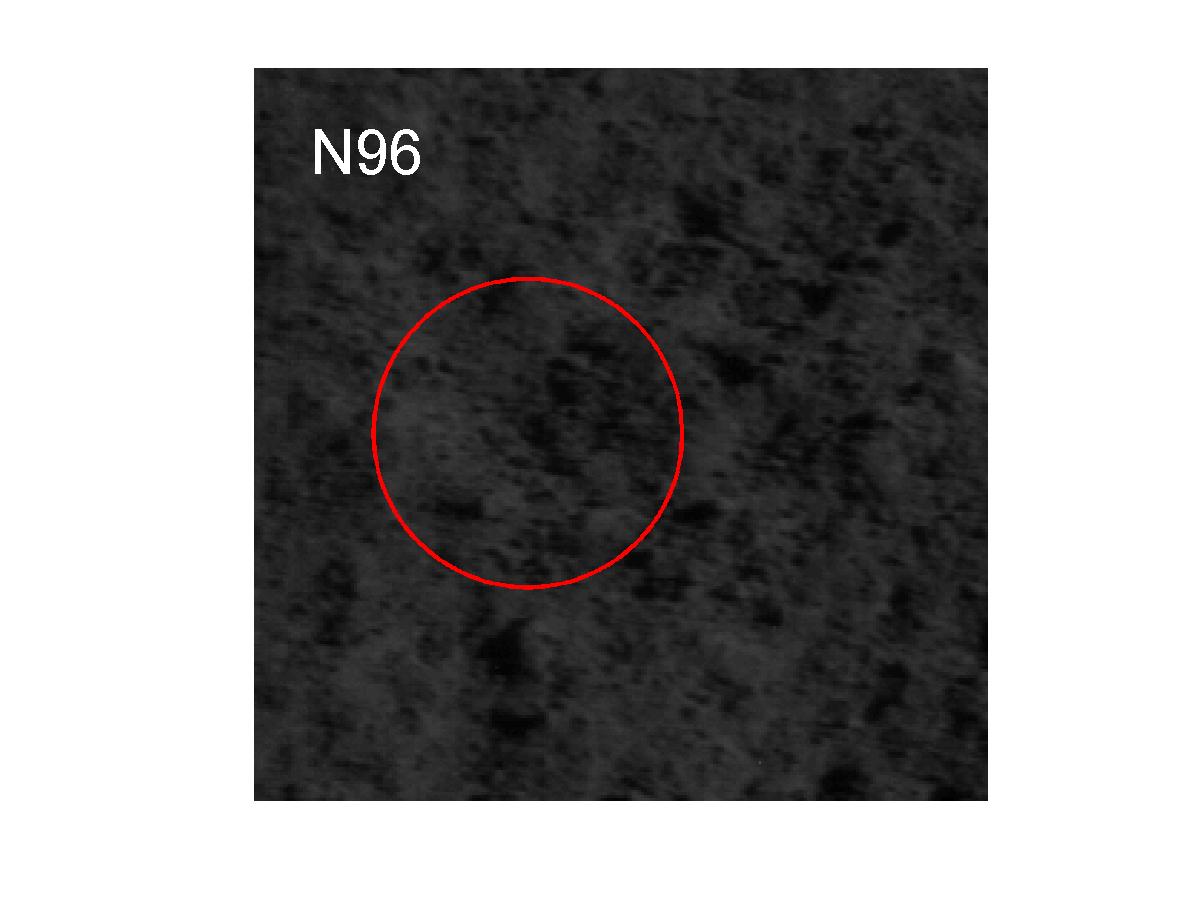

Supplement: Supplementary file 1 — Supplementary Information 1. [file 41598_2021_93694_MOESM1_ESM.zip › Supplementary_images of the spectral observations/N96.jpeg]

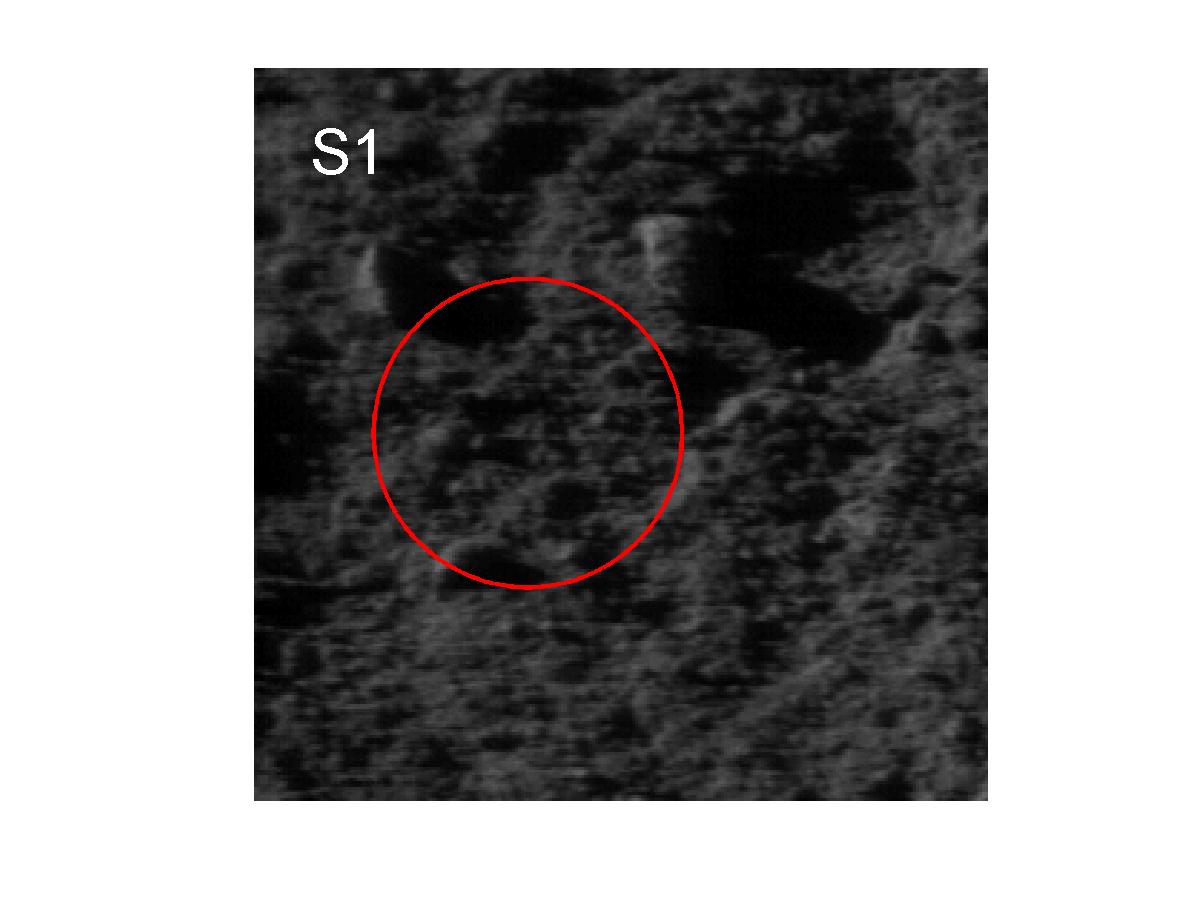

Supplement: Supplementary file 1 — Supplementary Information 1. [file 41598_2021_93694_MOESM1_ESM.zip › Supplementary_images of the spectral observations/S1.jpeg]
