# Supplementary material for: Mineralogical and chemical properties inversed from 21-lunar-day VNIS observations taken during the Chang’E-4 mission
Source: Sci Rep. 2021 Jul 29;11:15435. doi: 10.1038/s41598-021-93694-8 (PMC8322253; doi:10.1038/s41598-021-93694-8)
Supplement: Supplementary file 2 — Supplementary Information 2. [file 41598_2021_93694_MOESM2_ESM.docx]

Supporting Information for

[The mineralogy and chemical properties inversion from the 21-lunar-day VNIS observations of the Chang’E-4 mission]

[Qinghong Zeng 1, Shengbo Chen 1, 2 ✉, Yuanzhi Zhang 3, Yongling Mu 1,Rui Dai 1, Congyu Yang 1, Anzhen Li 1]

[^1^ College of Geo-exploration Science and Technology, Jilin University, Changchun, China.

^2^ 2CAS Center for Excellence in Comparative Planetology, Hefei, China.

^3^ Key Laboratory of Lunar and Deep-Space Exploration, National Astronomical Observatories, Chinese Academy of Sciences, Beijing, China.

Corresponding author: Shengbo Chen ([chensb@jlu.edu.](mailto:chensb@jlu.edu.)cn)]

**Introduction**

This supplemental material includes:

Text S1: Hapke radiative transfer model theory used in this study;

Text S2: Different types of pits along the path of the Yutu-2;

Text S3: FeO and $\mathrm{Ti}O_{2}$ content achieved from the VNIS spectra;

Text S4. Comparisons between modeled and measured reflectance spectra;

Text S5. The different spectral ranges used for polynomial fitting to calculate the band center;

Text S6. The VNIS spectra used in this study

References for supplementary materials.

Text S1. Hapke radiative transfer model theory used in this study

In this study, we use a Hapke radiative transfer model (Hapke, 1981) to estimate the lunar surface mineral abundance, because it has advantages in taking physical factors onto account on planetary surfaces. Lunar samples (Li et al., 2011) and meteorites (Li et al., 2015) was tested and applied using Hapke model, results showed the mineral abundance errors (absolute error) are commonly within 5–15%.

With the assumptions that the materials are mixed intimately and the particle size of the mineral is much larger than the spectral wavelength, the Hapke model (Hapke, 1981) can be written as:

$r\left( i,e,\alpha\right)=\frac{\omega_{ave}}{4(\mu_{0}+\mu)}\left\{ \left[ 1+B\left( \alpha\right) \right]P\left( \alpha\right)+H\left( \mu_{0},\omega_{ave} \right)H\left( \mu,\omega_{ave} \right)-1 \right\}$ (S1)

where $r$ is the reflectance, $\mu_{0}$ and$\mu$ are the cosines of the incidence angles (i) and emission angles (e), respectively, and $\alpha$ is phase angle.

$\omega_{ave}$ is the average single scattering albedo (SSA) of the material, which can be computed as:

$\omega_{ave}=\sum_{i} \frac{M_{i}\omega_{i}}{\rho_{I}d_{i}}/\sum_{i} \frac{M_{i}}{\rho_{I}d_{i}}$ (S2)

Where i denotes the ith component, $M_{i}$, $\rho_{I}$, $d_{i}$ and $\omega_{i}$ are mass fraction, density, particle size and single-scattering albedo of ith components, respectively.

$B\left( \alpha\right)$ is the backscattering function that describes opposition effect:

$B\left( g \right)=\frac{1}{1+\frac{tan(\alpha/2)}{h}} , h=-\frac{3}{8}ln(1-\varphi)$ (S3)

where, $\varphi$ is filling factor, which can be set to be 0.41 for the lunar regolith in this study (Bowell et al., 1989).

$P\left( \alpha\right)$ is the single-particle phase function, which can be approximated by Legendre polynomials:

$P\left( \alpha\right)=1+bP_{1}\left( \alpha\right)+cP_{2}\left( \alpha\right)$

$P_{1}\left( \alpha\right)=cos(\alpha)$ (S4)

$P_{2}\left( \alpha\right)=\frac{3}{2}{cos}^{2}\left( \alpha\right)-\frac{1}{2}$

where b and c are set as −0.4 and 0.25, respectively (Mustard et al.,1989).

H is a multiple-scattering function (Hapke, 2012):

$H\left( x,y \right)={\{1-(1-\gamma)x[r_{0}+(1-\frac{r_{0}}{2}-r_{0}x)ln((1+x)/x)]\}}^{-1}$ (S5)

Where $x$ is the cosine of the emission angle or the incident angle, y is the average single scattering albedo and $\gamma=\sqrt{1-y}$ ,$r_{0}=(1-\gamma)/(1+\gamma)$.

***Single-Scattering Albedo of Mineral Endmember***

The endmembers used in this study are high-calcium pyroxene (HCP), low-calcium pyroxene (LCP), olivine (OL), plagioclase (Plag) and agglutinates(Agg), which are the dominant minerals of the Moon. We only focus on the relative fractions of these minerals. For laboratory endmembers, the single-scattering albedos should be derived using mineral optical constants. Single-scattering albedos of each endmember can be calculated using the Hapke model for given optical constants and grain size (Hapke, 1981):

$\omega=S_{e}+(1-S_{e})\frac{(1-S_{i})\theta}{1-S_{i}\theta}$ (S6)

$S_{e}=\frac{{(n-1)}^{2}+k^{2}}{{(n+1)}^{2}+k^{2}}+0.05$ (S7)

$S_{i}=1-\frac{4}{n{(n+1)}^{2}}$ (S8)

$\theta=e^{-\alpha<D>}$ (S9)

where, n and k are optical constants of the minerals. $<D>$ is the average distance traveled by transmitted rays during one traverse of a particle:

$<D>=\frac{2}{3}\left[ n^{2}-\frac{1}{n}{(n^{2}-1)}^{3/2} \right]D$ (S10)

where, D is particle size.

$\alpha=4\pi nk/\lambda$ is the absorption coefficient of the mineral.

**Table S1** The reflectance spectra for endmembers used in this study

| **Endmember** | **Spectra Label** | **Size Rang (um)** | **Used Size (um)** | **n** |
| --- | --- | --- | --- | --- |
| HCP | LS-CMP-009 | 0-250 | 20 | 1.73 |
| OL | LS-CMP-006 | 250-500 | 20 | 1.83 |
| LCP | LS-CMP-012 | 0-250 | 25 | 1.77 |
| Plag | LS-CMP-086 | 0-25 | 11 | 1.56 |
| Agg | LU-CMP-007-1 | 100-1000 | 110 | 1.49 |

**Table S2** The accuracy of the mineral abundances estimation assessing by multi-measurement group during the tenth lunar day

| **Observation** | **Agg** | **HCP** | **OL** | **LCP** | **Plag** |
| --- | --- | --- | --- | --- | --- |
| N79 | 53.1 | 17.3 | 5.8 | 14.4 | 10.4 |
| N80 | 51.0 | 17.0 | 5.5 | 15.2 | 12.0 |
| N81 | 49.5 | 17.6 | 5.7 | 15.1 | 12.7 |
| N82 | 47.8 | 18.4 | 5.2 | 15.8 | 13.9 |
| N83 | 45.9 | 19.6 | 4.8 | 16.0 | 14.5 |
| N84 | 49.8 | 18.0 | 4.5 | 15.0 | 13.2 |
| N85 | 56.1 | 16.1 | 5.5 | 13.2 | 9.7 |
| N86 | 55.7 | 16.2 | 5.2 | 13.5 | 10.2 |
| N87 | 48.2 | 18.2 | 4.8 | 15.3 | 14.1 |
| N88 | 53.5 | 18.1 | 4.5 | 13.3 | 11.5 |
| N89 | 59.1 | 15.6 | 5.3 | 12.4 | 8.4 |
| N90 | 51.9 | 19.2 | 3.2 | 13.6 | 13.2 |
| average | 51.8 | 17.6 | 5.0 | 14.4 | 12.0 |
| SD | 3.71 | 1.18 | 0.68 | 1.10 | 1.88 |
| SD% | 7.16 | 6.69 | 13.66 | 7.65 | 15.67 |

The observations (N79-N90) are the multiple spectral measurements for the same target, the standard deviations (SD) is calculated, and the SD% is the standard deviation divided by the average.

Text S2. Different types of pits along the path of the Yutu-2

There are different types of pits along the path of the Yutu-2, the fragments filling in the pits show a different degree of space weathering (Fig.S1), providing unique view to understanding the space weathering influence on the mineral abundance by in-situ spectra on the far side of moon.


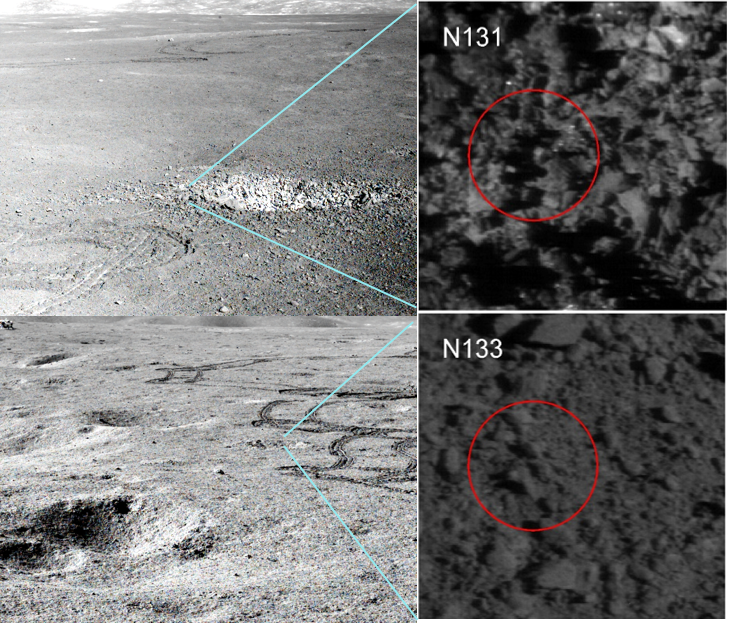


**Figure. S1** The different types of pits along the path of the Yutu-2, the fragments filling in the pits show a different degree of space weathering. The left column is the panoramic camera (PCAM) images of typical pits along the path, and the right column is the VNIS images at this site, the red circle is the field of view (FOV) for SWIR. PCAM images in the left column were created by our own script in Anaconda Python 3.7 (https://docs.anaconda.com/anaconda/install/). VNIS images in the right column were created by the script in MATLAB 2013a (https://www.mathworks.com/products/matlab.html).

Text S3. FeO and $\mathbf{Ti}\boldsymbol{O}_{\mathbf{2}}$ content achieved from the VNIS spectra

The empirical inversion of FeO relies on a plot of the NIR/VIS ratio versus the VIS reflectance. The key to the iron parameter qFe, the location of the “hypermature” end-member, was selected according to the suggestion by Lucey et al. [2000], i.e., maximizing the correlation between $\boldsymbol{\theta}_{\boldsymbol{Fe}}$ and the FeO content of the lunar soils and minimizing the residual effects of maturity. We used the similar origin as that used by Lucey et al. [2000].

The FeO contents of the lunar soils correlates with the reflectance ratio between bands at 750nm and 950nm via FeO parameter ($\boldsymbol{\theta}_{\boldsymbol{Fe}}$). The best fit cure of $\mathbf{FeO}\left( \boldsymbol{wt.\%} \right)\mathbf{=31.875}\boldsymbol{\theta}_{\boldsymbol{Fe}}\boldsymbol{-17.895}$ (Fig. S2a), where $\boldsymbol{\theta}_{\boldsymbol{Fe}}$ is defined by $\boldsymbol{\theta}_{\boldsymbol{Fe}}\boldsymbol{=-}\mathbf{arctan[}\boldsymbol{(}\boldsymbol{R}_{\boldsymbol{950}}\boldsymbol{/}\boldsymbol{R}_{\boldsymbol{750}}\boldsymbol{-}\boldsymbol{y}_{\boldsymbol{0}\boldsymbol{Fe}}\boldsymbol{)/(}\boldsymbol{R}_{\boldsymbol{750}}\boldsymbol{-}\boldsymbol{x}_{\boldsymbol{0}\boldsymbol{Fe}}\boldsymbol{)}$, and the origin ${\mathbf{(}\boldsymbol{x}}_{\mathbf{0}\boldsymbol{Fe}}\mathbf{,}\boldsymbol{y}_{\mathbf{0}\boldsymbol{Fe}}\mathbf{)}$ was set to (-0.1, 1.39).

Similarly, the $\mathbf{Ti}\boldsymbol{O}_{\mathbf{2}}$ contents are determined from the best fit curve of $\mathbf{Ti}\mathbf{O}_{\mathbf{2}} \left( \boldsymbol{wt}\boldsymbol{.\%} \right)\boldsymbol{=1.836\times}{\boldsymbol{\theta}_{\boldsymbol{Ti}}}^{\mathbf{9.779}}\mathbf{]}$ (Fig. S2b), where $\boldsymbol{\theta}_{\boldsymbol{Ti}}$ is defined by $\boldsymbol{\theta}_{\boldsymbol{Ti}}\mathbf{=arctan[(}\boldsymbol{R}_{\mathbf{415}}\mathbf{/}\boldsymbol{R}_{\mathbf{750}}\mathbf{-}\boldsymbol{y}_{\mathbf{0}\boldsymbol{Ti}}\mathbf{)/(}\boldsymbol{R}_{\mathbf{750}}\mathbf{-}\boldsymbol{x}_{\mathbf{0}\boldsymbol{Ti}}\mathbf{)]}$, and the origin $\mathbf{(}\boldsymbol{x}_{\mathbf{0}\boldsymbol{Ti}}\mathbf{,}\boldsymbol{y}_{\mathbf{0}\boldsymbol{Ti}}$) was set to (-1.08, 0.208). The $\boldsymbol{R}_{\mathbf{415}}$ was determined by $\boldsymbol{R}_{\mathbf{415}}\mathbf{=}{\mathbf{0.95}\boldsymbol{R}}_{\mathbf{415}}\mathbf{-0.0013}$ ($\boldsymbol{R}^{\mathbf{2}}\mathbf{=0.99}$).


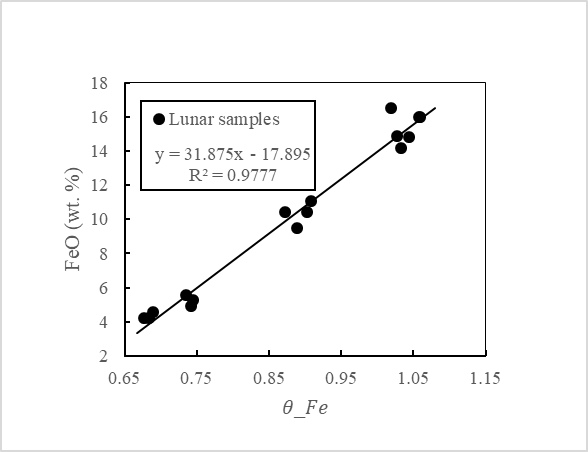

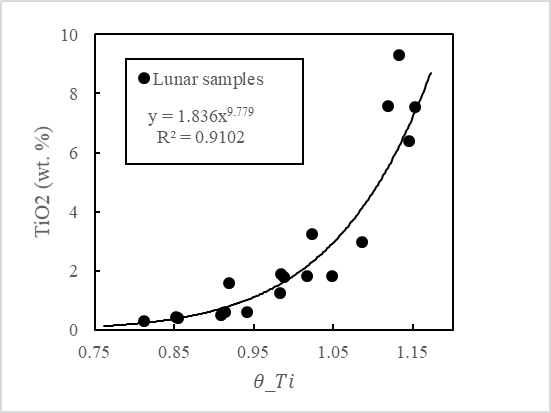


**Figure. S2** The plot of FeO and $\mathrm{Ti}O_{2}$ content measured for returned lunar samples versus the spectral iron and titanium parameters. The spectra of lunar samples are measured in laboratory (Pieters et al., 2000; Taylor et al., 2001).

Text S4. Comparisons between modeled and measured reflectance spectra


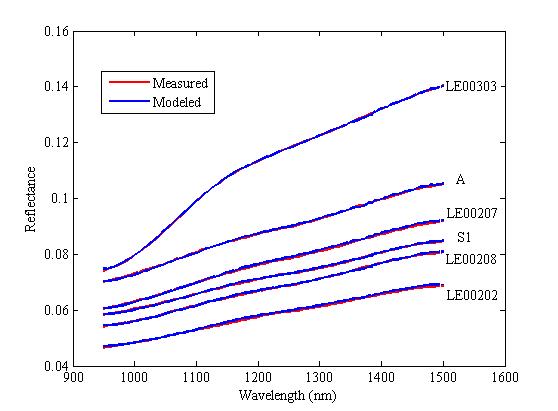


**Figure. S3** Comparisons between modeled and measured reflectance spectra of the rock (LE00303) and regolith.

Text S5. The different spectral ranges used for polynomial fitting to calculate the band center


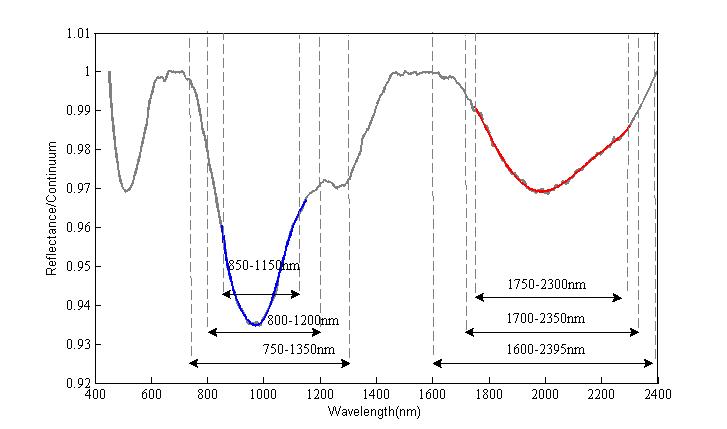


**Figure. S4 The illustration of the different spectral ranges used for polynomial fitting to calculate the band center.**

Text S6. The VNIS spectra used in this study

CE-4 VNIS data IDs are listed in Supplementary Table 3. And the VNIS image are obtained in the Zip file. “ Supplementary_images of the spectral observations.zip”

**Table S3.** Data IDs for CE-4 measurements used in this work.

| **No.** | **Measurement site** | **CE-4 Data ID** |
| --- | --- | --- |
| 1 | A | CE4_GRAS_VNIS-SD_SCI_N_20190104004000_20190109213900_0001_B.2B CE4_GRAS_VNIS-VD_SCI_N_20190104004000_20190109213900_0001_B.2B |
| 2 | S1 | CE4_GRAS_VNIS-SD_SCI_N_20190111150201_20190112102100_0003_B.2B CE4_GRAS_VNIS-VD_SCI_N_20190111150201_20190112102100_0003_B.2B |
| 3 | LE00202 | CE4_GRAS_VNIS-SD_SCI_N_20190131080301_20190201054300_0005_B.2B CE4_GRAS_VNIS-VD_SCI_N_20190131080301_20190201054300_0005_B.2B |
| 4 | LE00207 | CE4_GRAS_VNIS-SD_SCI_N_20190209053101_20190209130800_0008_B.2B CE4_GRAS_VNIS-VD_SCI_N_20190209053101_20190209130800_0008_B.2B |
| 5 | LE00208 | CE4_GRAS_VNIS-SD_SCI_N_20190209130801_20190210033400_0009_B.2B CE4_GRAS_VNIS-VD_SCI_N_20190209130801_20190210033400_0009_B.2B |
| 6 | LE00303 | CE4_GRAS_VNIS-SD_SCI_N_20190302060001_20190302131100_0015_B.2B CE4_GRAS_VNIS-VD_SCI_N_20190302060001_20190302131100_0015_B.2B |
| 7 | LE00304 | CE4_GRAS_VNIS-SD_SCI_N_20190302131101_20190302160000_0016_B.2B CE4_GRAS_VNIS-VD_SCI_N_20190302131101_20190302160000_0016_B.2B |
| 8 | LE00306 | CE4_GRAS_VNIS-SD_SCI_N_20190310092701_20190311064400_0020_B.2B CE4_GRAS_VNIS-VD_SCI_N_20190310092701_20190311064400_0020_B.2B |
| 9 | LE00308 | CE4_GRAS_VNIS-SD_SCI_N_20190311064401_20190312052700_0021_B.2B CE4_GRAS_VNIS-VD_SCI_N_20190311064401_20190312052700_0021_B.2B |
| 10 | N24 | CE4_GRAS_VNIS-SD_SCI_N_20190331080001_20190331100000_0024_B.2B CE4_GRAS_VNIS-VD_SCI_N_20190331080001_20190331100000_0024_B.2B |
| 11 | N25 | CE4_GRAS_VNIS-SD_SCI_N_20190331100001_20190331120000_0025_B.2B CE4_GRAS_VNIS-VD_SCI_N_20190331100001_20190331120000_0025_B.2B |
| 12 | N26 | CE4_GRAS_VNIS-SD_SCI_N_20190331120001_20190331140000_0026_B.2B CE4_GRAS_VNIS-VD_SCI_N_20190331120001_20190331140000_0026_B.2B |
| 13 | N27 | CE4_GRAS_VNIS-SD_SCI_N_20190331140001_20190331160000_0027_B.2B CE4_GRAS_VNIS-VD_SCI_N_20190331140001_20190331160000_0027_B.2B |
| 14 | N28 | CE4_GRAS_VNIS-SD_SCI_N_20190331230001_20190401010000_0028_B.2B CE4_GRAS_VNIS-VD_SCI_N_20190331230001_20190401010000_0028_B.2B |
| 15 | N29 | CE4_GRAS_VNIS-SD_SCI_N_20190401010001_20190401040000_0029_B.2B CE4_GRAS_VNIS-VD_SCI_N_20190401010001_20190401040000_0029_B.2B |
| 16 | N31 | CE4_GRAS_VNIS-SD_SCI_N_20190409020001_20190409070000_0031_B.2B CE4_GRAS_VNIS-VD_SCI_N_20190409020001_20190409070000_0031_B.2B |
| 17 | N32 | CE4_GRAS_VNIS-SD_SCI_N_20190409110001_20190411050000_0032_B.2B CE4_GRAS_VNIS-VD_SCI_N_20190409110001_20190411050000_0032_B.2B |
| 18 | N34 | CE4_GRAS_VNIS-SD_SCI_N_20190430080001_20190501120000_0034_B.2B CE4_GRAS_VNIS-VD_SCI_N_20190430080001_20190501120000_0034_B.2B |
| 19 | N36 | CE4_GRAS_VNIS-SD_SCI_N_20190508070001_20190509073000_0036_B.2B CE4_GRAS_VNIS-VD_SCI_N_20190508070001_20190509073000_0036_B.2B |
| 20 | N38 | CE4_GRAS_VNIS-SD_SCI_N_20190509160001_20190510120000_0038_B.2B CE4_GRAS_VNIS-VD_SCI_N_20190509160001_20190510120000_0038_B.2B |
| 21 | N40 | CE4_GRAS_VNIS-SD_SCI_N_20190529070001_20190529100000_0040_B.2B CE4_GRAS_VNIS-VD_SCI_N_20190529070001_20190529100000_0040_B.2B |
| 22 | N41 | CE4_GRAS_VNIS-SD_SCI_N_20190529100001_20190529120000_0041_B.2B CE4_GRAS_VNIS-VD_SCI_N_20190529100001_20190529120000_0041_B.2B |
| 23 | N43 | CE4_GRAS_VNIS-SD_SCI_N_20190607030001_20190609140000_0043_B.2B CE4_GRAS_VNIS-VD_SCI_N_20190607030001_20190609140000_0043_B.2B |
| 24 | N47 | CE4_GRAS_VNIS-SD_SCI_N_20190706073001_20190707040000_0047_B.2B CE4_GRAS_VNIS-VD_SCI_N_20190706073001_20190707040000_0047_B.2B |
| 25 | N51 | CE4_GRAS_VNIS-SD_SCI_N_20190804050001_20190804080000_0051_B.2B CE4_GRAS_VNIS-VD_SCI_N_20190804050001_20190804080000_0051_B.2B |
| 26 | N52 | CE4_GRAS_VNIS-SD_SCI_N_20190805020001_20190805050000_0052_B.2B CE4_GRAS_VNIS-VD_SCI_N_20190805020001_20190805050000_0052_B.2B |
| 27 | N53 | CE4_GRAS_VNIS-SD_SCI_N_20190805050001_20190806120000_0053_B.2B CE4_GRAS_VNIS-VD_SCI_N_20190805050001_20190806120000_0053_B.2B |
| 28 | N55 | CE4_GRAS_VNIS-SD_SCI_N_20190825053001_20190825080000_0055_B.2B CE4_GRAS_VNIS-VD_SCI_N_20190825053001_20190825080000_0055_B.2B |
| 29 | N58 | CE4_GRAS_VNIS-SD_SCI_N_20190827000001_20190827020000_0058_B.2B CE4_GRAS_VNIS-VD_SCI_N_20190827000001_20190827020000_0058_B.2B |
| 30 | N59 | CE4_GRAS_VNIS-SD_SCI_N_20190827030001_20190827053000_0059_B.2B CE4_GRAS_VNIS-VD_SCI_N_20190827030001_20190827053000_0059_B.2B |
| 31 | N60 | CE4_GRAS_VNIS-SD_SCI_N_20190827060001_20190827100000_0060_B.2B CE4_GRAS_VNIS-VD_SCI_N_20190827060001_20190827100000_0060_B.2B |
| 32 | N62 | CE4_GRAS_VNIS-SD_SCI_N_20190903143001_20190903150000_0062_B.2B CE4_GRAS_VNIS-VD_SCI_N_20190903143001_20190903150000_0062_B.2B |
| 33 | N63 | CE4_GRAS_VNIS-SD_SCI_N_20190904010001_20190904040000_0063_B.2B CE4_GRAS_VNIS-VD_SCI_N_20190904010001_20190904040000_0063_B.2B |
| 34 | N64 | CE4_GRAS_VNIS-SD_SCI_N_20190904053001_20190904073000_0064_B.2B CE4_GRAS_VNIS-VD_SCI_N_20190904053001_20190904073000_0064_B.2B |
| 35 | N65 | CE4_GRAS_VNIS-SD_SCI_N_20190904093001_20190904113000_0065_B.2B CE4_GRAS_VNIS-VD_SCI_N_20190904093001_20190904113000_0065_B.2B |
| 36 | N66 | CE4_GRAS_VNIS-SD_SCI_N_20190904130001_20190904140000_0066_B.2B CE4_GRAS_VNIS-VD_SCI_N_20190904130001_20190904140000_0066_B.2B |
| 37 | N68 | CE4_GRAS_VNIS-SD_SCI_N_20190923090001_20190923110000_0068_B.2B CE4_GRAS_VNIS-VD_SCI_N_20190923090001_20190923110000_0068_B.2B |
| 38 | N69 | CE4_GRAS_VNIS-SD_SCI_N_20190923123001_20190923130000_0069_B.2B CE4_GRAS_VNIS-VD_SCI_N_20190923123001_20190923130000_0069_B.2B |
| 39 | N70 | CE4_GRAS_VNIS-SD_SCI_N_20190924021501_20190924035000_0070_B.2B CE4_GRAS_VNIS-VD_SCI_N_20190924021501_20190924035000_0070_B.2B |
| 40 | N71 | CE4_GRAS_VNIS-SD_SCI_N_20190924060001_20190924061500_0071_B.2B CE4_GRAS_VNIS-VD_SCI_N_20190924060001_20190924061500_0071_B.2B |
| 41 | N72 | CE4_GRAS_VNIS-SD_SCI_N_20190924073001_20190924080000_0072_B.2B CE4_GRAS_VNIS-VD_SCI_N_20190924073001_20190924080000_0072_B.2B |
| 42 | N73 | CE4_GRAS_VNIS-SD_SCI_N_20190924094501_20190924101000_0073_B.2B CE4_GRAS_VNIS-VD_SCI_N_20190924094501_20190924101000_0073_B.2B |
| 43 | N74 | CE4_GRAS_VNIS-SD_SCI_N_20190924113001_20190924115000_0074_B.2B CE4_GRAS_VNIS-VD_SCI_N_20190924113001_20190924115000_0074_B.2B |
| 44 | N75 | CE4_GRAS_VNIS-SD_SCI_N_20190925022001_20190925024000_0075_B.2B CE4_GRAS_VNIS-VD_SCI_N_20190925022001_20190925024000_0075_B.2B |
| 45 | N76 | CE4_GRAS_VNIS-SD_SCI_N_20190925030001_20190925054500_0076_B.2B CE4_GRAS_VNIS-VD_SCI_N_20190925030001_20190925054500_0076_B.2B |
| 46 | N77 | CE4_GRAS_VNIS-SD_SCI_N_20190925080001_20190925100000_0077_B.2B CE4_GRAS_VNIS-VD_SCI_N_20190925080001_20190925100000_0077_B.2B |
| 47 | N78 | CE4_GRAS_VNIS-SD_SCI_N_20190925110001_20190925113000_0078_B.2B CE4_GRAS_VNIS-VD_SCI_N_20190925110001_20190925113000_0078_B.2B |
| 48 | N79 | CE4_GRAS_VNIS-SD_SCI_N_20191002030001_20191002043000_0079_B.2B CE4_GRAS_VNIS-VD_SCI_N_20191002030001_20191002043000_0079_B.2B |
| 49 | N80 | CE4_GRAS_VNIS-SD_SCI_N_20191002052001_20191002054500_0080_B.2B CE4_GRAS_VNIS-VD_SCI_N_20191002052001_20191002054500_0080_B.2B |
| 50 | N81 | CE4_GRAS_VNIS-SD_SCI_N_20191002070001_20191002072000_0081_B.2B CE4_GRAS_VNIS-VD_SCI_N_20191002070001_20191002072000_0081_B.2B |
| 51 | N82 | CE4_GRAS_VNIS-SD_SCI_N_20191002083001_20191002090000_0082_B.2B CE4_GRAS_VNIS-VD_SCI_N_20191002083001_20191002090000_0082_B.2B |
| 52 | N83 | CE4_GRAS_VNIS-SD_SCI_N_20191002103001_20191002105000_0083_B.2B CE4_GRAS_VNIS-VD_SCI_N_20191002103001_20191002105000_0083_B.2B |
| 53 | N84 | CE4_GRAS_VNIS-SD_SCI_N_20191003012001_20191003014500_0084_B.2B CE4_GRAS_VNIS-VD_SCI_N_20191003012001_20191003014500_0084_B.2B |
| 54 | N85 | CE4_GRAS_VNIS-SD_SCI_N_20191003033001_20191003050000_0085_B.2B CE4_GRAS_VNIS-VD_SCI_N_20191003033001_20191003050000_0085_B.2B |
| 55 | N86 | CE4_GRAS_VNIS-SD_SCI_N_20191003051501_20191003053000_0086_B.2B CE4_GRAS_VNIS-VD_SCI_N_20191003051501_20191003053000_0086_B.2B |
| 56 | N87 | CE4_GRAS_VNIS-SD_SCI_N_20191003071001_20191003073000_0087_B.2B CE4_GRAS_VNIS-VD_SCI_N_20191003071001_20191003073000_0087_B.2B |
| 57 | N88 | CE4_GRAS_VNIS-SD_SCI_N_20191003090001_20191003092000_0088_B.2B CE4_GRAS_VNIS-VD_SCI_N_20191003090001_20191003092000_0088_B.2B |
| 58 | N89 | CE4_GRAS_VNIS-SD_SCI_N_20191003105001_20191003111000_0089_B.2B CE4_GRAS_VNIS-VD_SCI_N_20191003105001_20191003111000_0089_B.2B |
| 59 | N90 | CE4_GRAS_VNIS-SD_SCI_N_20191004010001_20191004020000_0090_B.2B CE4_GRAS_VNIS-VD_SCI_N_20191004010001_20191004020000_0090_B.2B |
| 60 | N91 | CE4_GRAS_VNIS-SD_SCI_N_20191023023001_20191024073000_0091_B.2B CE4_GRAS_VNIS-VD_SCI_N_20191023023001_20191024073000_0091_B.2B |
| 61 | N93 | CE4_GRAS_VNIS-SD_SCI_N_20191101040001_20191101060000_0093_B.2B CE4_GRAS_VNIS-VD_SCI_N_20191101040001_20191101060000_0093_B.2B |
| 62 | N96 | CE4_GRAS_VNIS-SD_SCI_N_20191130093001_20191130130000_0096_B.2B CE4_GRAS_VNIS-VD_SCI_N_20191130093001_20191130130000_0096_B.2B |
| 63 | N100 | CE4_GRAS_VNIS-SD_SCI_N_20191222043001_20191222081500_0100_B.2B CE4_GRAS_VNIS-VD_SCI_N_20191222043001_20191222081500_0100_B.2B |
| 64 | N101 | CE4_GRAS_VNIS-SD_SCI_N_20191222081501_20191222124000_0101_B.2B CE4_GRAS_VNIS-VD_SCI_N_20191222081501_20191222124000_0101_B.2B |
| 65 | N102 | CE4_GRAS_VNIS-SD_SCI_N_20191222124001_20191223083000_0102_B.2B CE4_GRAS_VNIS-VD_SCI_N_20191222124001_20191223083000_0102_B.2B |
| 66 | N103 | CE4_GRAS_VNIS-SD_SCI_N_20191230034001_20191230051000_0103_B.2B CE4_GRAS_VNIS-VD_SCI_N_20191230034001_20191230051000_0103_B.2B |
| 67 | N104 | CE4_GRAS_VNIS-SD_SCI_N_20191230062501_20191230074500_0104_B.2B CE4_GRAS_VNIS-VD_SCI_N_20191230062501_20191230074500_0104_B.2B |
| 68 | N105 | CE4_GRAS_VNIS-SD_SCI_N_20191230095001_20191230101000_0105_B.2B CE4_GRAS_VNIS-VD_SCI_N_20191230095001_20191230101000_0105_B.2B |
| 69 | N106 | CE4_GRAS_VNIS-SD_SCI_N_20191230105001_20191230124000_0106_B.2B CE4_GRAS_VNIS-VD_SCI_N_20191230105001_20191230124000_0106_B.2B |
| 70 | N107 | CE4_GRAS_VNIS-SD_SCI_N_20191230124501_20191230130500_0107_B.2B CE4_GRAS_VNIS-VD_SCI_N_20191230124501_20191230130500_0107_B.2B |
| 71 | N108 | CE4_GRAS_VNIS-SD_SCI_N_20191230143501_20191231062000_0108_B.2B CE4_GRAS_VNIS-VD_SCI_N_20191230143501_20191231062000_0108_B.2B |
| 72 | N109 | CE4_GRAS_VNIS-SD_SCI_N_20191231080001_20191231120000_0109_B.2B CE4_GRAS_VNIS-VD_SCI_N_20191231080001_20191231120000_0109_B.2B |
| 73 | N110 | CE4_GRAS_VNIS-SD_SCI_N_20191231120001_20191231123000_0110_B.2B CE4_GRAS_VNIS-VD_SCI_N_20191231120001_20191231123000_0110_B.2B |
| 74 | N113 | CE4_GRAS_VNIS-SD_SCI_N_20200121015001_20200121142500_0113_B.2B CE4_GRAS_VNIS-VD_SCI_N_20200121015001_20200121142500_0113_B.2B |
| 75 | N114 | CE4_GRAS_VNIS-SD_SCI_N_20200129035001_20200129090000_0114_B.2B CE4_GRAS_VNIS-VD_SCI_N_20200129035001_20200129090000_0114_B.2B |
| 76 | N120 | CE4_GRAS_VNIS-SD_SCI_N_20200219074001_20200220102000_0120_B.2B CE4_GRAS_VNIS-VD_SCI_N_20200219074001_20200220102000_0120_B.2B |
| 77 | N124 | CE4_GRAS_VNIS-SD_SCI_N_20200328024001_20200329102000_0124_B.2B CE4_GRAS_VNIS-VD_SCI_N_20200328024001_20200329102000_0124_B.2B |
| 78 | N125 | CE4_GRAS_VNIS-SD_SCI_N_20200417070501_20200418110000_0125_B.2B CE4_GRAS_VNIS-VD_SCI_N_20200417070501_20200418110000_0125_B.2B |
| 79 | N128 | CE4_GRAS_VNIS-SD_SCI_N_20200427043501_20200428095500_0128_B.2B CE4_GRAS_VNIS-VD_SCI_N_20200427043501_20200428095500_0128_B.2B |
| 80 | N129 | CE4_GRAS_VNIS-SD_SCI_N_20200615025001_20200616030000_0129_B.2B CE4_GRAS_VNIS-VD_SCI_N_20200615025001_20200616030000_0129_B.2B |
| 81 | N131 | CE4_GRAS_VNIS-SD_SCI_N_20200616090001_20200617031000_0131_B.2B CE4_GRAS_VNIS-VD_SCI_N_20200616090001_20200617031000_0131_B.2B |
| 82 | N132 | CE4_GRAS_VNIS-SD_SCI_N_20200617053001_20200617064500_0132_B.2B CE4_GRAS_VNIS-VD_SCI_N_20200617053001_20200617064500_0132_B.2B |
| 83 | N133 | CE4_GRAS_VNIS-SD_SCI_N_20200624090001_20200624103000_0133_B.2B CE4_GRAS_VNIS-VD_SCI_N_20200624090001_20200624103000_0133_B.2B |
| 84 | N136 | CE4_GRAS_VNIS-SD_SCI_N_20200716031001_20200716100000_0136_B.2B CE4_GRAS_VNIS-VD_SCI_N_20200716031001_20200716100000_0136_B.2B |
| 85 | N138 | CE4_GRAS_VNIS-SD_SCI_N_20200724030001_20200724045000_0138_B.2B CE4_GRAS_VNIS-VD_SCI_N_20200724030001_20200724045000_0138_B.2B |
| 86 | N142 | CE4_GRAS_VNIS-SD_SCI_N_20200822025501_20200822044500_0142_B.2B CE4_GRAS_VNIS-VD_SCI_N_20200822025501_20200822044500_0142_B.2B |

References for supplementary materials

1. Hapke, B., Bidirectional reflectance spectroscopy .1. Journal of Geophysical Research, 1981,.86, 3039-3054.
2. Li, S.; Li, L., Radiative transfer modeling for quantifying lunar surface minerals, particle size, and submicroscopic metallic Fe(Article). Journal of Geophysical Research E: Planets 2011,116 (9).
3. Li, S.; Milliken, R. E., Estimating the modal mineralogy of eucrite and diogenite meteorites using visible-near infrared reflectance spectroscopy. Meteoritics and Planetary Science 2015, 50 (11), 1821-1850.
4. Bowell, E.; Hapke, B.; Domingue, D.; Lumme, K.; Peltoniemi, J.; Harris, A. W., Application of photometric models to asteroids. 1988.
5. Mustard, J. F.; Pieters, C. M., Photometric phase functions of common geologic minerals and applications to quantitative analysis of mineral mixture reflectance spectra. Journal of Geophysical Research : Solid earth and planets, 1989, 94 (B10), 13619-13634.
6. Lucey, P. G.; Blewett, D. T.; Jolliff, B. L., Lunar iron and titanium abundance algorithms based on final processing of Clementine ultraviolet-visible images. JOURNAL OF GEOPHYSICAL RESEARCH 2000, 105 (E8), 20297-20305.
7. Pieters, C. M.; Taylor, L. A.; Noble, S. K.; Keller, L. P.; Hapke, B.; Morris, R. V.; Allen, C. C.; McKay, D. S.; Wentworth, S., Space weathering on airless bodies: Resolving a mystery with lunar samples. Meteorit Planet Sci 2000, 35 (5), 1101-1107.
8. Taylor, L. A.; Pieters, C. M.; Keller, L. P.; Morris, R. V.; McKay, D. S., Lunar Mare Soils: Space weathering and the major effects of surface-correlated nanophase Fe. Journal of Geophysical Research, 2001, 106 (E11), 27985-27999.
